# Calculating metalation in cells reveals CobW acquires Co^II^ for vitamin B_12_ biosynthesis while related proteins prefer Zn^II^

**DOI:** 10.1038/s41467-021-21479-8

**Published:** 2021-02-19

**Authors:** Tessa R. Young, Maria Alessandra Martini, Andrew W. Foster, Arthur Glasfeld, Deenah Osman, Richard J. Morton, Evelyne Deery, Martin J. Warren, Nigel J. Robinson

**Affiliations:** 1 Department of Biosciences, Durham University, Durham, UK; 2 Department of Chemistry, Durham University, Durham, UK; 3 Max Planck Institute for Chemical Energy Conversion, Mülheim an der Ruhr, Germany; 4 Chemistry Department, Division of Mathematical and Natural Sciences, Reed College, Portland, OR USA; 5 Department of Mathematics, Physics, and Electrical Engineering, Northumbria University, Newcastle-upon-Tyne, UK; 6 School of Biosciences, University of Kent, Canterbury, Kent UK; 7 Quadram Institute Bioscience, Norwich Research Park, Norfolk, UK

**Keywords:** Proteins, Chemical tools, Metals

## Abstract

Protein metal-occupancy (metalation) in vivo has been elusive. To address this challenge, the available free energies of metals have recently been determined from the responses of metal sensors. Here, we use these free energy values to develop a metalation-calculator which accounts for inter-metal competition and changing metal-availabilities inside cells. We use the calculator to understand the function and mechanism of GTPase CobW, a predicted Co^II^-chaperone for vitamin B_12_. Upon binding nucleotide (GTP) and Mg^II^, CobW assembles a high-affinity site that can obtain Co^II^ or Zn^II^ from the intracellular milieu. In idealised cells with sensors at the mid-points of their responses, competition within the cytosol enables Co^II^ to outcompete Zn^II^ for binding CobW. Thus, Co^II^ is the cognate metal. However, after growth in different [Co^II^], Co^II^-occupancy ranges from 10 to 97% which matches CobW-dependent B_12_ synthesis. The calculator also reveals that related GTPases with comparable Zn^II^ affinities to CobW, preferentially acquire Zn^II^ due to their relatively weaker Co^II^ affinities. The calculator is made available here for use with other proteins.

## Abstract (web summary)

The connection between metal binding to proteins and availabilities of different metals in cells has been unclear. Here, the authors report a metalation-calculator that takes into account competition between metals and their variable accessibility, and thereby elucidate in vivo metal occupancies of three different proteins, CobW, YeiR and YjiA.

## Introduction

Paradoxically, in vitro, most metalloproteins prefer to bind incorrect metals^1,2^. A non-cognate metal may bind more tightly to the native site or bind by using a subset of the native ligands, by recruiting additional ligand(s) and/or by distorting the geometry of a binding site. Some enzymes are cambialistic and can function with alternative metals^3^, but more commonly a non-cognate metal inactivates an enzyme^4,5^. Correct metalation occurs in vivo because cells control the availability of metals to nascent proteins^1,6–8^. For example, specialised delivery proteins support metal acquisition by about a third of metalloproteins, (which in turn represent about a third of all proteins and about a half of all enzymes)^1,8^. However, metal delivery proteins do not ultimately solve the challenge of metalation because now the correct metal must somehow partition onto the delivery protein.

For metalloproteins generally, there is a need to relate metal binding to the intracellular availability of metals. Our recent work provides the basis for such contextualisation^9^. Cells are thought to assist protein metalation by maintaining availabilities to the opposite of the Irving-Williams series with weaker binding metals such as Mg^II^, Mn^II^ and Fe^II^ highly available and tighter binding metals such as Ni^II^, Zn^II^ and Cu^I^ at low availabilities^10–12^. We have demonstrated this to be correct by determining the sensitivities of the DNA-binding metal-sensing transcriptional regulators of *Salmonella enterica* serovar Typhimurium (hereafter *Salmonella*)^9^. The sensors trigger expression of genes whose products, for example, import metals that are deficient or export those in excess^6,13^. A collection of thermodynamic parameters were measured for each sensor and used to calculate the (dynamic range of) buffered intracellular metal concentrations to which each sensor is finely tuned to switch gene expression^9,14^. For the more competitive metals, detection is so sensitive as to suggest that there is no hydrated metal in the cell^9,10^. Instead, rapid associative metal-exchange can occur between labile ligands in the crowded cytosol and the binding sites of metalloproteins, making it unhelpful to express metal availabilities as concentrations of the (largely irrelevant and negligible) hydrated species: thus, the chemical potentials of the bound available metals were expressed as free energies Δ*G*^9^. It is hypothesised that metal-delivery proteins acquire their metals from these exchangeable, buffered pools. By reference to available Δ*G* values and by assuming an idealised cell in which the sensors are at the mid-points of their dynamic ranges, the correct metal (Co^II^) was previously predicted to partition to the known chelatase of the anaerobic cobalamin biosynthetic pathway, CbiK^9^. There is a need to build upon this approach to account for (1) multiple competing metals and (2) non-idealised (conditional) cell cultures in order to understand the actions of putative metal delivery proteins (such as CobW and related GTPases) and to simplify such calculations for general use.

The G3E GTPase superfamily contains two branches of delivery proteins involved in the assembly of Ni^II^ centres (HypB, UreG), one for handling the cobalamin cofactor (MeaB), plus a fourth family, COG0523, investigated here^15,16^. Though ubiquitous, from bacteria to plants and humans, members of COG0523 have been persistently enigmatic^16^. Gene context and informatics have linked subgroups of the COG0523 family to at least three different metals: these include Nha3 associated with Fe^III^-requiring nitrile hydratases^17–19^, various subgroups (including YeiR, ZigA and ZagA) implicated in Zn^II^ metallostasis^16,20–24^, and CobW associated with the aerobic biosynthesis of cobalamin (vitamin B_12_) and hence Co^II^ (ref. ^25^). Metal insertion into the preformed corrin ring in the aerobic pathway for vitamin B_12_ biosynthesis appears to be irreversible^26,27^, highlighting the importance of Co^II^ specificity at this step. Disruption of *cobW* impairs B_12_ biosynthesis^25^, and a role in Co^II^ delivery has been suggested^28^, but not established. The roles of YeiR and YjiA (two homologues of CobW in *Salmonella*) are undefined, albeit Zn^II^ has been predicted for YeiR^16,20^, and Co^II^, Ni^II^ and Zn^II^ shown to bind *Escherichia coli (E. coli)* YjiA in vitro^21^. The impact of GTP binding on metal binding remains to be tested for COG0523 GTPases.

Vitamin B_12_ is an essential nutrient for human health but is neither made nor required by plants^29^. Prokaryotes present in the ruminant microbiome produce B_12_ and hence meat and dairy products provide a dietary source^30^. Vitamin B_12_ supplements are recommended for those on a vegan diet and its biomanufacture is in demand^31^. *E. coli* has significant advantages over currently employed production strains^32^. Native *E. coli* does not make vitamin B_12_ but strains containing functional B_12_ pathways have been created: the most promising of these use genes of the aerobic pathway, primarily from *Rhodobacter capsulatus*, and produce high levels of metal-free corrinoids^33–35^. In *R. capsulatus*, Co^II^ is inserted into the corrin ring by a cobalt chelatase ATPase (CobNST)^36^, putatively via CobW^28^. An understanding of Co^II^-availability inside engineered *E. coli* strains (referred to hereafter as E. coli*) is required to optimise Co^II^ supply for the B_12_ pathway, with relevance to biomanufacturing. High B_12_ production coupled with similarity between the metal sensors of *E. coli* and *Salmonella* also make this system tractable for testing metalation in vivo: the metal sensors of *Salmonella* having been thermodynamically characterised^9^.

Here we calculate intracellular metalation to discover which metals partition onto three proposed metal delivery proteins (CobW, YeiR and YjiA). This work makes it widely possible to quantify metal occupancy of proteins and other molecules in vivo based on thermodynamic parameters. The cognate metals of proteins can thus be identified where this was uncertain, and the contributions of additional mechanisms that enable metalation (such as molecular interactions or bespoke growth conditions) exposed. We determine metal affinities of CobW, YeiR and YjiA, and calculate their in vivo metal occupancies (in *Salmonella* and closely related species), establishing that CobW cannot acquire Co^II^ from the intracellular milieu in the absence of Mg^II^GTP and revealing Zn^II^ as the preferred metal for Mg^II^GTP-YeiR and Mg^II^GTP-YjiA. Predictions of Co^II^ occupancy of Mg^II^GTP-CobW in Co^II^-supplemented media are reflected in CobW-dependent production of B_12_ in E. coli*, establishing the function of CobW in Co^II^-supply for B_12_ and further validating an easy-to-use metalation calculator.

## Results

### Guanine nucleotides create two metal sites in CobW

**Fig. 1:**
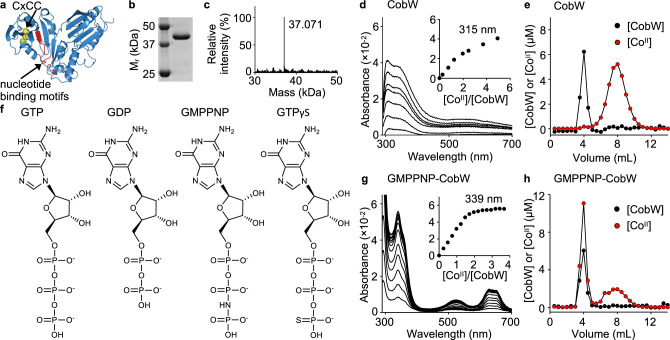
Co^II^ binding to CobW is enhanced by guanine nucleotides. **a** Homology model of CobW (generated with SWISS-MODEL^67^ using *E. coli* YjiA PDB entry 1NIJ^68^ as template; image generated using CCP4 Molecular Graphics software) showing sulfur atoms from conserved CxCC motif (in yellow) and nucleotide-binding (GxxGxGKT, hhhExxG, SKxD*) motifs^15,16^ (in red). *Ordinarily NKxD but [ST]KxD observed in some COG0523 proteins^15^. **b** Purified CobW analysed by SDS-PAGE (full image in Supplementary Fig. 1; *n* = 1 under these conditions). **c** ESI-MS analysis (de-convoluted spectra) of purified CobW. **d** Representative (*n* = 2) apo-subtracted spectra of Co^II^-titrated CobW (26.1 µM); feature at 315 nm (inset) shows a non-linear increase. **e** Representative (*n* = 2) elution profile following gel-filtration of a mixture of CobW (10 µM) and Co^II^ (30 µM) showing no co-migration of metal (red) with protein (black). Fractions were analysed for protein by Bradford assay and for metal by ICP-MS. **f** Structures of nucleotides used in this work (generated using ChemDraw software). **g** As in **d** for a mixture of CobW (24 µM) and GMPPNP (60 µM); feature at 339 nm (inset) showing a linear increase saturating at 2:1 ratio Co^II^:CobW (*n* = 2). **h** As in **e** for a mixture of CobW (10 µM), Co^II^ (30 µM) and GMPPNP (30 µM) showing co-migration of 1.8 equivalents Co^II^ per CobW (mean value from peak integration, *n* = 2 independent experiments). Data replicates are shown in Supplementary Fig. 1.

The first objective was to measure the Co^II^ affinities of the form of CobW that acquires metal inside a cell. A modelled structure of CobW (Fig. 1a) showed hypothetical nucleotide-binding sequences adjacent to a putative metal-binding motif, CxCC, and both of these features are conserved in the COG0523 subfamily^15,16^. To assess the effect of nucleotides on metal binding, CobW was overexpressed and purified (Fig. 1b and Supplementary Fig. 1). The protein mass determined by electrospray ionisation mass spectrometry (ESI-MS) (37,071 Da; Fig. 1c) is consistent with that expected for CobW after cleavage of the N-terminal methionine (37,072.6 Da).

Co^II^-titration of CobW alone (26.1 µM) produced a non-linear increase in absorbance at 315 nm (Fig. 1d) but gel-filtration of a mixture of CobW (10 µM) and Co^II^ (30 µM) resulted in their complete separation (Fig. 1e). Taken together, these results suggest only weak interactions between Co^II^ and CobW in the absence of cofactors. In the presence of excess GMPPNP (60 µM), a less readily hydrolysed analogue of GTP (Fig. 1f), Co^II^-titration of CobW (24 µM) produced an absorbance feature at 339 nm characteristic of ligand-to-metal charge transfer with an extinction coefficient (*ε* ~2800 M^−1^ cm^−1^) indicative of coordination by three cysteine side-chains^37^ (Fig. 1g). Visible absorbance features (500–700 nm, *ε* ~300–700 M^−1^ cm^−1^) are characteristic of *d–d* transitions, diagnostic of tetrahedral Co^II^-coordination geometry (Fig. 1g and Supplementary Fig. 2). Equivalent experiments performed with GTP and an alternate stable analogue, GTPγS, generated indistinguishable spectra (Supplementary Fig. 3a, b). These absorbance features increased linearly, saturating at 2:1 ratio Co^II^:CobW, and gel filtration of a mixture of CobW (10 µM) and Co^II^ (30 µM) pre-incubated with GMPPNP (30 µM) resulted in co-migration of ~2 equivalents Co^II^ per protein monomer (Fig. 1h). These data show that binding of guanine nucleotides to CobW promotes tight coordination of two metal ions.

### Cellular [Mg^II^] generates one Co^II^ site in nucleotide-bound CobW

**Fig. 2:**
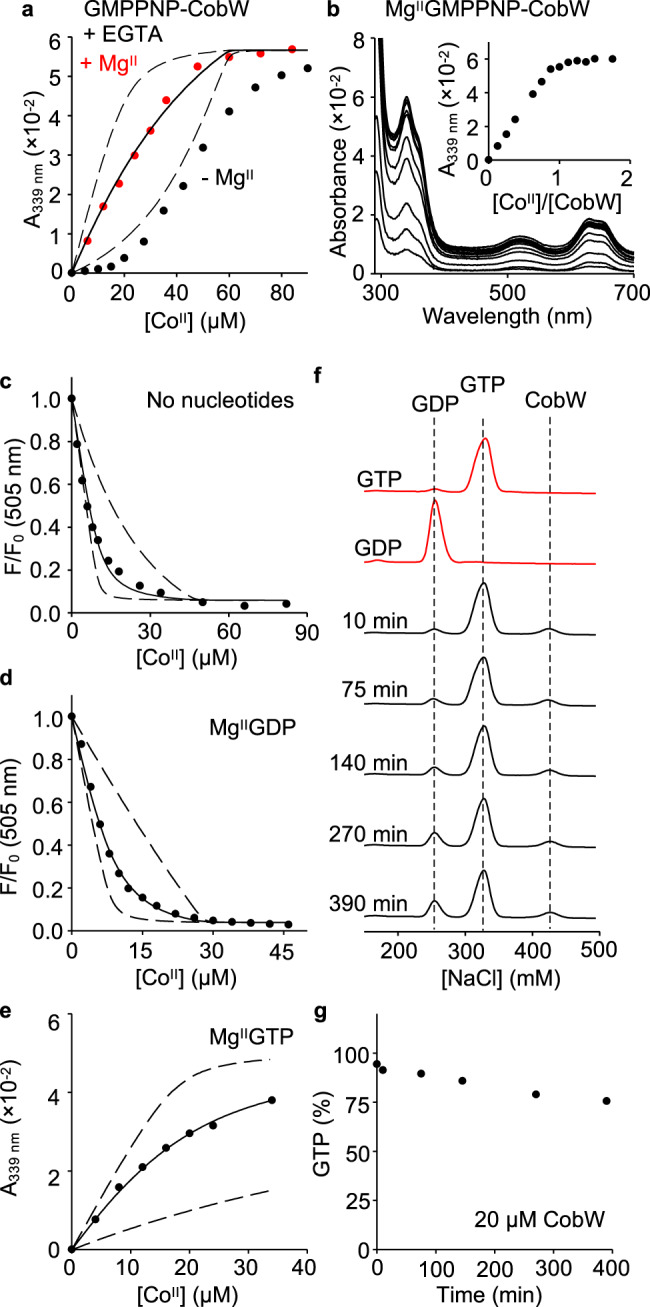
Mg^II^ and the γ-phosphate group of GTP are necessary for high affinity Co^II^ binding. **a** Absorbance (relative to Co^II^-free solution) of Co^II^-titrated CobW (20 µM) with GMPPNP (60 µM) in competition with EGTA (40 µM); titrations in the absence (black) or presence (red) of Mg^II^ (2.7 mM, i.e. concentration in a bacterium^9,12^). Data shown are representative of *n* = 3 independent experiments (with varying [competitor] and/or identity; see Supplementary Figs. 3c, d and 4a, b). **b** Absorbance (relative to Co^II^-free solution) of Co^II^-titrated CobW (20 µM) with GMPPNP (60 µM) and Mg^II^ (2.7 mM) in the absence of competing ligand; feature at 339 nm (inset) showing linear increase saturating at 1:1 ratio Co^II^:CobW (*n* = 2; see Supplementary Fig. 3e). **c**–**e** Representative *K*_Co(II)_ quantification for CobW in the absence or presence of nucleotides (*n* = 3 independent experiments, details in Supplementary Fig. 4 and Supplementary Table 1). **c** Fluorescence quenching of Co^II^-titrated fura-2 (10 µM) in competition with CobW alone (37 µM). **d** Fluorescence quenching of Co^II^-titrated fura-2 (8.1 µM) in competition with CobW (20 µM) with Mg^II^ (2.7 mM) and GDP (200 µM). **e** Absorbance (relative to Co^II^-free solution) of Co^II^-titrated CobW (18 µM) in competition with EGTA (2.0 mM) with Mg^II^ (2.7 mM) and GTP (200 µM). Solid traces in **a**, **c**, **d**, **e** show curve fits of experimental data to a model where CobW binds one molar equivalent Co^II^ per protein monomer. Dashed lines show simulated responses for *K*_Co(II)_ tenfold tighter or weaker than the fitted value. **f** Analysis of GTP hydrolysis by anion-exchange chromatography. Control samples of GTP and GDP elute as distinct peaks (red traces) measured by absorbance at 254 nm. Black traces show the extent of hydrolysis of GTP (200 µM) incubated with CobW (20 µM), Mg^II^ (2.7 mM) and Co^II^ (18 µM) over time. **g** Analysis of data from **f** showing % GTP remaining over time. After 390 min incubation, nucleotides remain primarily (>75 %) unhydrolysed. Equivalent data using 4:1 ratio GTP:CobW is shown in Supplementary Fig. 6.

The uniform absorbance increase observed across both metal-binding events in Fig. 1g could be explained by either the presence of two sequentially filled sites with identical spectroscopic features or two spectrally distinct sites being filled in a pairwise manner. Competition between GMPPNP-CobW and ethylene glycol tetraacetic acid (EGTA) or nitrilotriacetic acid (NTA) for Co^II^ produced sigmoidal binding isotherms indicating positive cooperativity (*K*_D2_ < *K*_D1_) between the two metal sites (Fig. 2a and Supplementary Fig. 3c, d). Such cooperativity will result in pairwise filling of the two metal sites. Given that GTPases typically bind nucleotides in complex with Mg^II^, we hypothesised that the cognate metal for the first (weak-affinity) site is Mg^II^, and that Mg^II^ binding triggers assembly of the second (tight-affinity) metal site in GMPPNP-CobW. Co^II^ titration of CobW (20 µM) with GMPPNP (60 µM) and Mg^II^ (2.7 mM, i.e. available idealised intracellular concentration, [Mg^II^]_cell_^9,12^) produced identical spectra to that observed without Mg^II^ but saturating at 1:1 ratio Co^II^:CobW (Fig. 2b and Supplementary Fig. 3e). Equivalent experiments performed with GTP and GTPγS also revealed 1:1 Co^II^:CobW stoichiometry in the presence of [Mg^II^]_cell_ (Supplementary Fig. 3f, g). Thus, binding of Mg^II^ and guanine nucleotides preassembles one distinct Co^II^ site in CobW. Occupancy of the first site by Mg^II^ was spectroscopically silent in these experiments. The features at 339 nm and at 500–700 nm therefore correspond exclusively to a distinct tetrahedral Co^II^ site and the coordinating sulfhydryl side-chains likely derive (at least in part) from the CxCC motif adjacent to the nucleotide-binding site.

Due to the tight coordination of Co^II^ to nucleotide-bound forms of CobW (i.e. no measurable dissociation at the micromolar-range protein concentrations required for detection), it was necessary to employ competition assays, whereby Co^II^ is partitioned between the protein and a ligand of well-matched and defined Co^II^ affinity, for reliable quantification of metal-binding affinities^38^. Competition between Mg^II^GMPPNP-CobW and EGTA for Co^II^ yielded a binding isotherm consistent with 1:1 stoichiometry for both Co^II^:protein and Co^II^:EGTA, and enabled *K*_Co(II)_ of 2.7 (±0.4) × 10^−9^ M for Mg^II^GMPPNP-CobW to be determined (Fig. 2a, Supplementary Fig. 4a, b and Supplementary Tables 1, 2). Mg^II^ had negligible impact on the conditional affinity of EGTA for Co^II^ at the concentrations used here (Supplementary Table 3): For this reason, Mg^II^ was not incorporated into curve-fitting models. Competition with EGTA revealed a Co^II^ affinity for Mg^II^GTPγS-CobW (*K*_Co(II)_ = 1.7 (±0.8) × 10^−10^ M; Supplementary Fig. 4c–e and Supplementary Tables 1, 2), that was more than tenfold tighter than Mg^II^GMPPNP-CobW, establishing that the nature of the bound nucleotide exerts an effect on metal binding to CobW.

### Co^II^ binds CobW 1000-fold tighter with GTP than GDP

Observed variation in Co^II^ affinities of CobW in association with Mg^II^GTPγS versus Mg^II^GMPPNP prompted us to assess the Co^II^ affinities of all three anticipated biological species: nucleotide-free CobW, Mg^II^GTP-CobW and Mg^II^GDP-CobW. Co^II^ affinities of CobW and Mg^II^GDP-CobW were determined via competition with the probe ligand fura-2 (Fig. 2c, d, Supplementary Fig. 4f–i and Supplementary Tables 1, 2), which undergoes fluorescence quenching upon Co^II^ binding^39^. Fura-2 is too weak to compete effectively with Mg^II^GTP-CobW (Supplementary Fig. 4j), but high concentrations of EGTA or NTA imposed sufficient competition to enable *K*_Co(II)_ of 3.0 (±0.8) × 10^−11^ M to be determined (Fig. 2e, Supplementary Fig. 4k–m and Supplementary Tables 1, 2). GTP concentration was not a limiting factor in these affinity measurements (Supplementary Fig. 5). Under identical conditions used for affinity measurements, we confirmed that CobW-catalysed GTP hydrolysis is sufficiently slow such that nucleotides remain predominantly unhydrolysed over the duration of metal-binding experiments (Fig. 2f, g and Supplementary Fig. 6). Mg^II^GDP-CobW, despite displaying identical absorbance features indicating the persistence of the cysteine-rich tetrahedral site (Supplementary Fig. 7), has a Co^II^ affinity more than 1000-fold weaker than Mg^II^GTP-CobW and only marginally tighter than unbound CobW which lacks this site altogether (Supplementary Table 2). GTP also confers higher Co^II^ affinity than either of the tested non-hydrolysable analogues in which the γ-phosphates have been modified (Fig. 1f and Supplementary Table 2). Thus, the presence of an intact nucleotide γ-phosphate is a prerequisite for high-affinity Co^II^ binding.

### Cu^I^ and Zn^II^ bind Mg^II^GTP-CobW more tightly than Co^II^

**Fig. 3:**
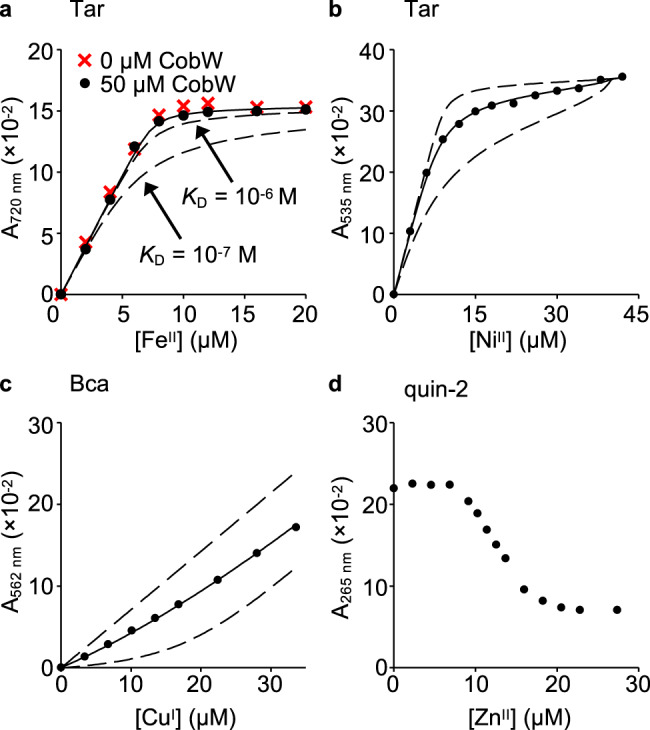
Binding of Mg^II^GTP-CobW to Fe^II^, Ni^II^, Cu^I^ and Zn^II^. **a** Absorbance upon Fe^II^ titration into a mixture of Tar (16 µM), Mg^II^ (2.7 mM) and GTP (500 µM) in the absence (red crosses) or presence (black circles) of CobW (50 µM). Dashed lines show simulated responses for specified *K*_Fe(II)_ of Mg^II^GTP-CobW, providing limiting *K*_Fe(II)_ ≥ 10^−6^ M. Control Fe^II^ titration into a solution of Tar (16 µM) in buffer only (Supplementary Fig. 8a) confirmed that Mg^II^ and GTP did not inhibit stoichiometric Fe^II^Tar_2_ formation. **b** Absorbance change (relative to Ni^II^-free solution) of Ni^II^-titrated Tar (20 µM) in competition with CobW (30 µM) in the presence of Mg^II^ (2.7 mM) and GTP (300 µM). **c** Absorbance of Cu^I^-titrated Bca (1.0 mM) in competition with CobW (20 µM) in the presence of Mg^II^ (2.7 mM) and GTP (200 µM). In **a**–**c**, solid traces show curve fits of experimental data to models where CobW binds one molar equivalent of metal per protein monomer. Supplementary Table 2 contains mean ± standard deviation (SD) *K*_metal_ values from *n* = 3 independent experiments (full details in Supplementary Figs. 8–12 and Supplementary Table 1). In **b**, **c**, dashed lines show simulated responses for *K*_metal_ tenfold tighter or weaker than the fitted value. **d** Absorbance (relative to probe-free solution) upon titration of Zn^II^ into a mixture of quin-2 (10 µM), Mg^II^ (2.7 mM), GTP (100 µM) and CobW (10 µM).

In view of the challenges associated with correct metal–protein speciation, we sought to determine Mg^II^GTP-CobW affinities for other first-row transition metals (Fe^II^, Ni^II^, Cu^I^, Zn^II^). Fe^II^-titration into a mixture of Mg^II^GTP-CobW (50 µM) and probe ligand 4-(2-thiazolylazo)-resorcinol (Tar) (16 µM) showed Fe^II^ being withheld by Tar which revealed a limiting affinity (*K*_Fe(II)_ > 10^−6^ M) for Mg^II^GTP-CobW (Fig. 3a, Supplementary Fig. 8 and Supplementary Tables 1, 2). Competition between Mg^II^GTP-CobW (10 µM) and mag-fura-2 (Mf2; 20 µM) for Ni^II^ showed that Mg^II^GTP-CobW has one Ni^II^ site which outcompetes Mf2 (*K*_Ni(II)_ < 10^−8^ M) in addition to two weaker sites which compete with Mf2 for Ni^II^ (*K*_Ni(II)_ ~10^−7^ M) and are also present in CobW alone (Supplementary Fig. 9a). Competition with Tar allowed the affinity of the tight Ni^II^ site in Mg^II^GTP-CobW to be determined (*K*_Ni(II)_ = 9.8 (±6.5) × 10^−10^ M; Fig. 3b, Supplementary Fig. 9b, c and Supplementary Tables 1, 2). The conditional *β*_2_ value (4.3 (±0.6) × 10^15^ M^−2^) for Ni^II^Tar_2_ formation under experimental conditions (pH 7.0, 100 mM NaCl, 400 mM KCl) was independently established by competition with EGTA (Supplementary Fig. 10). Titration of Mg^II^GTP-CobW (15 µM) and bathocuproine disulfonate (Bcs; 30 µM) with Cu^I^ did not reach the expected intensity at saturating metal concentrations (Supplementary Fig. 11a) suggesting the presence of a stable ternary complex, which would preclude accurate affinity determinations^40^. An equivalent experiment with alternative Cu^I^-probe bicinchoninic acid (Bca) showed that Mg^II^GTP-CobW has two Cu^I^ sites which outcompete Bca and at least three additional weaker Cu^I^ sites which effectively compete with the probe (Supplementary Fig. 11b). Effective competition imposed by excess Bca enabled *K*_Cu(I)_ of 2.4 (±0.9) × 10^−16^ M to be determined (Fig. 3c, Supplementary Figs. 11c, d,  12 and Supplementary Tables 1, 2), assuming only the tightest Cu^I^ site can acquire metal at the limiting Cu^I^ availabilities employed (e.g. [Cu^I^_aq_] <3 × 10^−16^ M in Fig. 3c). Zn^II^ titration into a mixture of quin-2 (10 µM) and Mg^II^GTP-CobW (10 µM) revealed one high-affinity Zn^II^ site in the protein which was too tight to be quantified by using quin-2 thus showing *K*_Zn(II)_ < 10^−12^ M (Fig. 3d).

**Fig. 4:**
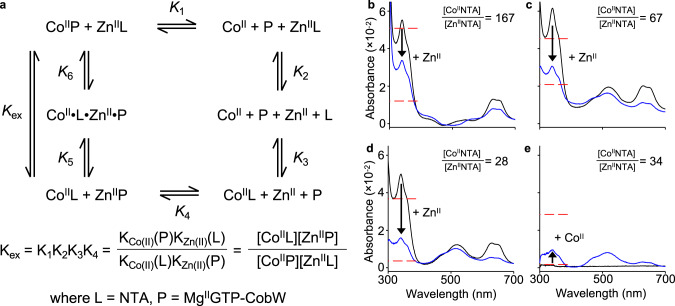
Mg^II^GTP-CobW binds Zn^II^ with sub-picomolar affinity. **a** Representation of the equilibrium for exchange of Co^II^ and Zn^II^ between ligand (L = NTA) and protein (P = Mg^II^GTP-CobW). **b**–**e** Absorbance (relative to metal-free solution) of solutions of CobW (17.9–20.4 µM), Mg^II^ (2.7 mM), GTP (200 µM) and NTA (0.4–4.0 mM) upon (**b**–**d**) first addition of Co^II^ (black trace) then Zn^II^ (blue trace) or (**e**) the reverse, at equilibrium (*n* = 1 for each panel). The absorbance peak at 339 nm corresponds to Co^II^-bound protein. An excess of ligand NTA was used to buffer both metals in each experiment: varying the ratios of ligand-bound metal ions ([Co^II^NTA]/[Zn^II^NTA] = 28–167) shifted the ratios of Co^II^- and Zn^II^-bound protein as predicted by the equilibrium exchange constant in **a**. Consistent *K*_Zn(II)_ values for Mg^II^GTP-CobW were generated at all tested conditions (Supplementary Table 4). Dashed red lines show expected *A*_339 nm_ peak intensities for *K*_Zn(II)_ of Mg^II^GTP-CobW tenfold tighter or weaker than calculated values.

Because of the limiting affinity of quin-2, we employed inter-metal competition, which presumably also occurs within the buffered intracellular milieu, to determine *K*_Zn(II)_ for Mg^II^GTP-CobW. *K*_Zn(II)_ was determined, relative to the known *K*_Co(II)_, via competition between the two metals. This approach required an excess of metal ions competing for a limited number of protein metal-sites (i.e. [Co^II^]_tot_ + [Zn^II^]_tot_ > [CobW]_tot_), thus it was essential to include a buffering ligand, in this case NTA, to control the speciation of all Co^II^ and Zn^II^ in solution (i.e. [NTA]_tot_ > [Co^II^]_tot_ + [Zn^II^]_tot_). The measured equilibrium (*K*_ex_ in Fig. 4a) was the exchange constant for Co^II^/Zn^II^ exchange between the protein (Mg^II^GTP-CobW) and buffering ligand (NTA). Equilibrium ratios of [Co^II^Mg^II^GTP-CobW]/[Zn^II^Mg^II^GTP-CobW] were determined (Fig. 4b–e and Supplementary Table 4): absorbance intensity at A_339 nm_ reported specifically on the Co^II^–protein complex and all remaining protein was Zn^II^-bound (since Mg^II^GTP-CobW was metal-saturated under experimental conditions; Supplementary Fig. 13). The concentrations of NTA-bound metals were determined from mass balance relationships (Eqs. (6–8) in “Methods”). Experiments were conducted at multiple relative availabilities of Co^II^ and Zn^II^ and reciprocally (Fig. 4b–e), with consistent results (Supplementary Table 4), to confirm reliability of measured affinities. We thus determined a tight *K*_Zn(II)_ of 1.9 (±0.6) × 10^−13^ M for Mg^II^GTP-CobW (Supplementary Table 2).

### GTP not GDP enables Co^II^ acquisition by CobW in cells

**Fig. 5:**
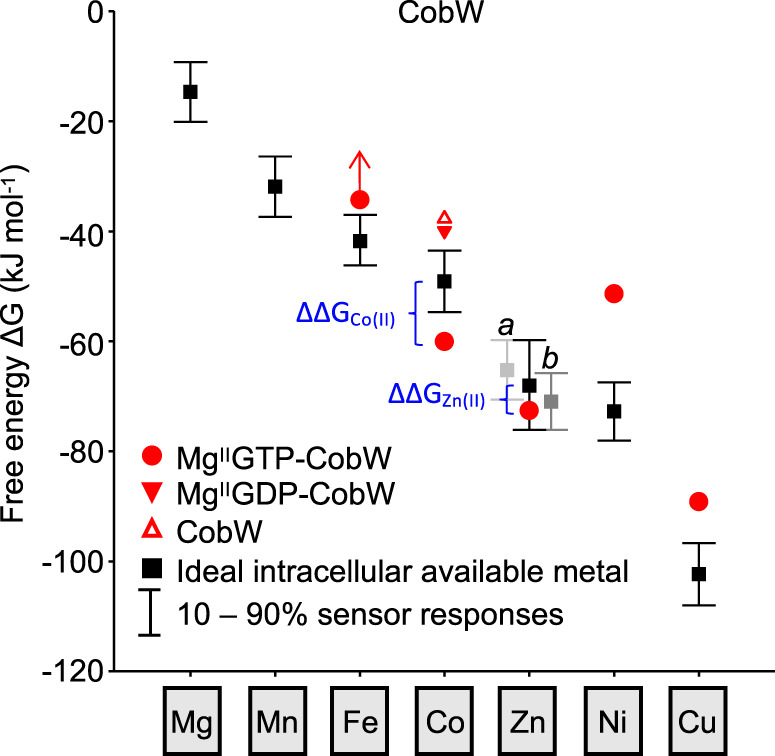
Mg^II^GTP-CobW is predicted to acquire Co^II^ or Zn^II^ in a bacterial cell. Free-energy change (Δ*G*) for metal binding to Mg^II^GTP-CobW (red circles) plotted against the intracellular available free energies for metal binding in a reference bacterial cytosol (values correspond to *Salmonella*) under idealised conditions (i.e. where each metal sensor undergoes half of its transcriptional response; black squares). Intracellular available Δ*G*_Zn(II)_ is the mean of the values determined from the two Zn^II^ sensors ZntR (*a*) and Zur (*b*). Bars show the change in intracellular available Δ*G* as cognate sensors shifts from 10–90% of their responses. Free energy differences (ΔΔ*G*) which favour acquisition of metals by Mg^II^GTP-CobW in vivo are indicated in blue. Δ*G* values for Co^II^ complexes of CobW alone (open red triangle) and Mg^II^GDP-CobW (closed red triangle) are also shown. For Fe^II^ binding to Mg^II^GTP-CobW, arrow indicates limiting Δ*G* > −34.2 kJ mol^−1^.

In the same manner that Fig. 3 considered competition between a ligand (Tar, Bca or quin-2) and a protein (Mg^II^GTP-CobW) for metal binding in vitro, metal acquisition by proteins in vivo likewise involves competition with a surplus of cytosolic ligands that buffer metals to different availabilities^8,9,14,41,42^. Recent work has estimated the buffered availabilities of metals M (where M = Mg^II^, Mn^II^, Fe^II^, Co^II^, Ni^II^, Cu^I^, Zn^II^) in a reference bacterium (*Salmonella*^9^) expressed here as free energies (Δ*G*; Fig. 5). The intracellular available Δ*G* for each metal, Δ*G*_M_, is defined as the free energy required for a ligand to become 50% metalated from available and exchangeable intracellular metal (see Supplementary Note 1). Figure 5 and Supplementary Fig. 14 show the intracellular available Δ*G*_M_ values in an idealised cell (i.e. neither metal deficiency nor excess) defined as the metal availabilities at which the cognate sensors undergoes half of their transcriptional responses. Bars show the changes in available intracellular Δ*G*_M_ as sensors shift from 10–90% (Fig. 5) or 1–99% (Supplementary Fig. 14) of their dynamic ranges. The percentage occupancy of a protein, P, with metal, M, in vivo is governed by the difference between the free energy for protein metalation, Δ*G*_MP_, and the intracellular available Δ*G*_M_ (Eq. (1)) and can be calculated via Eq. (2) (see Supplementary Note 1):

1 $${\Delta}{\Delta}G_{\mathrm{M}} = {\Delta}G_{{\mathrm{MP}}} - {\Delta}G_{\mathrm{M}}$$

2 $${\mathrm{Fractional}}\;{\mathrm{occupancy}}\;({\mathrm{\% }}) = 100 \times \frac{{\left[ {{\mathrm{MP}}} \right]}}{{\left[ {\mathrm{P}} \right]_{{\mathrm{tot}}}}} = 100 \times \frac{{{\mathrm{e}}^{\frac{{ - {\Delta}{\Delta}G_{\mathrm{M}}}}{{RT}}}}}{{1 + {\mathrm{e}}^{\frac{{ - {\Delta}{\Delta}G_{\mathrm{M}}}}{{RT}}}}}$$

In an idealised cell, the Δ*G*_Co(II)_ for CobW and Mg^II^GDP-CobW were both significantly more positive than intracellular available Δ*G*_Co(II)_ (ΔΔ*G*_Co(II)_ ≫ 0; Fig. 5) resulting in negligible Co^II^ occupancies of 1.0% and 2.5% for these two protein forms, respectively. Conversely, Δ*G*_Co(II)_ for Mg^II^GTP-CobW was significantly more negative than intracellular available Δ*G*_Co(II)_ (ΔΔ*G*_Co(II)_ ≪ 0), resulting in almost complete protein metalation (99%). Thus, CobW needs Mg^II^GTP to acquire Co^II^ in a cell.

### Mg^II^GTP-CobW may also acquire Zn^II^

In addition to Co^II^, other metals also bound to Mg^II^GTP-CobW (Figs. 3 and 4). However, ΔΔ*G* for Fe^II^, Ni^II^ and Cu^I^ was significantly greater than zero (Eq. (1) and Fig. 5), thus preventing acquisition of these metals (Eq. (2) and Table 1). In contrast, ΔΔ*G*_Zn(II)_ was <0 with in vivo Zn^II^ occupancy predicted to be 86% (Fig. 5 and Table 1). However, based on Eq. (2), the sum of metal occupancies of Mg^II^GTP-CobW gave an impossible total metalation >100% (Table 1). Since ΔΔ*G* was <0 for both Co^II^ and Zn^II^, a more sophisticated approach needs to account for competition between multiple buffered metals in order to predict how much Zn^II^ binds Mg^II^GTP-CobW in vivo.

**Table 1:** Calculated metal occupancies of COG0523 proteins in an idealised cell^a^.

Metal	Mg^II^GTP-CobW Eq. 2^b^	Mg^II^GTP-CobW Eq. 4^c^	Mg^II^GTP-YeiR Eq. 4^c^	Mg^II^GTPγS-YjiA Eq. 4^c^
Mn^II d^			<0.8%	<1.8%
Fe^II d^	<4.6%	<0.1%	<3.0%	< 3.4%
Co^II^	98.8%	91.9%	10.3%	2.0%
Zn^II^	86.2%	6.9%	24.4%	22.6%
Ni^II^	0.1%	0.0%	0.0%	0.0%
Cu^I^	0.5%	0.0%	0.2%	0.1%
Total	190.3%	98.9%	38.6%	29.8%

^a^Based on metal availabilities in *Salmonella* under idealised conditions (ref. ^9^).

^b^Does not account for competition between different metals for the same high-affinity site in Mg^II^GTP-CobW.

^c^Takes into account competition between multiple intracellular metals for the same site in each protein.

^d^Where only limiting metal–protein affinities (strongest *K*_D_ limit) were determined, calculated occupancy corresponds to a maximum value (denoted by <).

### Calculating inter-metal competition in a cell

Figure 4 considered competition between Co^II^ and Zn^II^ for a single metal-binding site in a protein (Mg^II^GTP-CobW) when the metals were buffered to different availabilities in vitro by an excess of NTA. This can be represented as an available Δ*G*_M_ (Supplementary Table 4). The observed Co^II^ occupancy was a function of the protein’s affinities for both Co^II^ and Zn^II^ relative to their buffered availabilities in solution (i.e. ΔΔ*G* values), as described by Eq. (3) (see Supplementary Note 1).

3 $${\mathrm{Fractional}}\;({\mathrm{\% }})\;{\mathrm{Co}}^{{\mathrm{II}}}\;{\mathrm{occupancy}} = 100 \times \frac{{e^{\frac{{ - {\Delta}{\Delta}G_{{\mathrm{Co}}({\mathrm{II}})}}}{{RT}}}}}{{1 + e^{\frac{{ - {\Delta}{\Delta}G_{{\mathrm{Co}}({\mathrm{II}})}}}{{RT}}} + e^{\frac{{ - {\Delta}{\Delta}G_{{\mathrm{Zn}}({\mathrm{II}})}}}{{RT}}}}}$$

By analogy, in a cytoplasm multiple metals, each buffered to different intracellular available Δ*G*_M_, compete for a single protein-binding site. We generalised Eq. (3) to account for *n* different metals (Eq. (4) and Supplementary Note 1).

4 $${\mathrm{Fractional}}\;(\% )\;{\mathrm{occupancy}}\;\left( {{\mathrm{with}}\;{\mathrm{metal}}\;{\mathrm{M}}_1\;{\mathrm{of}}\;{\mathrm{interest}}} \right) = 100 \times \frac{{e^{\frac{{ - {\Delta}{\Delta}G_{{\mathrm{M}}1}}}{{RT}}}}}{{1 + \mathop {\sum }\nolimits_{k = 1}^{k = n} e^{\frac{{ - {\Delta}{\Delta}G_{{\mathrm{M}}k}}}{{RT}}}}}$$

Thus, we developed a metalation calculator (based on *Salmonella*, Supplementary Data 1) for determining in vivo metal occupancies of proteins, accounting for multiple inter-metal competitions plus competition from components of the intracellular milieu.

### Mg^II^GTP-CobW selects Co^II^ in idealised (*Salmonella*) cells

Since ΔΔ*G* was <0 for binding of both Co^II^ and Zn^II^ to Mg^II^GTP-CobW (Fig. 5), Eq. (4) was next used to predict in vivo metalation in an idealised cell. Between the five metals considered (Fe^II^, Co^II^, Ni^II^, Cu^I^ and Zn^II^), Mg^II^GTP-CobW will favour Co^II^ binding in a cell and calculations via Eq. (4) predicted occupancies of 92% and 7%, for Co^II^ and Zn^II^, respectively (Table 1). Thus, although Mg^II^GTP-CobW affinities for both Co^II^ and Zn^II^ are tight enough to extract either metal from the cytosolic buffer, Co^II^ will outcompete Zn^II^, rationalising specificity but only in an intracellular context where there is competition from other cellular components.

### Related GE3 GTPase YeiR prefers Zn^II^ in idealised *Salmonella*

To test the calculator on a second protein, YeiR from *Salmonella* was overexpressed and purified (Supplementary Fig. 15) in order to determine metal affinities. In view of similarity between YeiR, ZigA^22,23^ and ZagA^24^, notably a deduced binding site for Zn^II^-sensor Zur in the *yeiR* promoter (Supplementary Fig. 16), occupancy with Zn^II^ might be predicted. Perhaps unexpectedly, Mg^II^GTP-YeiR showed a similar (slightly weaker) affinity for Zn^II^ relative to Mg^II^GTP-CobW, the greatest difference in affinity was for Co^II^ (Supplementary Figs. 17–21 and Supplementary Tables 5, 6).

**Fig. 6:**
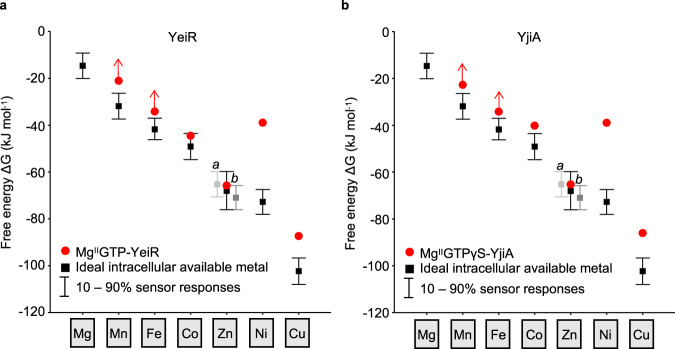
Mg^II^GTP-YeiR and Mg^II^GTP-YjiA preferentially acquire Zn^II^. **a** Free-energy change (Δ*G*) for metal binding to Mg^II^GTP-YeiR (red circles) plotted against the intracellular available free energies for metal binding (as described in Fig. 5; black squares and bars). **b** As **a** for Mg^II^GTPγS-YjiA (red circles). Arrows indicate where only a limiting Δ*G* was determined (thus Δ*G* > plotted value).

Mn^II^ failed to migrate through a gel filtration column with Mg^II^GTP-YeiR even when the running buffer was supplemented with an additional 20 μM MnCl_2_, revealing a Mn^II^ affinity >2 × 10^−4^ M (Supplementary Figs. 17a and 18). Mg^II^GTP-YeiR did not compete with Tar under conditions that imply Fe^II^ affinity ≥1 × 10^−6^ M (Supplementary Fig. 17b). The Co^II^ and Ni^II^ affinities of Mg^II^GTP-YeiR were determined by competition with fura-2 and Mf2, respectively (Supplementary Fig. 17c, d and Supplementary Fig. 19a–d). Data were fit to a 1:1 metal-binding model giving a Co^II^ affinity of 1.5 (±0.7) × 10^−8^ M and Ni^II^ affinity of 1.5 (±0.6) × 10^−7^ M. Competition for Cu^I^ between Mg^II^GTP-YeiR and Bca identified a Cu^I^ affinity of 4.9 (±5.1) × 10^−16^ M (Supplementary Fig. 17e). Competition with quin-2 was used to determine Zn^II^ affinities of Mg^II^GTP-YeiR of 3.0 (±1.2) × 10^−12^ M, and 4.1 (±2.7) × 10^−12^ M for Mg^II^GTPγS-YeiR (Supplementary Figs. 17f, 19e–g, 20d and 21h, i). Equation (4) was used to predict in vivo metalation of Mg^II^GTP-YeiR in an idealised cell. Zn^II^ binding is favoured with calculated occupancies of 24% and 10%, for Zn^II^ and Co^II^ respectively, when sensors are at the mid-points of their dynamic ranges (Fig. 6a and Table 1), and trace amounts of zinc were detected after extensive purification (Supplementary Figs. 22 and 23).

### Related GE3 GTPase YjiA prefers Zn^II^ in idealised *Salmonella*

Co^II^, Ni^II^ and Zn^II^ have all been shown to bind recombinant YjiA in vitro^21^, and the *yjiA* promoter contains no deduced recognition sequence for Zur. To test the calculator on a third COG0523 protein, YjiA was overexpressed, purified (Supplementary Fig. 15), and its affinities for metals determined (Supplementary Figs. 24 and 25, Supplementary Tables 5 and 7).

Affinities were determined for Mg^II^GTPγS-YjiA to avoid nucleotide hydrolysis: this is supported by similar Zn^II^ affinities being measured for Mg^II^GTP-YeiR and Mg^II^GTPγS-YeiR (Supplementary Table 5). Mf2 fully outcompeted Mg^II^GTPγS-YjiA for Mn^II^ and simulations indicate a dissociation constant ≥1 × 10^−4^ M (Supplementary Figs. 24a and 25a–c). Mg^II^GTPγS-YjiA did not compete with Tar under conditions that imply Fe^II^ affinity ≥1 × 10^−6^ M (Supplementary Figs. 24b and 25d–f). The Co^II^, Ni^II^ and Cu^I^ affinities of Mg^II^GTPγS-YjiA were determined using fura-2, Mf2 and Bca, respectively, as described for YeiR, giving a Co^II^ affinity of 9.1 (±2.0) × 10^−8^ M, Ni^II^ affinity of 1.5 (±0.3) × 10^−7^ M and Cu^I^ affinity of 7.6 (±1.4) × 10^−16^ M (Supplementary Figs. 24c–e and 25g–l). Competition with quin-2 was used to determine a Zn^II^ affinity for Mg^II^GTPγS-YjiA of 3.7 (±1.1) × 10^−12^ M, and this was repeated with Mg^II^GTP-YjiA generating a near identical value of 3.3 (±2.5) × 10^−12^ M (Supplementary Figs. 24f and 25m–p). Equation (4) was used to predict in vivo metalation of Mg^II^GTP-YjiA in an idealised cell. Zn^II^ binding is favoured with occupancies of 23% and 2.0% for Zn^II^ and Co^II^, respectively (Fig. 6b and Table 1). Notably, the two Zn^II^ sensors show a relatively wide dynamic range for Δ*G*_Zn(II)_, suggesting that Zn^II^ occupancy could increase dependent upon media [Zn^II^].

### Mg^II^GTP-CobW outcompetes Mg^II^GTP-YeiR for Co^II^

**Fig. 7:**
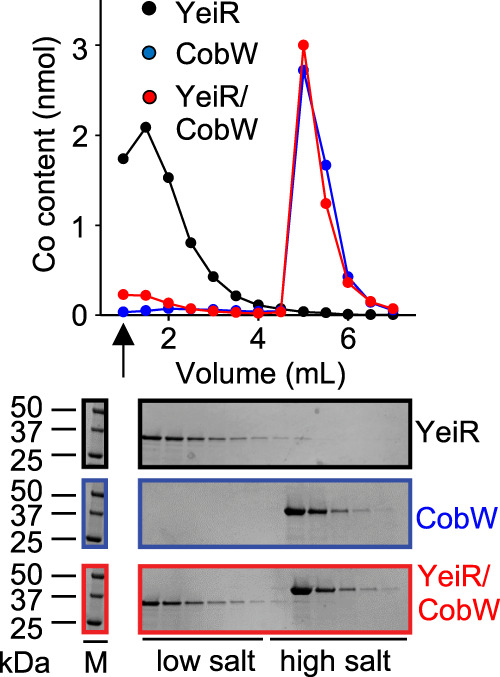
Mg^II^GTP-CobW outcompetes Mg^II^GTP-YeiR for Co^II^. Elution profile of YeiR (10 μM), CobW (10 μM) or both proteins following incubation with GTP (100 μM), Mg^II^ (2.7 mM) and Co^II^ (8 μM) resolved by differential elution from an anion exchange column. Fractions were analysed for Co^II^ by ICP-MS and protein by SDS-PAGE (YeiR alone black, CobW alone blue, both proteins red; *n* = 1). Arrow denotes flow through fractions. Full gel images and SDS-PAGE analysis of flow through fractions shown in Supplementary Fig. 26.

Counterintuitively, Mg^II^GTP-YeiR and Mg^II^GTP-YjiA were predicted to preferentially bind Zn^II^ in vivo, not due to tighter affinities for Zn^II^, but rather due to their weaker Co^II^ affinities relative to Mg^II^GTP-CobW (Table 1, Figs. 5 and 6, and Supplementary Tables 2 and 5). To test relative Co^II^ affinities, Mg^II^GTP-YeiR was competed against Mg^II^GTP-CobW (Fig. 7). YeiR and CobW were incubated with Co^II^ in the presence of Mg^II^ and GTP, then separated by anion exchange chromatography. The chromatography was also conducted with each protein separately. Individually each protein eluted bound to Co^II^, but in competition Co^II^ eluted almost exclusively with Mg^II^GTP-CobW (Fig. 7 and Supplementary Fig. 26), confirming its tighter affinity for Co^II^.

### Fine tuning ∆*G* for metalation in a cell

**Fig. 8:**
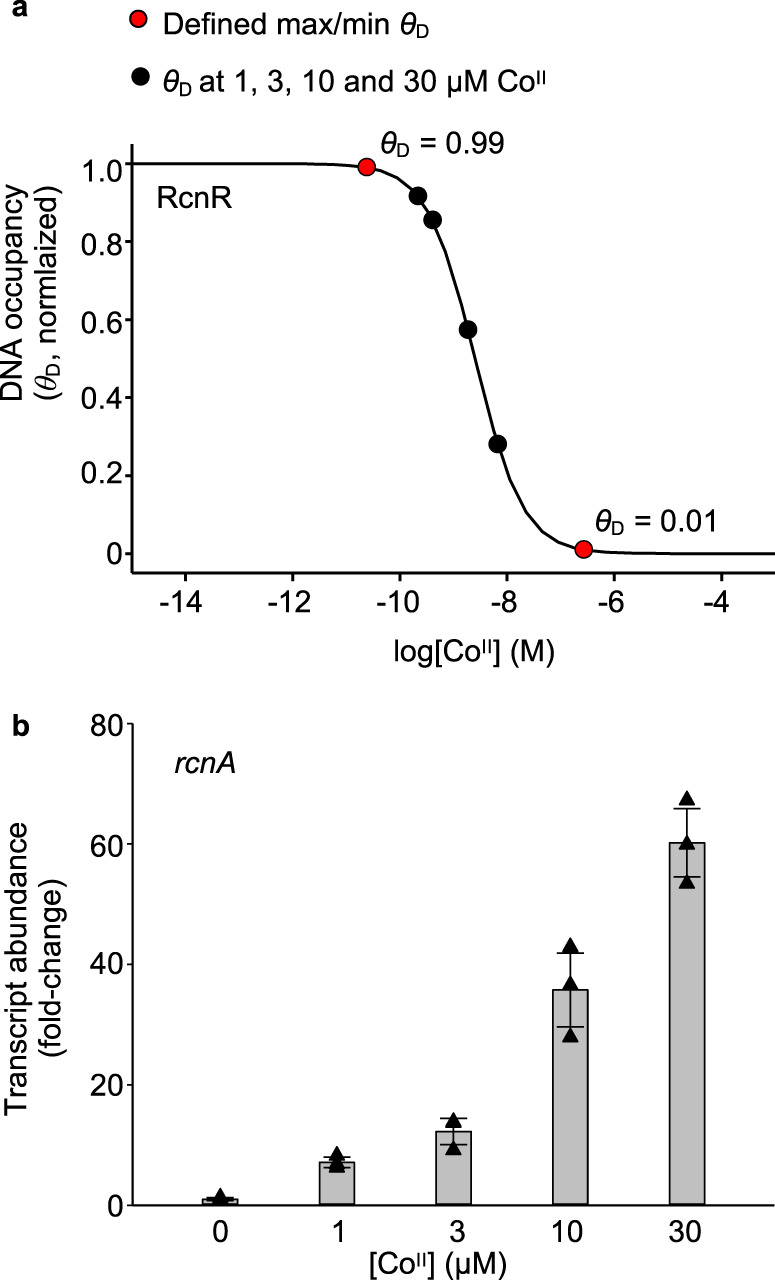
Calculations of conditional Co^II^ availabilities in B_12_-producing E. coli*. **a** Calculated relationship between intracellular Co^II^ availability and normalised DNA occupancy (*θ*_D_) by RcnR. *θ*_D_ of 0 and 1 are the maximum and minimum calculated DNA occupancies. The dynamic range (within which RcnR responds to changing intracellular Co^II^ availability) has been defined as *θ*_D_ of 0.01–0.99 (i.e. 1–99% of RcnR response). The calibrated maximum and minimum fold changes in *rcnA* transcript abundance (i.e. boundary conditions, see Supplementary Fig. 27) therefore correspond to *θ*_D_ of 0.01 and 0.99 in these calculations (red circles). *θ*_D_ for each growth condition (black circles) was calculated from the qPCR response in **b**, assuming a linear relationship between change in *θ*_D_ and change in transcript abundance (Eq. (10)). Corresponding Co^II^ availabilities are listed in Supplementary Table 8. **b** Transcript abundance (relative to untreated control) of the RcnR-regulated gene *rcnA* following 1 h exposure of E. coli* to increasing [Co^II^], measured by qPCR. Data are the mean ± SD of *n* = 3 biologically independent replicates. Triangle shapes represent individual experiments (some data points overlap, experimental values are available in Source Data files).

Calculated free energies for intracellular metalation (Δ*G*_M_) in Figs. 5 and 6 are based on an assumption that cellular metal availabilities are fixed at ideal buffered concentrations where every metal sensor undergoes half of its transcriptional response (i.e. normalised fractional DNA occupancy *θ*_D_ = 0.5, see ref. ^9^). In reality, cellular metal availabilities, and consequently *θ*_D_ of sensors, fluctuate conditionally (e.g. in response to addition of metals or chelators to the growth media). For example, the dynamic response range (defined as *θ*_D_ = 0.99–0.01) of RcnR, the Co^II^ sensor from *Salmonella*, coincides with an increase in the intracellular available [Co^II^] from 2.4 × 10^−11^ to 2.7 × 10^−7^ M, corresponding to an increase in intracellular available Δ*G*_Co(II)_ from −60.6 to −37.5 kJ mol^−1^ (Fig. 8a and Supplementary Table 8).

In order to account for this variation, we developed a method to fine-tune free energy calculations under bespoke culture conditions using quantitative polymerase chain reaction (qPCR) analyses of transcripts regulated by metal sensors. Fine-tuning was performed for Co^II^ in E. coli* which has been engineered to synthesise vitamin B_12_ (*E. coli* and *Salmonella* RcnR share 93% sequence identity and equivalent responses to available Co^II^ were assumed). E. coli* cells were cultured in Luria-Bertani (LB) medium with increasing Co^II^ supplementation. Abundance of the RcnR-regulated *rcnA* transcript (Fig. 8b) was used to calculate *θ*_D_ of RcnR for each condition (via Eq. (10) in “Methods”) following calibration of the maximum and minimum responses (defined as *θ*_D_ = 0.99 and 0.01 at low and high [Co^II^], respectively; Supplementary Fig. 27). This enabled the intracellular Co^II^ availabilities, as conditional free energies, to be calculated from the RcnR *θ*_D_ for each condition (Fig. 8a and Supplementary Table 8).

### Co^II^-acquisition by Mg^II^GTP-CobW predicts B_12_ synthesis

**Fig. 9:**
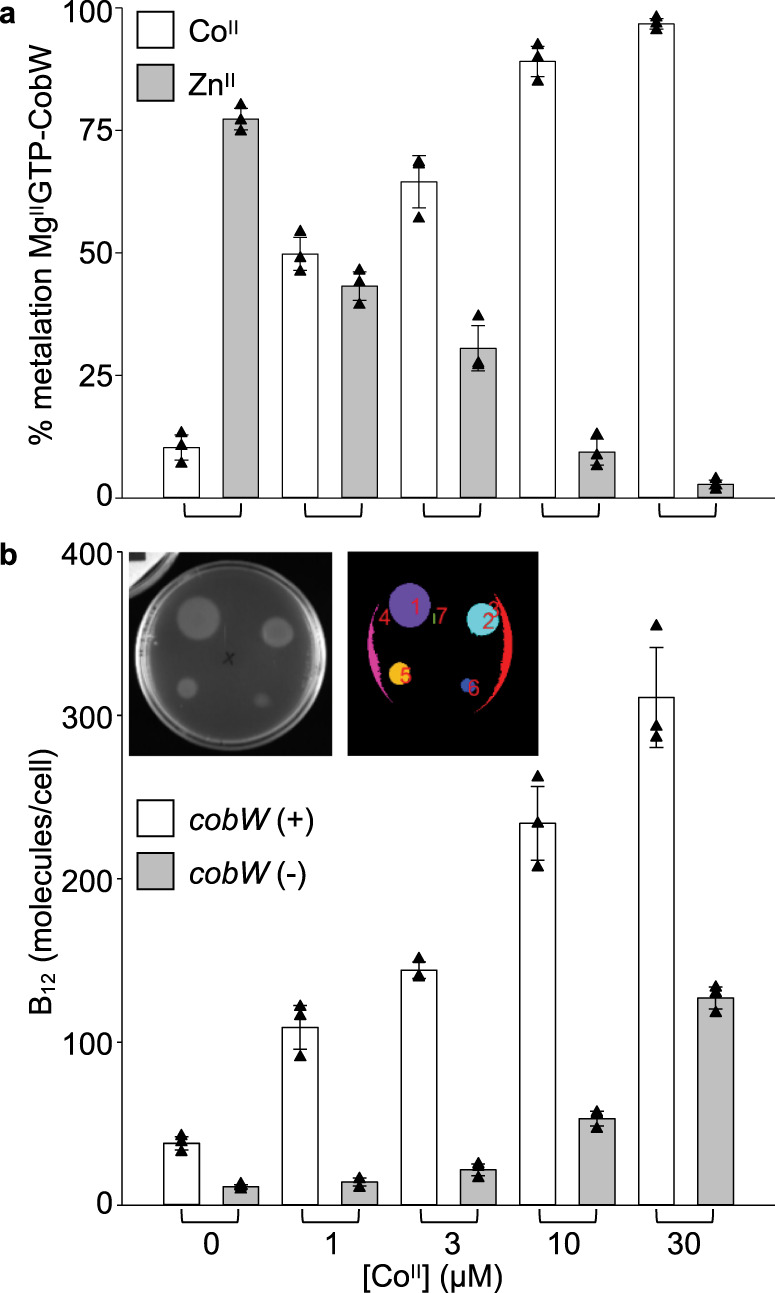
B_12_ production follows predicted metalation of Mg^II^GTP-CobW. **a** Predicted metalation of Mg^II^GTP-CobW with Co^II^ and Zn^II^ (open and grey bars, respectively) in samples treated with defined media [Co^II^]. Intracellular Δ*G*_Co(II)_ for each condition was calculated from *rcnA* expression (Fig. 8 and Supplementary Table 8). **b** B_12_ produced by E. coli* strains with and without *cobW* (open and grey bars, respectively) following 4 h exposure to defined [Co^II^]. B_12_ was detected using a *Salmonella* AR2680 bioassay^65^ (detects corrins, expected to be predominantly B_12_; see “Methods”) and quantified by automated analysis of growth areas (Supplementary Fig. 29 and Supplementary Software 1). Inset shows original image and detected areas (each false coloured) for representative (*n* = 3) bioassay plate of B_12_ calibration standards. All data are the mean ± SD of *n* = 3 biologically independent replicates (with errors in **a** propagated from qPCR data in Fig. 8b). Triangles represent individual experiments (some data points overlap, experimental values are available in Source Data files).

Does the amount of Co^II^ inserted into B_12_ follow the predicted metalation of Mg^II^GTP-CobW? Metal occupancies of Mg^II^GTP-CobW in E. coli* samples were recalculated (via Eq. (4)) using bespoke intracellular available free energies, Δ*G*_Co(II)_, for each growth condition (Fig. 8 and Supplementary Table 8). This predicted that in unsupplemented LB media the protein would be predominantly Zn^II^-bound (10% Co^II^ and 77% Zn^II^) and that Co^II^ occupancies would increase from 10% to 97% as added [Co^II^] increased from 0 to 30 µM (Fig. 9a). Since intracellular Zn^II^ availability was also significant in our predictions, we confirmed that our previous estimation of Δ*G*_Zn(II)_ was valid in LB media (Supplementary Fig. 28). Corrin concentrations (presumed to be predominantly B_12_, noting that intermediates after Co^II^ insertion may also be detected, and that Zn^II^ may competitively inhibit the chelatase complex but not insert into ring-contracted corrins^36^) were measured in E. coli* strains containing or missing *cobW* (Fig. 9b and Supplementary Fig. 29), under the growth conditions for which intracellular available Δ*G*_Co(II)_ was defined (Supplementary Table 8). As the added [Co^II^] increased so did B_12_ production in *cobW(+)*, consistent with the predicted loading of Mg^II^GTP-CobW with Co^II^ (Fig. 9). At higher [Co^II^], CobW-independent B_12_ synthesis became evident. As anticipated, total cellular cobalt increases with supplementation, and the amount of cobalt in B_12_ is <10% of the total cellular cobalt (Supplementary Table 9). The number of additional atoms accumulated per cell exceeds the amount predicted if Co^II^ were not buffered, noting that the internal buffered concentration at 10 μM exogenous Co^II^ is 1.9 nM (Fig. 8 and Supplementary Table 8), and that only 1 atom per cell volume (approximately 1 femtolitre) equates to 1.7 nM. Most importantly, B_12_ synthesis which is dependent on CobW (Fig. 9b, compare *cobW*(+) with *cobW*(−)) matches the trend in predicted metalation of Mg^II^GTP-CobW (Fig. 9a).

## Discussion

Here we relate metal affinities of three putative metallochaperones to a thermodynamic framework, identifying their cognate metals which align with previous speculations^16,20,25^ (Figs. 5, 6 and Table 1). We establish the connection between CobW and Co^II^ and show how CobW can acquire Co^II^ in a cell (Figs. 1–5 and Table 1). Free-energy calculations reveal that in an idealised cell Co^II^ ions will not flow from the cellular milieu to nucleotide-free CobW (ΔΔ*G*_Co(II)_ > 0). Crucially, Co^II^ will flow from the cellular milieu to the Mg^II^GTP form of CobW (ΔΔ*G*_Co(II)_ < 0) (Fig. 5, Supplementary Fig. 30a, Table 1 and Supplementary Table 2). Thus, CobW must first bind Mg^II^GTP in order to acquire Co^II^ inside a cell. In contrast, the product of GTP hydrolysis, Mg^II^GDP-CobW, will release Co^II^ to the cellular milieu (ΔΔ*G*_Co(II)_ > 0) (Fig. 5, Supplementary Fig. 30b and Supplementary Table 2). Thus, the GTPase activity of CobW will facilitate Co^II^ release, for example to CobNST for insertion into the corrin ring of B_12_ (Fig. 2f, g and Supplementary Fig. 6). We establish that CobW enhances B_12_ production when Co^II^ is limiting (Fig. 9b), and Supplementary Fig. 30 illustrates the proposed mechanism. Zn^II^ is identified as the preferred metal for nucleotide-bound forms of YeiR and YjiA: this is due to their weaker affinities for Co^II^ relative to Mg^II^GTP-CobW (Fig. 7 and Supplementary Table 5), rather than tighter affinities for Zn^II^. These data illustrate the value of using the metalation calculator provided as Supplementary Data 1, which can now be broadly applied to metal-speciation in the context of intracellular competition.

Mg^II^GTP-CobW binds Zn^II^ and Cu^I^ more tightly than Co^II^ (Figs. 2e,  3,  4 and Supplementary Table 2), and likewise nucleotide-bound forms of YeiR and YjiA bind Cu^I^ more tightly than Zn^II^. Notably, by taking into account intracellular metal availability, ΔΔ*G* for Cu^I^ was shown to be greater than zero for all three proteins in idealised cells, and also in conditional cells at either 90% or 99% of the dynamic range of the Cu^I^ sensor CueR (Figs. 5, 6 and Supplementary Fig. 14). Thus, these proteins will not acquire Cu^I^. However, for Mg^II^GTP-CobW ΔΔ*G* for Zn^II^ was below zero in an idealised cell suggesting mis-metalation with Zn^II^ (Fig. 5). Indeed, given that CobW binds Zn^II^ more tightly than many known Zn^II^ proteins^14,43^, and comparably to YeiR and YjiA (Supplementary Tables 2 and 5), it seems remarkable that Zn^II^ is not the cognate metal. The data in Fig. 4, plus Supplementary Table 4, illustrate how occupancies of Mg^II^GTP-CobW with Co^II^ versus Zn^II^ change as a function of relative buffered metal availabilities. By reference to intracellular available free energies, the metal with the most negative ΔΔ*G* will have the highest occupancy in vivo (Eq. (4)). In an idealised cell, ΔΔ*G* for Co^II^ is more negative than ΔΔ*G* for Zn^II^ and so the weaker binding metal dominates (Fig. 5 and Supplementary Table 2). In contrast, for nucleotide-bound forms of YeiR and YjiA ΔΔ*G* for Zn^II^ is more negative than ΔΔ*G* for Co^II^ making these deduced Zn^II^ proteins. The previously intractable challenge to understand inter-metal competition in a cell now becomes tractable.

Initial calculations here, and in previous work^9^, assume an idealised cell in which the metal sensors are at the mid-points of their dynamic ranges (*θ*_D_ = 0.5). Therefore, we have calculated the available Δ*G*_Co(II)_ in real (conditional) cells from the responses of RcnR (*θ*_D_) estimated experimentally by qPCR of *rcnA* (Fig. 8 and Supplementary Fig. 27). As with other metallochaperones^10,44^, CobW is crucial when the cognate metal is limiting but at elevated Co^II^, CobW-independent synthesis of B_12_ occurs (Fig. 9b). CobNST must acquire Co^II^ directly from the cytosol at the higher available Δ*G*_Co(II)_. Importantly, CobW-dependent B_12_ synthesis tracked with the calculated Co^II^ occupancy of Mg^II^GTP-CobW in cells supplemented with different amounts of Co^II^ (Fig. 9). By monitoring the responses of sensors for different metals, it will be possible to define available Δ*G*, and predict protein occupancies with diverse metals, in different growth conditions. The calculator should be most accurate in *Salmonella* and closely related species such as *E. coli*. However, metal availabilities can also be adjusted (and/or simulated) to account for species differences, noting that the dynamic ranges of available Δ*G* values might be similar even when total cellular metal changes greatly between species.

Spectral features indicate that the Co^II^ site in Mg^II^GTP-CobW involves thiols, likely derived from the CxCC motif in the GTPase domain, and a tetrahedral geometry (Figs. 1, 2 and Supplementary Fig. 3). However, all COG0523 proteins contain the CxCC motif^16^ and there is now a quest to understand why Co^II^ affinities are weaker for Mg^II^GTP-bound YeiR and YjiA (and, hypothetically, ZigA and ZagA), which bind predominantly to Zn^II^ as a result (Fig. 6). Notably a further pair of conserved cysteine residues (C_56_, C_61_) in CobWs are absent from the homologues (Supplementary Fig. 31). Intriguingly, Ni^II^ binding to Mg^II^GTP-CobW, Mg^II^GTP-YeiR and Mg^II^GTPγS-YjiA does not follow the order of stabilities of metal binding predicted by the Irving-Williams series (Figs. 5 and 6). An appealing explanation is that the allosteric coupling of GTP- and metal-binding imposes a (tetrahedral) geometry on the metal site that would disfavour Ni^II^ coordination (the Irving-Williams series applies where there is no steric selection): notably, related G3E GTPases involved in Ni^II^ homoeostasis (HypB and UreG) display square planar Ni^II^ coordination geometry^45,46^.

The metalation calculator has identified cognate metals for three members of the COG0523 sub-family of GE3 GTPases (Figs. 5, 6 and Table 1), and this work establishes vitamin B_12_ as the ultimate Co^II^-client of CobW (Fig. 9). For YeiR and YjiA, there is now a quest to identify their distinct roles and potential Zn^II^-requiring client(s). Under-metalation of Mg^II^GTP-CobW with Co^II^ (and resultant mis-metalation with Zn^II^, Fig. 9a) could be especially problematic in E. coli* due to the lack of a dedicated Co^II^ import system in this bacterium^47^. This suggests tantalising opportunities to engineer strains suited to the manufacture of vitamin B_12_, either via enhanced Co^II^ uptake through engineered Co^II^-import, or via impaired Zn^II^ accumulation by endogenous Zn^II^-transport systems. By analogy, with almost a half of enzymes requiring metals, an ability to calculate metalation in vivo should have broad applicability in optimising (or subverting) metalation in biotechnology. The calculator (Supplementary Data 1) can be widely used to understand metalation and mismetalation of proteins that acquire Mg^II^, Mn^II^, Fe^II^, Co^II^, Ni^II^, Cu^I^ or Zn^II^ from the milieu inside living cells.

## Methods

### Protein expression and purification

The DNA sequence coding CobW was amplified by PCR using primers 1 and 2 (Supplementary Table 10) with genomic DNA from *R. capsulatus* SB1003 as template. The amplified fragment contained an NdeI restriction site at the 5ʹ end and a SpeI site at the 3ʹ end, allowing it to be cloned into a modified pET-3a vector^34^. The DNA coding sequence of *yeiR* (SL1344_2189) from *S. enterica* serovar Typhimurium strain SL1344 was obtained as a synthetic gene from Eurofins in a pEX plasmid with the T7 promoter and terminator from pET29a flanking *yeiR*. Additionally, the start codon of the gene (GTG) was changed to the more common ATG (pEX*yeiR*). The native coding sequence of *yjiA* (SL1344_4461) was obtained in the same manner (pEX*yjiA*). The correct sequence of each gene (*cobW*, *yeiR* and *yjiA*) was confirmed by DNA sequencing (DBS Genomics – Durham University).

*E. coli* BL21(DE3) pLysS, transformed with either pET3a-*cobW*, pEX-*yeiR* or pEX-*yjiA* were cultured in LB medium with antibiotics carbenicillin (50–100 mg L^−1^) and chloramphenicol (30–34 mg L^−1^). At mid-log phase, protein expression was induced by addition of 0.4 mM (CobW), 0.5 mM (YjiA) or 1.0 mM (YeiR) IPTG. Cells were cultured (with shaking) for 3–4 h at 37 °C (CobW) or at 20 °C overnight (YeiR and YjiA). Cells were harvested and stored at −20 °C prior to use.

Cells overexpressing CobW were resuspended in 20 mM sodium phosphate pH 7.4, 500 mM NaCl, 5 mM imidazole, 5 mM DTT and 1 mM PMSF for lysis (sonication) and cell debris was pelleted by centrifugation (38,000 × *g*, 45 min, 4 °C). Lysate was loaded to a 5-mL HisTrap HP column (GE Heathcare) pre-equilibrated in suspension buffer. CobW binds to the HisTrap column courtesy of a natural His-rich region within the protein. The column was washed with suspension buffer (10 column volumes), then eluted with suspension buffer containing 100 mM imidazole. Protein-containing fractions were incubated with excess (≥10-fold) EDTA for ≥1 h before being loaded to a HiLoad 26/600 Superdex 75 size exclusion column equilibrated in 50 mM Tris pH 8.0, 150 mM NaCl, 5 mM DTT and eluted in the same buffer. Peak CobW-containing fractions (determined from SDS-PAGE) were pooled, concentrated to ~0.5 mL (using a Vivaspin® 15 Turbo centrifugal concentrator). Protein identity was confirmed using ESI-MS by Durham University Department of Chemistry Mass Spectrometry Service. ESI-MS data were recorded on a QtoF Premier mass spectrometer coupled to an Acuity UPLC system (Waters). Protein samples were desalted prior to injection using a Waters MassPrep desalting cartridge (2.1 × 10 mm) and eluted with a linear acetonitrile gradient (20–80% v/v; 0.1% formic acid). Spectra were processed using Masslynx 4.1, deconvoluted using MaxEnt 1 and data imported into SigmaPlot software for preparation of figures.

Cells overexpressing YeiR were resuspended in 20 mM sodium phosphate pH 7.4, 100 mM NaCl, 5 mM DTT, 1 mM PMSF for lysis (sonication) and cell debris was pelleted by centrifugation (31,191 × *g*, 15 min, 8 °C). Soluble lysate was applied to a 5-mL HisTrap column (GE Healthcare) equilibrated with lysis buffer without PMSF. The column was washed with equilibration buffer before elution with equilibration buffer containing 10, 50 and 100 mM imidazole. YeiR eluted in the buffer containing 50 mM imidazole. EDTA was added to the YeiR-containing fraction to a final concentration of 10 mM and stored overnight at 4 °C. The sample was applied to HiLoad 26/600 Superdex 75 (GE Healthcare) equilibrated with 50 mM Tris pH 8, 150 mM NaCl, 5 mM DTT and eluted with the same buffer. Peak fractions were pooled and applied to a 5-mL Q anion exchange column (GE Healthcare) equilibrated with the size exclusion column buffer. Column flow through and wash were collected before eluting the column with size exclusion column buffer with the addition of 1 M NaCl. YeiR displays no affinity for the Q column and elutes in the flow through and wash. The remaining major contaminant elutes with 1 M NaCl. The flow through and wash from the Q column were pooled and concentrated using a Spin-X UF concentrator (Corning, 10 kDa molecular weight cut-off).

Cells overexpressing YjiA were resuspended in 20 mM Tris 7.5, 100 mM NaCl, 5 mM DTT, 1 mM PMSF for lysis (sonication) and cell debris was pelleted by centrifugation (two consecutive 20 min runs, 39,191 × *g*, 4 °C) before passing clarified supernatant through a 20-µm nylon membrane filter. Soluble lysate was applied to a 5-mL HisTrap column (GE Healthcare) equilibrated with lysis buffer without PMSF, collecting the flow through and a one column volume wash. This step removes a major contaminant which binds to the HisTrap column. Pooled flow through and wash fractions were applied to a 5-mL HiTrap Q-Sepharose fast flow column (GE Healthcare) equilibrated with lysis buffer without PMSF. The column was washed with equilibration buffer then equilibration buffer with 200 mM NaCl before elution of YjiA by application of a 50-mL gradient of 200–600 mM NaCl in equilibration buffer collecting 5 mL fractions. YjiA typically eluted between 10 and 30 mL. Fractions containing the highest concentration of YjiA with the lowest degree of contamination, as judged by SDS-PAGE, were stored overnight (4 °C) with EDTA to a final concentration of 5 mM. Fractions were concentrated to 5 mL using a Spin-X UF concentrator (Corning, 10 kDa molecular weight cut-off) and applied to HiLoad 26/600 Superdex 75 (GE Healthcare) equilibrated with lysis buffer without PMSF and eluted with the same buffer. Fractions were pooled and concentrated to 5–20 mg mL^−1^ using a Spin-X UF concentrator (Corning, 10 kDa molecular weight cut-off) before storage at −80 °C.

Following purification, CobW, YeiR and YjiA samples were transferred to an anaerobic glovebox (Belle Technology), (0.5–1 mL) applied to a PD-10 Desalting Column prepacked with Sephadex G-25 medium (GE Healthcare) equilibrated with chelex-treated and N_2_-purged buffer (10 mM HEPES pH 7.0, 100 mM NaCl, 400 mM KCl) and eluted in the same buffer. Proteins were quantified by *A*_280 nm_ using experimentally determined extinction coefficients (*ε* = 15,300 M^−1^ cm^−1^ for CobW, 52,745 M^−1^ cm^−1^ for YeiR, and 27,900 M^−1^ cm^−1^ for YjiA) determined by quantitative amino acid analysis (Alta Bioscience Ltd). Samples were confirmed to be of high purity by SDS-PAGE (full gel images are available in the Supplementary Information), and ≥92.5% (YeiR) and ≥95% (CobW, YjiA) metal-free (by inductively coupled plasma-mass spectrometry; ICP-MS). ICP-MS of one sample of YjiA was performed 3 weeks after buffer exchange and >5% zinc detected, but results were consistent with replicates containing <5% zinc. ICP-MS was conducted using Durham University Bio-ICP-MS Facility (PlasmaLab software; Thermo Fisher). Reduced thiol content was determined by reaction with ~10-fold excess of Ellman’s reagent 5,5ʹ-dithio-bis-[2-nitrobenzoic acid] (DTNB; produces one equivalent of chromophore TNB^2−^ per protein thiol, *A*_412 nm_ = 14,150 M^−1^ cm^−1^)^48,49^. For CobW >5.5 cysteines were found to be reactive with DTNB (expected value = 6), for YeiR 4–5 cysteines (expected value = 5), and for YjiA >4.5 cysteines (expected value = 5).

### Preparation of metal stocks

All metal stocks were prepared in ultrapure water from appropriate salts (MgCl_2_, (NH_4_)_2_Fe(SO_4_)_2_, CoCl_2_, NiSO_4_, NiCl_2_, CuSO_4,_ ZnCl_2_, ZnSO_4_) and quantified by ICP-MS analysis. Fe^II^ stocks were prepared by dissolving (NH_4_)_2_Fe(SO_4_)_2_·6H_2_O in deoxygenated 0.1% (v/v) HCl in an anaerobic chamber. Reaction with excess ferrozine (Fz; ∼50-fold) confirmed that iron was ≥95% reduced (Fe^II^Fz_3_ *ε*_562 nm_ = 27,900 M^−1^ cm^−1^)^50^. Concentrated stocks were diluted daily in deoxygenated ultrapure water to prepare working solutions and confirmed to be ≥90% Fe^II^. Other metal stocks were prepared aerobically as concentrated stocks and diluted to working solutions with deoxygenated ultrapure water in an anaerobic chamber. Cu(I) was generated in situ (from CuSO_4_) by hydroxylamine (1–10 mM) which quantitatively reduces Cu(II) to Cu(I) in the presence of excess chelator (L = Bca or Bcs) to form Cu^I^L_2_ complexes^51^.

### Determination of CobW Co^II^-binding stoichiometries

Metal-binding experiments were conducted in an anaerobic chamber in deoxygenated, chelex-treated 10 mM HEPES pH 7.0, 100 mM NaCl, 400 mM KCl. For stoichiometry determinations, Co^II^ was titrated into a solution of CobW (15–30 µM) together with relevant nucleotides (supplied in ∼10-fold excess of protein concentration for GTP and GDP and ∼3-fold excess for GMPPNP and GTPγS, as specified in figure legends) in the absence or presence of Mg^II^ (2.7 mM). Absorbance was recorded using a Lambda 35 UV-visible spectrophotometer (Perkin Elmer; UV-Win lab software). The extinction coefficient of Co^II^Mg^II^GTP-CobW (*ε*_339 nm_ = 2800 ± 100 M^−1^ cm^−1^, average ± s.d of *n* = 3 independent titrations) was determined from absorbance at saturating metal concentrations (Supplementary Fig. 3g). Extinction coefficients of related complexes Co^II^Mg^II^GMPPNP-CobW, Co^II^Mg^II^GTPγS-CobW, Co^II^_2_GTP-CobW, Co^II^_2_GMPPNP-CobW and Co^II^_2_GTPγS-CobW were similarly determined (Figs. 1, 2 and Supplementary Figs. 1, 3): within experimental error, all produced the same extinction coefficient as for Co^II^Mg^II^GTP-CobW, thus *ε*_339 nm_ = 2800 M^−1^ cm^−1^ was assigned to all species.

Gel-filtration chromatography experiments were performed by incubating CobW (10 µM) and Co^II^ (30 µM) for 30 min with or without cofactor GMPPNP (30 µM) then applying 0.5 mL to a PD-10 Sephadex G-25 gel-filtration column (GE Healthcare). Eluted fractions (0.5 mL) were analysed for cobalt by ICP-MS and for protein by Bradford assay.

### Determination of CobW metal affinities via ligand competition

Ligand competition experiments were conducted in an anaerobic chamber in deoxygenated, chelex-treated 10 mM HEPES pH 7.0, 100 mM NaCl, 400 mM KCl, except where high concentrations (≥1 mM) of competing ligand were employed, where 50 mM HEPES was used to maintain buffered pH 7.0. Absorbance was recorded using a Lambda 35 UV-visible spectrophotometer (Perkin Elmer). Fluorescence spectra were recorded using a Cary Eclipse fluorescence spectrophotometer (Agilent; Cary Eclipse scan application software). Affinities were determined at a range of different competing conditions (by varying the competing ligand and/or the protein:ligand ratio) to ensure reliability: details are documented in Supplementary Table 1. Scripts used for data fitting (using Dynafit^52^) are provided in Supplementary Software 2. The effect of Mg^II^ (2.7 mM) on apparent dissociation constants of ligand standards (EGTA, NTA, fura-2, Mf2 and quin-2) was calculated to be insignificant under the conditions of competition experiments (Supplementary Table 3). For probes with undefined Mg^II^ affinities (Tar, Bca), control experiments confirmed that addition of Mg^II^ (2.7 mM) had negligible effect on competition experiments (Supplementary Figs. 10d and 12). Thus, Mg^II^ was not incorporated into the curve-fitting models.

For determination of weaker (*K*_D_ > 10 nM) Co^II^ binding affinities (CobW and Mg^II^GDP -CobW), CoCl_2_ was titrated into a solution of 5-Oxazolecarboxylic acid, 2-(6-(bis(carboxymethyl)amino)-5-(2-(2-(bis(carboxymethyl)amino)-5-methylphenoxy)ethoxy)-2-benzofuranyl)-pentapotassium salt (fura-2; quantified by *ε*_363 nm_ = 28,000 M^−1^ cm^−1^)^53^ and CobW in the presence or absence of cofactors (MgCl_2_ and GDP) and fluorescence emission (*λ*_ex_ = 360 nm; *λ*_max_ ~505 nm) was recorded at equilibrium. Co^II^-dependent fluorescence quenching of fura-2 was used to determine Co^II^ speciation. For determination of Co^II^ binding affinities tighter than 10 nM (Mg^II^GMPPNP-CobW, Mg^II^GTPγS-CobW and Mg^II^GTP-CobW), CoCl_2_ was titrated into a solution containing CobW, competing ligand (EGTA or NTA), MgCl_2_ and nucleotide (GMPPNP, GTPγS or GTP). UV-visible absorbance (relative to metal-free solution) was recorded at equilibrium to determine Co^II^ speciation (*ε*_339 nm_ = 2800 M^−1^ cm^−1^ for Co^II^-bound proteins). Data were fit using Dyanfit^52^ to models describing 1:1 binding stoichiometry for Co^II^:protein and 1:1 binding stoichiometry for Co^II^:ligand (ligand = fura-2, EGTA or NTA). Ligand dissociation constants at pH 7.0: fura-2 *K*_Co(II)_ = 8.6 × 10^−9^ M (ref. ^39^); EGTA *K*_Co(II)_ = 7.9 × 10^−9^ M (ref. ^38^); NTA *K*_Co(II)_ = 2.2 × 10^−8^ M (ref. ^38^).

(NH_4_)Fe(SO_4_)_2_ was titrated into a solution of Tar (16 µM), MgCl_2_ (2.7 mM) and GTP (500 µM) in the absence or presence of CobW (50 µM) and UV-visible absorbance recorded at equilibrium to define Fe^II^ speciation (Fe^II^Tar_2_ *ε*_720_ =  19,560 M^−1^ cm^−1^ under experimental conditions, Supplementary Fig. 8a). Data were fit in Dynafit^52^ to a model describing 1:1 binding stoichiometry for Fe^II^:protein and 1:2 binding stoichiometry for Fe^II^:Tar using *β*_2,Fe(II)_ = 4.0 × 10^13^ M^−2^ for Tar at pH 7.0 (ref. ^54^). Experimental data were compared to simulated fits with defined protein *K*_Fe(II)_ = 10^−6^ M, 10^−7^ M, allowing limiting *K*_D_ ≥ 10^−6^ M for Mg^II^GTP-CobW to be determined. Tar stock concentrations were quantified using *ε*_470 nm_ = 24,800 M^−1^ cm^−1^ (reported value at pH 7.0 (ref. ^54^)) and verified by titration with metal stocks (Fe^II^ or Ni^II^, quantified by ICP-MS).

NiSO_4_ was titrated into a solution of Tar (20 µM), CobW (10–30 µM), MgCl_2_ (2.7 mM) and GTP (100–300 µM) and UV-visible absorbance recorded at equilibrium to determine Ni^II^ speciation (Ni^II^Tar_2_ Δ*ε*_535 nm_ = 3.8 (±0.1) × 10^4^ M^−1^ cm^−1^ relative to ligand only solution; Supplementary Fig. 10a). Tar stock concentrations were quantified as above. Data were fit using Dynafit^52^ to a model describing 1:1 stoichiometry Ni^II^:protein and 1:2 stoichiometry Ni^II^:Tar; *β*_2,Ni(II)_ = 4.3 (±0.6) × 10^15^ M^−2^ for Tar at pH 7.0 was independently determined by preparing a series of solutions of NiTar_2_ ([Ni^II^] = 15 µM, [Tar] = 36 µM) with varying EGTA concentrations (0–400 µM) and measuring UV-visible absorbance at equilibrium (following 1–2 h incubation). EGTA *K*_Ni(II)_ = 5.0 × 10^−10^ M at pH 7.0 (ref. ^38^). Data were fit to Eq. (5)^51^ using Kaleidagraph (Synergy Software).

5 $$\frac{{[{\mathrm{EGTA}}]_{{\mathrm{tot}}}}}{{[{\mathrm{Ni}}^{{\mathrm{II}}}]_{{\mathrm{tot}}}}} = 1 - \frac{{[{\mathrm{Ni}}^{{\mathrm{II}}}{\mathrm{Tar}}_2]}}{{\left[ {{\mathrm{Ni}}^{{\mathrm{II}}}} \right]_{{\mathrm{tot}}}}} + K_{\mathrm{D}}\left( {{\mathrm{EGTA}}} \right){\beta}_2({\mathrm{Tar}})\left( {\frac{{[{\mathrm{Tar}}]_{{\mathrm{tot}}}}}{{[{\mathrm{Ni}}^{{\mathrm{II}}}{\mathrm{Tar}}_2]}} - 2} \right)^2[{\mathrm{Ni}}^{{\mathrm{II}}}{\mathrm{Tar}}_2]\left( {1 - \frac{{[{\mathrm{Ni}}^{{\mathrm{II}}}{\mathrm{Tar}}_2]}}{{\left[ {{\mathrm{Ni}}^{{\mathrm{II}}}} \right]_{{\mathrm{tot}}}}}} \right)$$

CuSO_4_ was titrated into a solution of Bca (1.0 mM), CobW (10–30 µM), MgCl_2_ (2.7 mM), GTP (100–300 µM) and reductant NH_2_OH (1.0 mM) which quantitatively reduces Cu^II^ to Cu^I^ in the presence of a strong Cu^I^ ligand (e.g. Bca: *β*_2,Cu(I)_ = 1.6 × 10^17^ M^−2^ (ref. ^38^)). UV-visible absorbance was recorded at equilibrium to define Cu^I^ speciation (Cu^I^Bca_2_ *ε*_562_ = 7900 M^−1^ cm^−1^ (ref. ^38^)) and data were fit using Dynafit^52^ to a model describing 1:1 stoichiometry Cu^I^:protein and 1:2 stoichiometry Cu^I^:Bca.

ZnCl_2_ was titrated into a solution containing quin-2 (10 µM), CobW (10 µM), MgCl_2_ (2.7 mM) and GTP (50 µM) and UV-visible absorbance recorded at equilibrium. Quin-2 was quantified using *ε*_261 nm_ = 37,000 M^−1^ cm^−1^ (ref. ^55^). *K*_Zn(II)_ for Mg^II^GTP-CobW was beyond the range of this experiment (significantly tighter than quin-2) and only a limiting affinity was determined (*K*_Zn(II)_ < 10^−12^ M).

### Zn^II^ affinity of Mg^II^GTP-CobW via inter-metal competition

Solutions containing CobW (17.9–20.4 µM), MgCl_2_ (2.7 mM), GTP (200 µM) and ligand NTA (0.4–4.0 mM) were titrated with CoCl_2_ (0.3–3.0 mM) and ZnCl_2_ (15.3–25.5 µM) and UV-visible absorbance was recorded at equilibrium to determine Co^II^ occupancy of CobW (*ε*_339 nm_ = 2800 M^−1^ cm^−1^ for Co^II^Mg^II^GTP-CobW). Details of individual experiments are in Supplementary Table 4. The total concentration of Co^II^ and Zn^II^ in each solution was limiting, such that both metals were buffered by ligand NTA. Metal speciation was determined from the mass balance relationships given in Eqs. (6–8) (cofactors Mg^II^GTP omitted for clarity). Thus, *K*_Zn(II)_ for Mg^II^GTP-CobW was calculated from the exchange equilibria (*K*_ex_) in Fig. 4a, relative to known *K*_Co(II)_ for the protein (Supplementary Table 2) and ligand dissociation constants (NTA *K*_Zn(II)_ = 1.18 × 10^−8^ M, *K*_Co(II)_ = 2.24 × 10^−8^ M (ref. ^38^)). These calculations are valid given that [M]_free_ ≪ [M]_tot_ (M = Co^II^ or Zn^II^, buffered by excess NTA), the concentration of non-metalated protein is negligible (Supplementary Fig. 13) and potential ternary complexes involving metal, protein and NTA are transient species only with insignificant concentration at thermodynamic equilibrium (varying ratios of buffered metals, [Co^II^NTA]/[Zn^II^NTA], were used to confirm consistency of *K*_D_ values at multiple equilibria; see Fig. 4 and Supplementary Table 4).

6 $$[{\mathrm{Co}}^{{\mathrm{II}}}{\mathrm{NTA}}] = [{\mathrm{Co}}^{{\mathrm{II}}}]_{{\mathrm{tot}}} - [{\mathrm{Co}}^{{\mathrm{II}}}{\mathrm{CobW}}]$$

7 $$[{\mathrm{Zn}}^{{\mathrm{II}}}{\mathrm{CobW}}] = [{\mathrm{CobW}}]_{{\mathrm{tot}}} - [{\mathrm{Co}}^{{\mathrm{II}}}{\mathrm{CobW}}]$$

8 $$[{\mathrm{Zn}}^{{\mathrm{II}}}{\mathrm{NTA}}] = [{\mathrm{Zn}}^{{\mathrm{II}}}]_{{\mathrm{tot}}} - [{\mathrm{Zn}}^{{\mathrm{II}}}{\mathrm{CobW}}]$$

### Determination of YeiR and YjiA metal stoichiometries and affinities

Investigation of protein–metal interactions and competition experiments to determine metal affinities were performed in 10 mM HEPES pH 7, 100 mM NaCl, 400 mM KCl (chelex treated and N_2_ purged) with the inclusion of nucleotides and MgCl_2_ as noted in figure legends. Absorbance was recorded using a Lambda 35 UV-visible spectrophotometer (Perkin Elmer). Fluorescence spectra were recorded using a Cary Eclipse fluorescence spectrophotometer (Agilent). Scripts used for data fitting (using Dynafit^52^) are provided in Supplementary Software 2.

CoCl_2_ was titrated into a solution of fura-2 (*ε*_363 nm_ = 28,000 M^−1^ cm^−1^, *K*_Co(II)_ = 8.6 × 10^−9^ M (refs. ^39,53^)) in the presence of YeiR or YjiA and fluorescence emission (510 nm) recorded at equilibrium (*λ*_ex_ = 360 nm, 20 °C). Data were fit to a model describing 1:1 Co^II^:fura-2 and 1:1 Co^II^:protein binding stoichiometries using Dynafit^52^.

NiCl_2_ was titrated into a solution of Mf2 (*ε*_369 nm_ = 22,000 M^−1^ cm^−1^, *K*_Ni(II)_ = 5 × 10^−8^ M (refs. ^53,56^)) in the presence of YeiR or YjiA and the absorbance (323–325 and 365–366 nm) recorded at equilibrium. Data were fit (both wavelengths simultaneously) to a model describing 1:1 Ni^II^:Mf2 and 1:1 Ni^II^:protein binding stoichiometries using Dynafit^52^.

ZnSO_4_ was titrated into a solution of Mf2 (*K*_Zn(II)_ = 2 × 10^−8^ M (ref. ^57^)), PAR (*β*_2 Zn(II)_ = 2 × 10^12^ M^−2^ (ref. ^58^)) or quin-2 (*K*_Zn(II)_ = 3.7 × 10^−12^ M (ref. ^55^)) in the presence of YeiR or YjiA and the absorbance (325 and 366 nm Mf2; 500 nm PAR; 265 or 269 nm quin-2) recorded at equilibrium. Concentrations of PAR and quin-2 stocks were determined by direct titration with ZnSO_4_. Data were fit to a model describing 1:1 Zn^II^:quin-2 and 1:1 Zn^II^:YjiA binding stoichiometries. Zn:YeiR stoichiometries were fit as 1:1, or allowed to be determined in fitting as described in the text using Dynafit^52^.

CuSO_4_ was titrated into a solution of Bca (Cu^I^Bca_2_ ε_562 nm_ = 7900 M^−1^ cm^−1^, *β*_2 Cu(I)_ = 10^17.2^ M^−2^ (ref. ^59^)) in the presence and absence of YeiR or YjiA (with inclusion of hydroxylamine) and absorbance (562 nm) recorded at equilibrium. Protein Cu^I^ affinity was calculated using Eq. (9), for the tightest binding event. Calculated affinities were simulated using Dynafit^52^, and overlaid on the data.

9 $$K_{\mathrm{D}}\beta _2 = \frac{{\left( {\frac{{[{\mathrm{P}}]_{{\mathrm{tot}}}}}{{[{\mathrm{MP}}]}}} \right) - 1}}{{\left( {\left( {\frac{{[{\mathrm{L}}]_{{\mathrm{tot}}}}}{{[{\mathrm{ML}}_2]}} - 2} \right)^2[{\mathrm{ML}}_2]} \right)}}$$

(NH_4_)Fe(SO_4_)_2_ was titrated into a solution of Tar (Tar_2_Fe(II) *ε*_720 nm_ =  19,000 M^−1^ cm^−1^, *β*_2 Fe(II)_ = 10^13.6^ M^−2^ (at pH 7.0) (ref. ^54^) in the presence and absence of YeiR or YjiA and absorbance (720 nm) recorded at equilibrium. Data were fit to a model describing 1:2 Fe^II^:Tar and 1:1 Fe^II^:protein binding stoichiometries using Dynafit^52^.

MnCl_2_ was titrated into a solution of Mf2 (*K*_Mn(II)_ = 6.1 × 10^−6^ M (ref. ^9^)) in the presence of YjiA and the absorbance (330 and 365 nm) recorded at equilibrium. Data were fit (both wavelengths simultaneously) to a model describing 1:1 Mn^II^:Mf2 and 1:1 Mn^II^:protein binding stoichiometries using Dynafit^52^.

Gel filtration chromatography of YeiR was performed by application of 0.5 mL (10 μM) to a PD-10 Desalting Column prepacked with Sephadex G-25 medium equilibrated with buffer supplemented with 2.7 mM MgCl_2_ with or without 20 μM MnCl_2_ and eluted with the same buffer. YeiR was incubated with 20 μM MnCl_2_ for 20 min prior to application to the column. Protein content of collected fractions was assayed by *A*_280 nm_ and Bradford assay, metal content by ICP-MS.

### Inter-protein competition for Co(II)

Experiments were performed in an anaerobic glovebox. YeiR (10 μM) was incubated with GTP (100 μM), MgCl_2_ (2.7 mM) and CoCl_2_ (8 μM) in 10 mM HEPES pH 7.0, 40 mM NaCl, 160 mM KCl (chelex treated and N_2_ purged) for 10 min before addition of CobW (10 μM) (total volume upon CobW addition = 1.1 mL). The mixture was incubated for a further 30 min before application of 1 mL of the incubation reaction to a 1-mL Q anion exchange column (GE Healthcare) equilibrated with 10 mM HEPES pH 7.0, 40 mM NaCl, 160 mM KCl (chelex treated and N_2_ purged), collecting the flow through. The column was sequentially eluted with equilibration buffer collecting six 0.5 mL fractions followed by 10 mM HEPES pH 7.0, 200 mM NaCl, 800 mM KCl (chelex treated and N_2_ purged) collecting six 0.5-mL fractions. Fractions were analysed for protein content by SDS-PAGE and for metal content by ICP-MS. Controls were conducted concurrently as above but with YeiR or CobW alone.

### GTPase activity assays

CobW (20–50 µM) was incubated with CoCl_2_ (0.9 equivalents Co^II^:protein) and GTP (200 µM) in an anaerobic chamber in N_2_-purged, chelex-treated 10 mM HEPES pH 7.0, 100 mM NaCl, 400 mM KCl. Aliquots of solution taken at various time intervals (0–390 min) were loaded to a 5-mL HiTrap Q HP column (GE Healthcare) equilibrated in buffer (20 mM HEPES pH 7.0, 100 mM NaCl) and eluted with a linear NaCl gradient (100–500 mM NaCl). Nucleotides were detected by UV absorbance (254 nm or 280 nm) and the ratio of GTP:GDP in each sample was calculated by integration of the respective peak areas.

### Growth of E. coli* strains

E. coli* strains used in this work are derived from *E. coli* MG1655 (DE3) engineered to contain a set of B_12_ biosynthesis genes from *R. capsulatus*^60,61^, and *Brucella melitensis (B. melitensis)*^34^. Strain ED741 (E. coli* without *cobW*) is MG1655 with P_lac_-T7RNAP-P_T7_-cobAIGJFMKLHBROQJD-bluE-C-bluF-PUB-cbiW-VE-P_T7_-cobNST while strain ED732 (E. coli* with *cobW*) is MG1655 with P_lac_-_T7_RNAP-P_T7_-cobAIGJFMKLHBROQJD-bluE-C-bluF-PUB-cbiW-VE-P_T7_-cobWNST. All *R. capsulatus* and *B. melitensis* (*cobG, cobR, cobE*) genes were cloned individually in pET3a and subcloned together using the link and lock method^34^. The synthetic operons were transferred into the *E. coli* genome using CRISPR technology^62^. Although chromosomally integrated B_12_ biosynthesis genes are IPTG-inducible under the control of the T7 promoter, in the current experiments IPTG was not added to cell cultures to avoid potential disruptions of cellular metal homoeostasis caused by over-production of metalloproteins.

All cultures and media were prepared in plasticware or acid-washed glassware to minimise trace metal contamination. LB medium was inoculated with overnight culture of E. coli* (OD_600 nm_ = 0.025) and incubated at 37 °C with shaking until OD_600 nm_ reached ~0.2. Aliquots (5 mL or 50 mL) of this culture were treated with sterile CoCl_2_, H_2_O, EDTA or ZnCl_2_ (100× concentrated stocks) to reach final concentrations as specified in figure legends (Figs. 8b, 9b and Supplementary Figs. 27, 28a, b, d and 29c) and incubated under the same conditions for a further 1–4 h. Samples used for RNA extraction were taken 1 h after treatment. Samples for B_12_ quantification and OD_600 nm_ readings were taken 4 h after treatment to ensure detectable corrinoid production.

### Determination of transcript abundance in E. coli*

Aliquots (1 mL) of E. coli* culture from each growth condition were stabilised in RNAProtect Bacteria Reagent (2 mL; Qiagen) and cells pellets were frozen at −80 °C prior to processing. RNA was extracted using an RNeasy Mini Kit (Qiagen) as described by the manufacturer. RNA was quantified by absorbance at 260 nm and treated with DNAse I (2.5 U/μL; Fermentas). cDNA was generated using the ImProm-II Reverse Transcriptase System (Promega) with 300 ng RNA per reaction, and control reactions without reverse transcriptase were conducted in parallel. Transcript abundance was determined using primers 3 and 4 for *rcnA*, 5 and 6 for *zntA*, 7 and 8 for *znuA*, 9 and 10 for *rpoD*, each pair designed to amplify ~110 bp fragment. Quantitative PCR analysis was carried out in 20 µL reactions using 5 ng of cDNA, 0.8 µM of each appropriate primer and PowerUp SYBR Green Master Mix (Thermo Fisher Scientific). Three technical replicates of each sample (i.e. biological replicate) were analysed using a Rotor-Gene Q 2plex (Qiagen; Rotor-Gene-Q Pure Detection software), plus control reactions without cDNA template for each primer pair. The fold change, relative to the mean of the control condition for each sensor, was calculated using the 2^−ΔΔCT^ method^63^, with *rpoD* as the reference gene. *C*_q_ values were calculated with LinRegPCR after correcting for amplicon efficiency^64^.

### Intracellular available ∆*G*_Co(II)_ under bespoke conditions

Intracellular available ∆*G*_metals_ were first calculated from available metal concentrations where the cognate sensor is at 1%, 10%, 50%, 90% and 99% of its response (i.e., *θ*_D_ = 0.01, 0.1, 0.5, 0.9, 0.99; Supplementary Note 1). Available metal concentrations corresponding to these fractional occupancies were determined using known metal affinities, DNA affinities, protein abundances and numbers of DNA binding sites determined for *Salmonella* sensors^9^, using excel spreadsheet (Supplementary Dataset 1) and MATLAB code (Supplementary Note 3) available in ref. ^9^.

Fractional responses (*θ*_D_) of RcnR at bespoke growth conditions were calculated from transcript abundance of *rcnA* via Eq. (10):

10 $${\mathrm{Conditional}}\;\theta _{\mathrm{D}} = 0.99 - 0.98 \times \left( {\frac{{{\mathrm{fold}} - {\mathrm{change}}_{{\mathrm{obs}}} - 1}}{{{\mathrm{fold}} - {\mathrm{change}}_{{\mathrm{max}}} - 1}}} \right)$$

where fold-change_obs_ is the observed fold-change in *rcnA* transcript abundance at the bespoke condition and fold-change_max_ is the maximum fold-change in *rcnA* transcript abundance at the calibration limit (corresponding to maximum abundance); all fold-changes were determined relative to the defined control condition (untreated LB) corresponding to minimum *rcnA* transcript abundance (see Supplementary Fig. 27c). Equation (10) defines maximum and minimum transcript abundances as corresponding to *θ*_D_ of 0.01 and 0.99, respectively (see Fig. 8a), and assumes a linear relationship between change in *θ*_D_ and change in transcript abundance.

The intracellular available [Co^II^] concentration corresponding to each RcnR *θ*_D_ was calculated using known metal affinity, DNA affinities, protein abundance, number of DNA binding sites determined for *Salmonella* RcnR^9^, to calculate the Co^II^-dependent response of *E. coli* RcnR (93% sequence identity) using excel spreadsheet (Supplementary Dataset 1) and MATLAB code (Supplementary Note 3) available in ref. ^9^. The intracellular available Δ*G*_Co(II)_ for each condition was calculated using Eq. (11), where [Co^II^] is the intracellular available Co^II^ concentration, *R* (gas constant) = 8.314 × 10^−3^ kJ K^−1^ mol^−1^ and *T* (temperature) = 298.15 K (see Supplementary Note 1).

11 $${\mathrm{Intracellular}}\;{\mathrm{available}}\;{\Delta}G_{{\mathrm{Co}}({\mathrm{II}})} = RT\ln \left[ {{\mathrm{Co}}^{{\mathrm{II}}}} \right]$$

### **E**stimation of intracellular available ∆*G*_Zn(II)_ in LB media

Fractional responses (*θ*_D_) of Zur and ZntR in LB media were calculated from transcript abundance of *znuA* and *zntA*, via Eqs. (10) and (12), respectively:

12 $${\mathrm{Conditional}}\;\theta _{\mathrm{D}} = 0.01 + 0.98 \times \left( {\frac{{{\mathrm{fold}} - {\mathrm{change}}_{{\mathrm{obs}}} - 1}}{{{\mathrm{fold}} - {\mathrm{change}}_{{\mathrm{max}}} - 1}}} \right)$$

where fold-change_obs_ is the observed fold-change in transcript abundance in LB and fold-change_max_ is the maximum fold-change in transcript abundance at the calibration limit (corresponding to maximum abundance); all fold-changes were determined relative to defined control conditions corresponding to minimum transcript abundance (see Supplementary Fig 28a, b). Equation (12) defines maximum and minimum transcript abundances as corresponding to *θ*_D_ of 0.99 and 0.01, respectively, and assumes a linear relationship between change in *θ*_D_ and change in transcript abundance.

The intracellular available [Zn^II^] concentration corresponding to each *θ*_D_ was calculated using known metal affinities, DNA affinities, protein abundance, number of DNA binding sites determined for *Salmonella* homologues, to calculate the Zn^II^-dependent responses of *E. coli* ZntR and Zur (both >92% sequence identity to *Salmonella*) using excel spreadsheet (Supplementary Dataset 1) and MATLAB code (Supplementary Note 3) available in ref. ^9^. The intracellular available Δ*G*_Zn(II)_ was calculated using Eq. (13), where [Zn^II^] is the intracellular available Zn^II^ concentration, *R* (gas constant) = 8.314 × 10^−3^ kJ K^−1^ mol^−1^ and *T* (temperature) = 298.15 K (see Supplementary Note 1).

13 $${\mathrm{Intracellular}}\;{\mathrm{available}}\;{\Delta}G_{{\mathrm{Zn}}({\mathrm{II}})} = RT\ln \left[ {{\mathrm{Zn}}^{{\mathrm{II}}}} \right]$$

### Quantification of vitamin B_12_ in E. coli* cultures

Aliquots (20 mL) of E. coli* culture from each growth condition were taken, and cell pellets frozen at −20 °C. To quantify corrin production (assumed to be predominantly B_12_, since E. coli* contains genes for the complete pathway), E. coli* pellets were thawed, resuspended in H_2_O (0.2 mL), boiled for 15 min (95 °C) and centrifuged to remove cell debris. An aliquot (10 µL) of each supernatant was applied to *Salmonella typhimurium* AR2680 (*ΔmetE, ΔcbiB*) pre-innoculated bioassay plates^65^, and incubated at 37 °C overnight. Plates were imaged together with a 1-cm^2^ reference area on black background (see example in Supplementary Data 2) using a Gel-Doc XR + gel documentation system (BioRad; ImageLab software). Images were analysed in MATLAB using the code in Supplementary Software 1 to determine the growth area (in cm^2^) of each sample. A calibration curve relating growth areas to B_12_ concentration was generated using B_12_ standards (cyanocobalamin; 1–100 nM; quantified by *A*_360 nm_ = 27,500 M^−1^ cm^−1^ at pH 10 (ref. ^66^)) in parallel with E. coli* lysates, using the same batch of bioassay plates (Supplementary Fig. 29a, b). To determine the number of cells in each sample, solutions of E. coli* at varying cell densities (OD_600 nm_ = 0.2–0.9) were prepared, serially diluted (2000-fold), and the number of cells per mL quantified using a CASY® cell counter. The resulting correlation factor (4.4 ± 0.1 × 10^8^ cells mL^−1^ OD_600 nm_^−1^) was used to convert OD_600 nm_ to cell number (Supplementary Fig. 29c, d).

### Metal content of E. coli* cells

Aliquots (20 mL) of E. coli* culture from each growth condition were taken and pellets were washed twice with 0.5 M sorbitol, 200 μM EDTA, 20 mM Tris pH 8.5. Cell pellets were suspended in ultrapure 65% (v/v) HNO_3_ (0.4 mL) to digest (>24 h), then diluted tenfold in 2.5% HNO_3_ before metal analysis by ICP-MS.

### Statistics and reproducibility

Sample sizes were chosen based on prior experimental experience, and to give consistent results, following convention in the literature for equivalent analyses. Experiments designed to derive quantitative values used to model or test calculations of metalation were performed in triplicate or more (*n* = 3–5) to enable calculation of SD (listed in Tables or shown as error bars in figures). The number of independent experiments or biologically independent samples is shown in figure legends or footnotes of Tables.

### Reporting summary

Further information on research design is available in the Nature Research Reporting Summary linked to this article.

## Supplementary information

Supplementary Information

Peer Review File

Description of Additional Supplementary Files

Supplementary Data 1

Supplementary Data 2

Supplementary Software 1

Supplementary Software 2

Reporting Summary

## Data Availability

All data are available within the article, its Supplementary Information files, plus PDB entry 1NIJ. Source data are provided with this paper.

## Source data

Source Data

## Code availability

Equation derivations, Excel spreadsheet (with instructions) constituting a metalation calculator, MATLAB code (with instructions) for use in B_12_ assays and Dynafit scripts are available in Supplementary Note 1, Supplementary Data 1, Supplementary Software 1 and Supplementary Software 2, respectively.
