# Supplementary material for: Calculating metalation in cells reveals CobW acquires CoII for vitamin B12 biosynthesis while related proteins prefer ZnII
Source: Nat Commun. 2021 Feb 19;12:1195. doi: 10.1038/s41467-021-21479-8 (PMC7895991; doi:10.1038/s41467-021-21479-8)
Supplement: Supplementary file 1 — Supplementary Information [file 41467_2021_21479_MOESM1_ESM.pdf]

Supplementary information for

Calculating metalation in cells reveals CobW acquires Co^{II} for vitamin B_{12} biosynthesis while related proteins prefer Zn^{II}

Tessa R. Young[1,2]*, Maria Alessandra Martini[1,3], Andrew W. Foster[1,2], Arthur Glasfeld[1,2,4], Deenah Osman[1,2], Richard J. Morton[5], Evelyne Deery[6], Martin J. Warren[6,7], Nigel J. Robinson[1,2]*

[1]Department of Biosciences, Durham University, Durham, UK. [2]Department of Chemistry, Durham University, Durham, UK. [3]Max Planck Institute for Chemical Energy Conversion, Mülheim an der Ruhr, Germany. [4]Chemistry Department, Division of Mathematical and Natural Sciences, Reed College, Oregon, USA. [5]Department of Mathematics, Physics, and Electrical Engineering, Northumbria University, Newcastle-upon-Tyne, UK. [6]School of Biosciences, University of Kent, Canterbury, Kent, UK., [7]Quadram Institute Bioscience, Norwich Research Park, Norfolk, UK.

*e-mail:

tessa.r.young@durham.ac.uk; nigel.robinson@durham.ac.uk

Table of contents

Supplementary Table 1. Conditions of CobW ligand competition experiments

Supplementary Table 2. Metal affinities (and corresponding free energies) of CobW

Supplementary Table 3. Calculated effect of 2.7 mM Mg^{II} on apparent ligand dissociation constants at pH 7.0

Supplementary Table 4. Calculated stability constants (K_D and ΔG values) for Zn^{II}-binding to Mg^{II}GTP-CobW under various conditions, from Zn^{II}-Co^{II} inter-metal competition experiments (Fig. 4)

Supplementary Table 5. Metal affinities (and corresponding free energies) of YeiR and YjiA

Supplementary Table 6. Conditions of YeiR ligand competition experiments

Supplementary Table 7. Conditions of YjiA ligand competition experiments

Supplementary Table 8. Calculations of conditional intracellular available [Co^{II}] (and corresponding $\Delta G_{Co(II)}$) for *E. coli** cultures in Co^{II}-supplemented LB media

Supplementary Table 9. Total metal in *E. coli**

Supplementary Table 10. Oligonucleotides used in this work

Supplementary Fig. 1. Source files and replicate data for Fig. 1.

Supplementary Fig. 2. Visible absorbance features from Fig. 1g

Supplementary Fig. 3. Different GTP analogues assemble similar metal-binding sites in CobW

Supplementary Fig. 4. $K_{Co(II)}$ quantification for CobW, in the absence or presence of nucleotides, using a variety of competing conditions

Supplementary Fig. 5. $K_{Co(II)}$ quantification for Mg^{II}GTP-CobW at varying nucleotide concentrations

Supplementary Fig. 6. CobW-catalysed GTP hydrolysis is also slow at 4:1 ratio GTP:CobW

Supplementary Fig. 7. Mg^{II}GDP-CobW has a similar Co^{II} site to Mg^{II}GTP-CobW but with weaker $K_{Co(II)}$

Supplementary Fig. 8. Further details of $K_{Fe(II)}$ quantification for Mg^{II}GTP-CobW using Tar

Supplementary Fig. 9. Further details of $K_{Ni(II)}$ quantification for Mg^{II}GTP-CobW

Supplementary Fig. 10. Determination of β_2 for $Ni^{II}Tar_2$ at pH 7.0

Supplementary Fig. 11. Further details of $K_{Cu(I)}$ quantification for Mg^{II}GTP-CobW

Supplementary Fig. 12. Effect of Mg^{II} on Cu^{I}-competition experiments using Bca

Supplementary Fig. 13. Mg^{II}GTP-CobW is metal-saturated in Fig. 4 inter-metal competition experiments

Supplementary Fig. 14. The accessible range of intracellular available free energies for metal-binding as sensors shift from 1 – 99% of their responses

Supplementary Fig. 15. SDS-PAGE analysis of purified YeiR and YjiA

Supplementary Fig. 16. Zur recognition sequences in the promoter region of *yeiR*

Supplementary Fig. 17. Metal affinities of Mg^{II}GTP-YeiR

Supplementary Fig. 18. Additional data for Supplementary Fig. 17a

Supplementary Fig. 19. Replicate data for Supplementary Fig. 17c,d and f

Supplementary Fig. 20. Fitting of Zn^{II} binding data for Mg^{II}GTP-YeiR

Supplementary Fig. 21. Additional replicates and fittings for Supplementary Fig. 19e-g and 20b,d

Supplementary Fig. 22. Size exclusion chromatography of YeiR shows co-purification of (trace amounts of) Zn^{II}

Supplementary Fig. 23. Source figures for Supplementary Fig. 22

Supplementary Fig. 24. Metal affinities of Mg^{II}GTPγS-YjiA and Mg^{II}GTP-YjiA

Supplementary Fig. 25. Replicate data for Supplementary Fig. 24

Supplementary Fig. 26. Source figures for Fig. 7

Supplementary Fig. 27. Calibration of maximum and minimum *rcnA* responses

Supplementary Fig. 28. Estimation of Zn^{II} availability within *E. coli** cells in defined growth conditions (conditional cells)

Supplementary Fig. 29. Quantification of vitamin B_{12} in *E. coli**

Supplementary Fig. 30. Proposed mechanism of CobW

Supplementary Fig. 31. Sequence alignment of COG0523 proteins investigated in this work

Supplementary Note 1. Derivation of equations for calculating the *in vivo* metal occupancy of a protein (equations 1-4 in main text).

Supplementary References

Supplementary Table 1. Conditions of CobW ligand competition experiments [a]

Metal (M)	Nucleotide [b]	Competing ligand (L) [c]	$[\text{CobW}]_{tot}$ (μM)	$[L]_{tot}$ (μM)	$[M]_{max}$ (μM)	Species detected (signal) [d]	K_D (M)	Figure ref.
Co^{II}	None	fura-2	34	10	80	Co^{II}fura-2	1.1×10^{-7}	S4f
			37	10	82	($F_{505\ nm}$)	2.7×10^{-7}	2c
			50	10	70		3.9×10^{-7}	S4g
	GDP		10	10	22		8.8×10^{-8}	S4i
			20	8	46		1.0×10^{-7}	2d
			30	8	50		1.1×10^{-7}	S4h
	GMPPNP	EGTA	20	20	40	Co^{II}CobW	2.3×10^{-9}	S4a
			20	40	84	($A_{339\ nm}$)	3.2×10^{-9}	2a
			20	40	90		2.6×10^{-9}	S4b
	GTPγS		20	200	222		1.1×10^{-10}	S4c
			20	1000	40		2.8×10^{-10}	S4d
			20	2000	40		1.2×10^{-10}	S4e
	GTP		20	100	170		nd [e]	S4k
			20	500	122		4.0×10^{-11}	S4l
			18	2000	34		2.7×10^{-11}	2e
		NTA	18	4000	34		2.2×10^{-11}	S4m
Fe^{II}		Tar	50	16	80	$Fe^{II}(Tar)_2$	$< 10^{-6}$ [f]	3a/S8b
			50	16	60	($A_{720\ nm}$)	$< 10^{-6}$ [f]	S8b
			50	16	60		$< 10^{-6}$ [f]	S8b
Ni^{II}			10	20	40	$Ni^{II}(Tar)_2$	3.8×10^{-10}	S9b
			20	20	38	($A_{535\ nm}$)	1.9×10^{-9}	S9c
			30	20	42		6.9×10^{-10}	3b
Cu^{I}		Bca	10	1000	34	$Cu^{I}(Bca)_2$	2.6×10^{-16}	S11c
			20	1000	34	($A_{562\ nm}$)	1.2×10^{-16}	3c
			30	1000	32		3.4×10^{-16}	S11d

[a] All experiments were conducted anaerobically in N_2-purged, chelex-treated HEPES (10 – 50 mM) pH 7.0, 100 mM NaCl, 400 mM KCl

[b] GTP and GDP were supplied in ~10-fold excess of protein concentration and non-hydrolysable analogues (GMPPNP and GTPγS) were supplied in ~3-fold excess of protein concentration (as specified in figure legends). All affinity determinations in presence of nucleotides included 2.7 mM Mg^{II}.

[c] fura-2, EGTA and NTA form 1:1 Co^{II}:ligand complexes: at pH 7.0 fura-2 $K_{Co(II)} = 8.6 \times 10^{-9}$ M (ref.[1]); EGTA $K_{Co(II)} = 7.9 \times 10^{-9}$ M (ref.[2]); and NTA $K_{Co(II)} = 2.2 \times 10^{-8}$ M (ref.[2]). Tar forms 1:2 metal:ligand complexes at pH 7.0: $\beta_{2,Fe(II)} = 4.0 \times 10^{13}\ M^{-2}$ (ref.[3]); $\beta_{2,Ni(II)} = 4.3 \times 10^{15}\ M^{-2}$ (Supplementary Fig. 10). Bca forms a 1:2 Cu^{I}:ligand complex with $\beta_2 = 1.6 \times 10^{17}\ M^{-2}$ (ref.[4])

[d] For cases where titration end-point was reached, probe responses were defined as variable parameters in Dynafit model (final fitted responses were all consistent with known extinction coefficients ± experimental error). For cases where titration end-point was not reached, probe responses were fixed to known extinction coefficients of metal-bound species in Dynafit models: $Co^{II}Mg^{II}$GTP*-CobW (where GTP* = GTP, GTPγS, GMPPNP) $\varepsilon_{339\ nm} = 2{,}800\ M^{-1}\ cm^{-1}$ (Fig. 2 and Supplementary Fig. 3), $Fe^{II}(Tar)_2$ $\varepsilon_{720\ nm} = 19{,}560\ M^{-1}\ cm^{-1}$ (Supplementary Fig. 8a), $Ni^{II}(Tar)_2$ $\Delta\varepsilon_{535\ nm} = 38{,}000\ M^{-1}\ cm^{-1}$ with respect to ligand only (Supplementary Fig. 10a), $Cu^{I}(Bca)_2$ $\varepsilon_{562\ nm} = 7{,}900\ M^{-1}\ cm^{-1}$ (ref.[2]).

[e] Insufficient [EGTA] for effective competition with Mg^{II}GTP-CobW, data not used for K_D determination.

[f] Negligible competition between probe Tar and protein for Fe^{II} binding, only a limiting affinity was determined (n = 3 independent experiments, same conditions).

Supplementary Table 2. Metal affinities (and corresponding free energies) of CobW

Species	Metal	K_D (M) [a]	ΔG_{MP} (kJ mol^{-1})
CobW	Co^{II}	2.5 (±1.1) ×10^{-7}	-37.7 ± 1.2
CobW-Mg^{II}GDP		1.0 (±0.1) ×10^{-7}	-39.9 ± 0.3
CobW-Mg^{II}GMPPNP		2.7 (±0.4) ×10^{-9}	-48.9 ± 0.4
CobW-Mg^{II}GTPγS		1.7 (±0.8) ×10^{-10}	-55.7 ± 1.2
CobW-Mg^{II}GTP		3.0 (±0.8) ×10^{-11}	-60.1 ± 0.7
	Fe^{II}	> 10^{-6} [b]	> -34.2 [b]
	Ni^{II}	9.8 (±6.5) ×10^{-10}	-51.4 ± 1.7
	Cu^{I}	2.4 (±0.9) ×10^{-16} [c]	-89.2 ± 1.0 [c]
	Zn^{II}	1.9 (±0.6) ×10^{-13}	-72.6 ± 0.8

[a]Data are mean ± SD of n = 3 independent experiments conducted at a range of competing conditions (see Supplementary Table 1), except $K_{Zn(II)}$ of CobW-Mg^{II}GTP is mean ± SD of n = 4 independent experiments (see Supplementary Table 4).
[b]Probe Tar outcompetes CobW-Mg^{II}GTP in Fe^{II}-binding experiments (see Fig. 3a), limiting K_D value only.
[c]Assuming only tightest affinity site binds Cu^{I} under experimental conditions (one-site model). A model with two sites of equivalent affinity fits to marginally-weaker K_D values (6.1 (±2.0) ×10^{-16} M).

Supplementary Table 3. Calculated effect of 2.7 mM Mg^{II} on apparent ligand dissociation constants at pH 7.0

Ligand (L)	$K_{Mg(II)}$ (M) [a]	$K_{Mg(II)}$ (M) (pH 7.0) [b]	α coefficient (2.7 mM Mg^{II}, pH 7.0) [c]
EGTA	6.2×10^{-6} [d]	9.7×10^{-2} [e]	0.97
NTA	3.9×10^{-6} [d]	2.1×10^{-3} [e]	0.44
Fura-2		9.8×10^{-3} [f]	0.78
Mf2		2.7×10^{-3} [g]	0.50
quin-2		2.0×10^{-3} [h]	0.43

[a] Absolute dissociation constants (K_D) for $Mg^{II}L$ complexes (0.1 M ionic strength)
[b] Conditional dissociation constants (K_D) for $Mg^{II}L$ complexes at pH 7.0 (0.1 M ionic strength)
[c] $\alpha = 1/(1+[Mg]/K_{Mg(II)})$ describes the effect of 2.7 mM Mg^{II} on the apparent ligand affinity for other metals at pH 7.0: $K_D' = K_D/\alpha$, where K_D is the ligand dissociation constant at pH 7.0 in the absence of Mg^{II} and K_D' is the apparent ligand dissociation constant at pH 7.0 in the presence of 2.7 mM Mg^{II}.
[d] From ref [5]
[e] Calculated from absolute dissociation constant as described in ref [2]
[f] At pH 7.2, from ref [6]
[g] At pH 7.4, from ref [7]
[h] From refs[6,8]

Supplementary Table 4. Calculated stability constants (K_D and ΔG values) for Zn^{II}-binding to Mg^{II}GTP-CobW under various conditions, from Zn^{II}-Co^{II} inter-metal competition experiments (Fig. 4)

$[CobW]_{tot}$ (μM)	$[NTA]_{tot}$ (μM)	$[Co^{II}]_{tot}$ (μM)	$[Zn^{II}]_{tot}$ (μM)	$[Co^{II}NTA]/[Zn^{II}NTA]$ [a]	$[Co^{II}CobW]/[Zn^{II}CobW]$ [a,b]	$\Delta G_{Co(II)}$ [c] ($kJ\ mol^{-1}$)	$\Delta G_{Zn(II)}$ [c] ($kJ\ mol^{-1}$)	CobW $K_{Zn(II)}$ (M) [b]	$\Delta G_{Zn(II)\text{-}CobW}$ ($kJ\ mol^{-1}$) [b]
19.7	4000	3000	25.5	167	1.58	-40.9	-55.2	1.5×10^{-13}	-73.2
20.4	400	300	15.3	67	0.99	-41.2	-53.2	2.4×10^{-13}	-72.0
18.2	3710	302	23.2	28	0.45	-49.7	-59.5	2.6×10^{-13}	-71.8
17.9	3710	302	23.2	34	0.24	-49.7	-60.0	1.1×10^{-13}	-73.9

[a] Calculated ratios at equilibrium

[b] In the presence of excess Mg^{II} (2.7 mM) and GTP (200 μM), omitted for clarity.

[c] *in vitro* available $\Delta G_{Co(II)}$ and $\Delta G_{Zn(II)}$ determined at equilibrium; *ie* $\Delta G_{Co(II)} = RT \ln[Co^{II}] = RT\ K_{Co(II)}(NTA) \times ([Co^{II}\text{-}NTA]/[NTA])$

Supplementary Table 5. Metal affinities (and corresponding free energies) of YeiR and YjiA

Metal	Nucleotide	YeiR K_D (M) [a]	YjiA K_D (M) [a]	YeiR ΔG_{MP} (kJ mol^{-1})	YjiA ΔG_{MP} (kJ mol^{-1})
Mn^{II}	GTP	≥2.0 × 10^{-4}	-	≥-21.1	-
	GTPγS	-	≥10^{-4}	-	≥-22.8
Fe^{II}	GTP	≥ 10^{-6}	-	≥-34.2	-
	GTPγS	-	≥10^{-6}	-	≥-34.2
Co^{II}	GTP	1.5 (±0.7) × 10^{-8}	-	-44.7 ± 1.2	-
	GTPγS	-	9.1 (±2.0) × 10^{-8}	-	-40.2 ± 0.5
Ni^{II}	GTP	1.5 (±0.6) × 10^{-7}	-	-38.9 ± 1.0	-
	GTPγS	-	1.5 (±0.3) × 10^{-7}	-	-38.9 ± 0.5
Zn^{II}	GTP	3.0 (±1.2) × 10^{-12}	3.3 (±2.5) × 10^{-12}	-65.8 ± 0.9	-65.5 ± 2.5
	GTPγS	4.1 (±2.7) × 10^{-12}	3.7 (±1.1) × 10^{-12}	-65.0 ± 1.4	-65.3 ± 0.7
Cu^{I}	GTP	4.9 (±5.1) × 10^{-16}	-	-87.4 ± 2.4	-
	GTPγS	-	7.6 (±1.4) × 10^{-16}	-	-86.3 ± 0.5

[a]Data are mean ± SD of n = 3-5 independent experiments (see Supplementary Tables 6 and 7) except where only limiting value was determined.

Supplementary Table 6. Conditions of YeiR ligand competition experiments [a]

Metal (*M*)	Nucleotide[b]	Competing ligand (*L*) [c]	$[YeiR]_{tot}$ (μM)	$[L]_{tot}$ (μM)	$[M]_{max}$ (μM)	Species detected (signal) [e]	K_D (M)	Figure ref.
Fe^{II}	GTP	Tar	10	15	12.8	$Fe^{II}(Tar)_2$ ($A_{720\ nm}$)	≥1.0 × 10^{-6} [f]	S17b
Co^{II}	GTP	fura-2	8.9	10	38.5	Co^{II}fura-2 ($F_{510\ nm}$)	1.0 × 10^{-8}	S17c
			8.9	11	33.9		2.2 × 10^{-8}	S19a
			10	13	33.0		1.1 × 10^{-8}	S19b
Ni^{II}	GTP	Mf2	8.9	11	38.1	Ni^{II}mf2 ($A_{325\ nm}$ and $A_{366\ nm}$	1.4 × 10^{-7}	S17d
			8.9	11	36.3		9.9 × 10^{-8}	S19c
			10	11	35.4		2.2 × 10^{-7}	S19d
Cu^{I}	GTP	Bca	10	800	5.5	$Cu^{I}(Bca)_2$ ($A_{562\ nm}$)	1.0 × 10^{-16} [g]	S17e
			10	800	5.5		1.1 × 10^{-16} [g]	S17e
			10	800	9.2		1.1 × 10^{-15} [g]	S17e
			10	800	5.5		1.4 × 10^{-16} [g]	S17e
			10	800	5.5		1.0 × 10^{-15} [g]	S17e
Zn^{II}	GTP	quin-2	10 (6.0) [d]	7.7	13.4	Zn^{II}quin-2 ($A_{269\ nm}$)	2.6 × 10^{-12} [h]	S17f
			10 (7.3) [d]	10	16.0		3.6 × 10^{-12} [h]	S19e
			10 (6.0) [d]	8.6	12.5		1.6 × 10^{-12} [h]	S19f
			10 (5.7) [d]	8.3	13.4		4.3 × 10^{-12} [h]	S19g
	GTPγS		10 (9.2) [d]	9.6	13.4		2.7 × 10^{-12} [h]	S20d
			10 (10) [d]	9.2	13.4		7.2 × 10^{-12} [h]	S21h
			10 (9.7) [d]	9.0	13.4		2.3 × 10^{-12} [h]	S21i

[a] All experiments conducted anaerobically in N_2-purged, chelex-treated HEPES (10 mM) pH 7.0, 100 mM NaCl, 400 mM KCl.

[b] For experiments determining affinities for Fe^{II}, Co^{II}, Ni^{II} and Cu^{I} GTP was supplied at 100 μM, experiments to determine the Zn^{II} affinity of Mg^{II}GTP-YeiR and Mg^{II}GTPγS-YeiR were performed in the presence of 50 μM and 30 μM of nucleotide, respectively, to reduce interference with the detected species (nucleotides absorb strongly around $A_{269\ nm}$).

[c] fura-2, Mf2 and quin-2 form 1:1 M^{II}:ligand complexes with Co^{II}, Ni^{II} and Zn^{II}, respectively: at pH 7.0 fura-2 $K_{Co(II)}$ = 8.6 × 10^{-9} M (ref [1]); Mf2 $K_{Ni(II)}$ = 5.0 × 10^{-8} M (ref [9]); and quin-2 $K_{Zn(II)}$ = 3.7 × 10^{-12} M (ref [10]). Tar forms 1:2 Fe^{II}:ligand complexes at pH 7.0: $\beta_{2,Fe(II)}$ = 4.0 × 10^{13} M^{-2} (ref [3]). Bca forms a 1:2 Cu^{I}:ligand complex with β_2 = 1.6 × 10^{17} M^{-2} (ref [4]).

[d] Protein concentration in parenthesis reflects effective concentration of Mg^{II}GTP-YeiR or Mg^{II}GTPγS-YeiR present in the experiment, see Supplementary Fig. 20 legend.

[e] For Co^{II}fura-2 and Ni^{II}Mf2 probe responses were defined as variable parameters in Dynafit models. For $Fe^{II}(Tar)_2$ the probe response was fixed to the known extinction coefficient at pH 7.0; $\varepsilon_{720\ nm}$ = 19,000 M^{-1} cm^{-1} (ref [3]). The [quin-2] was determined by fitting a concurrently performed control titration, this value was then applied to fitting the experimental data; absorbance data was normalised to the highest and lowest values obtained over the course of the experiment.

[f] Negligible competition between probe Tar and protein for Fe^{II} binding, only a limiting affinity was determined (n = 1).

[g] $K_{Cu(I)}$ calculated via equation (9) (see methods).

[h] $K_{Zn(II)}$ determined when [Mg^{II}GTP-YeiR] or [Mg^{II}GTPγS-YeiR] were defined as variable parameters in Dynafit models, see Supplementary Fig. 20 legend.

Supplementary Table 7. Conditions of YjiA ligand competition experiments [a]

Metal (*M*)	Nucleotide [b]	Competing ligand (*L*) [c]	$[YjiA]_{tot}$ (μM)	$[L]_{tot}$ (μM)	$[M]_{max}$ (μM)	Species detected (signal)	K_D (M)	Figure ref.
Mn^{II}	GTPγS	Mf2	9.7	12	20	Mn^{II}mf2 ($A_{330\ nm}$ and $A_{365\ nm}$	≥1 × 10^{-4} [e]	S25a
			11	12	20		≥1 × 10^{-4} [e]	S25b
			11	13	20		≥1 × 10^{-4} [e]	S25c
			10	11	20		≥1 × 10^{-4} [e]	S24a
Fe^{II}	GTPγS	Tar	10	15	14	$Fe^{II}(Tar)_2$ [d] ($A_{720\ nm}$)	≥1 × 10^{-6} [f]	S24b
			11	16	16		≥1 × 10^{-6} [f]	S25f
			9.1	15	14		≥1 × 10^{-6} [f]	S25d
			10	18	14		≥1 × 10^{-6} [f]	S25e
Co^{II}	GTPγS	fura-2	9.9	10	24	Co^{II}fura-2 ($F_{510\ nm}$)	7.5 × 10^{-8}	S24c
			9.2	11	20		1.2 × 10^{-7}	S25g
			11	10	20		8.9 × 10^{-8}	S25h
			10	11	20		8.2 × 10^{-8}	S25i
Ni^{II}	GTPγS	Mf2	10	11	24	Ni^{II}mf2 ($A_{325\ nm}$ and $A_{365\ nm}$	1.2 × 10^{-7}	S24d
			9.9	13	20		1.4 × 10^{-7}	S25j
			10	12	20		1.8 × 10^{-7}	S25k
Cu^{I}	GTPγS	Bca	11	250	20	$Cu^{I}(Bca)_2$ ($A_{562\ nm}$)	5.9 × 10^{-16} [g]	S24e/S25l
			11	250	20		7.0 × 10^{-16} [g]	S24e/S25l
			11	250	40		8.6 × 10^{-16} [g]	S24e/S25l
			11	250	40		9.0 × 10^{-16} [g]	S24e/S25l
Zn^{II}	GTP	quin-2	11	8.7	27	Zn^{II}quin-2 ($A_{265\ nm}$)	1.5 × 10^{-12}	S25o
			10	8.5	27		2.2 × 10^{-12}	S24f
			10	8.6	27		6.2 × 10^{-12}	S25p
	GTPγS		10	11	27		3.7 × 10^{-12}	S24f
			11	11	30		2.7 × 10^{-12}	S25m
			11	9.1	27		4.8 × 10^{-12}	S25n

[a] All experiments conducted anaerobically in N_2-purged, chelex-treated HEPES (10 mM) pH 7.0, 100 mM NaCl, 400 mM KCl.
[b] Nucleotide supplied at 100 μM throughout.
[c] fura-2, Mf2 and quin-2 form 1:1 M^{II}:ligand complexes with Co^{II}, Ni^{II} and Mn^{II}, and Zn^{II}, respectively: at pH 7.0 fura-2 $K_{Co(II)}$ = 8.6 × 10^{-9} M (ref [1]); Mf2 $K_{Ni(II)}$ = 5.0 × 10^{-8} M (ref [9]); $K_{Mn(II)}$ = 6.1 × 10^{-6} M (ref[11]) and quin-2 $K_{Zn(II)}$ = 3.7 × 10^{-12} M (ref [10]). Tar forms 1:2 Fe^{II}:ligand complexes at pH 7.0: $\beta_{2,Fe(II)}$ = 4.0 × 10^{13} M^{-2} (ref [3]). Bca forms a 1:2 Cu^{I}:ligand complex with β_2 = 1.6 × 10^{17} M^{-2} (ref [4]).
[d] In fitting Tar competition data [Tar] was defined as a variable parameter in Dynafit models.
[e] Negligible competition between probe Mf2 and protein for Mn^{II} binding, only a limiting affinity was determined (n = 4).
[f] Negligible competition between probe Tar and protein for Fe^{II} binding, only a limiting affinity was determined (n = 4).
[g] $K_{Cu(I)}$ calculated via equation (9) (see methods) over the first 5 μM of Cu^{I} addition to determine $K_{Cu(I)}$ for the tightest binding event.

Supplementary Table 8. Calculations of conditional intracellular available [Co^{II}] (and corresponding $\Delta G_{Co(II)}$) for *E. coli** cultures in Co^{II}-supplemented LB media

[Co^{II}] (μM)	θ_D (RcnR)	[Co^{II}]$_{buffered}$ (M)	$\Delta G_{Co(II)}$ (kJ mol^{-1})
0 [a]	0.99	2.4×10^{-11}	-60.6
1	0.92	2.2×10^{-10}	-55.1
3	0.85	4.1×10^{-10}	-53.6
10	0.57	1.9×10^{-9}	-49.8
30	0.28	6.7×10^{-9}	-46.6
300 [a]	0.01	2.7×10^{-7}	-37.5

[a] Defined as limits of RcnR dynamic response range (see Supplementary Fig. 27).

Supplementary Table 9. Total metal in *E. coli** [a]

[Co] in media (μM)	Co (atoms/cell) +*cobW*	Co (atoms/cell) -*cobW*	Zn (atoms/cell) +*cobW*	Zn (atoms/cell) -*cobW*
0	0.96 (±0.07) × 10^3	0.47 (±0.04) × 10^3	3.8 (±0.1) × 10^5	4.8 (±0.1) × 10^5
1	21 (±1) × 10^3	11 (±0.2) × 10^3	4.2 (±0.1) × 10^5	4.6 (±0.2) × 10^5
3	38 (±4) × 10^3	23 (±2) × 10^3	4.5 (±0.2) × 10^5	5.1 (±0.7) × 10^5
10	88 (±3) × 10^3	69 (±0.9) × 10^3	4.6 (±0.1) × 10^5	4.5 (±0.1) × 10^5
30	159 (±9) × 10^3	154 (±7) × 10^3	5.9 (±0.2) × 10^5	4.4 (±0.3) × 10^5
300	1.0 (±0.1) × 10^6	1.2 (±0.1) × 10^6	4.4 (±0.2) × 10^5	4.3 (±0.1) × 10^5

[a] Data are the mean ± SD of n = 3 biologically independent replicates

Supplementary Table 10. Oligonucleotides used in this work

No.	Primer Name	Sequence	Reference
1	Rc_cobW_F	5’-CATCATATGTCCGATCTGACCAAAATCC-3'	This work
2	Rc_cobW_R	5’-CATACTAGTCGACATGCATCAGGCGGC-3’	This work
3	Ec_rcnA_F	5’-GAACCAGGGCACTCAAAAAC-3’	ref.[12]
4	Ec_rcnA_R	5’-TGCGGTATGCGAAATAGTTG-3’	ref.[12]
5	Ec_zntA_F	5’-TCCGGCAACGGGTATTAGTG-3’	This work
6	Ec_zntA_R	5’-GTTCAGCAACCTGTGCTTCG-3’	This work
7	Ec_znuA_F	5’-GTTTGGACTGACACCGCTTG-3’	This work
8	Ec_znuA_R	5’-ACGCAGGTTGCTTTTTGCTC-3’	This work
9	Ec_rpoD_F	5’-GTGGCTTGCAGTTCCTTGAC-3’	This work
10	Ec_rpoD_R	5’-AGGTTGCGTAGGTGGAGAAC-3’	This work

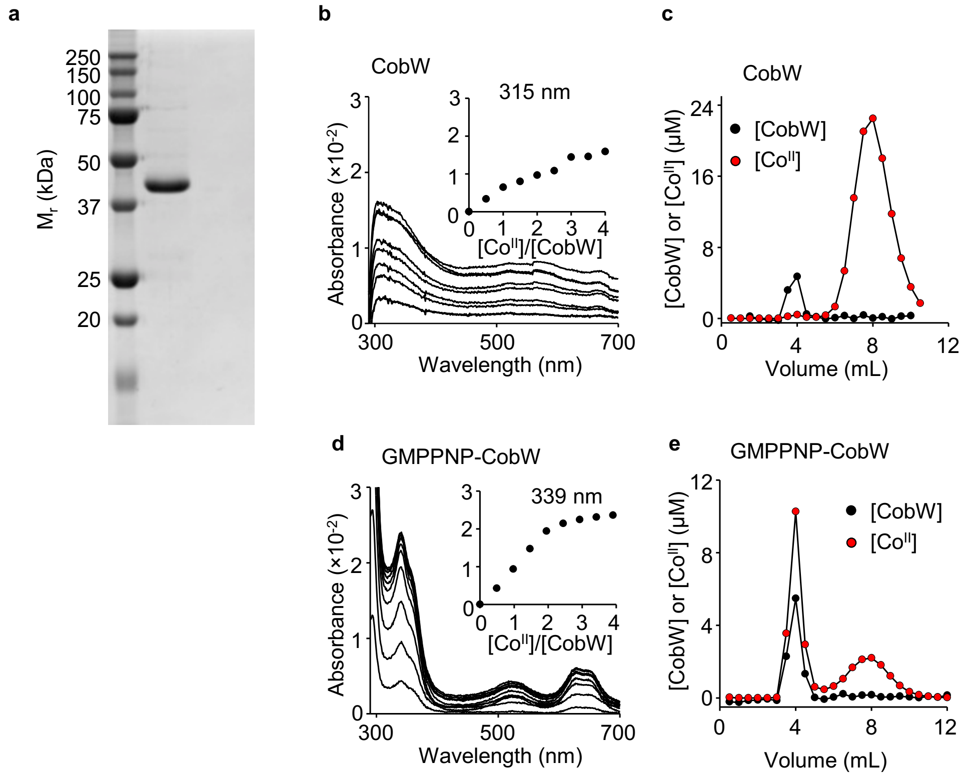

Supplementary Fig. 1. Source files and replicate data for Fig. 1. a Full gel image from Fig. 1b. **b** Replicate data for Fig. 1d (20 μM CobW). **c** Replicate data for Fig. 1e (10 μM CobW incubated with 100 μM Co^{II}). [CobW] and [Co^{II}] detected in each fraction are shown in black and red, respectively. **d** Replicate data for Fig. 1g (12 μM CobW, 30 μM GMP-PNP). **e** Replicate data for Fig. 1h (identical conditions). [CobW] and [Co^{II}] detected in each fraction are shown in black and red, respectively.

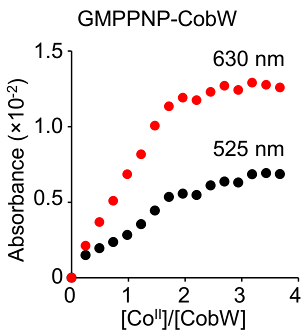

Supplementary Fig. 2. Visible absorbance features from Fig. 1g. Absorbance of features at 525 nm (black circles) and 630 nm (red circles) from Fig. 1g show a linear increase saturating at 2:1 ratio Co^{II}:CobW.

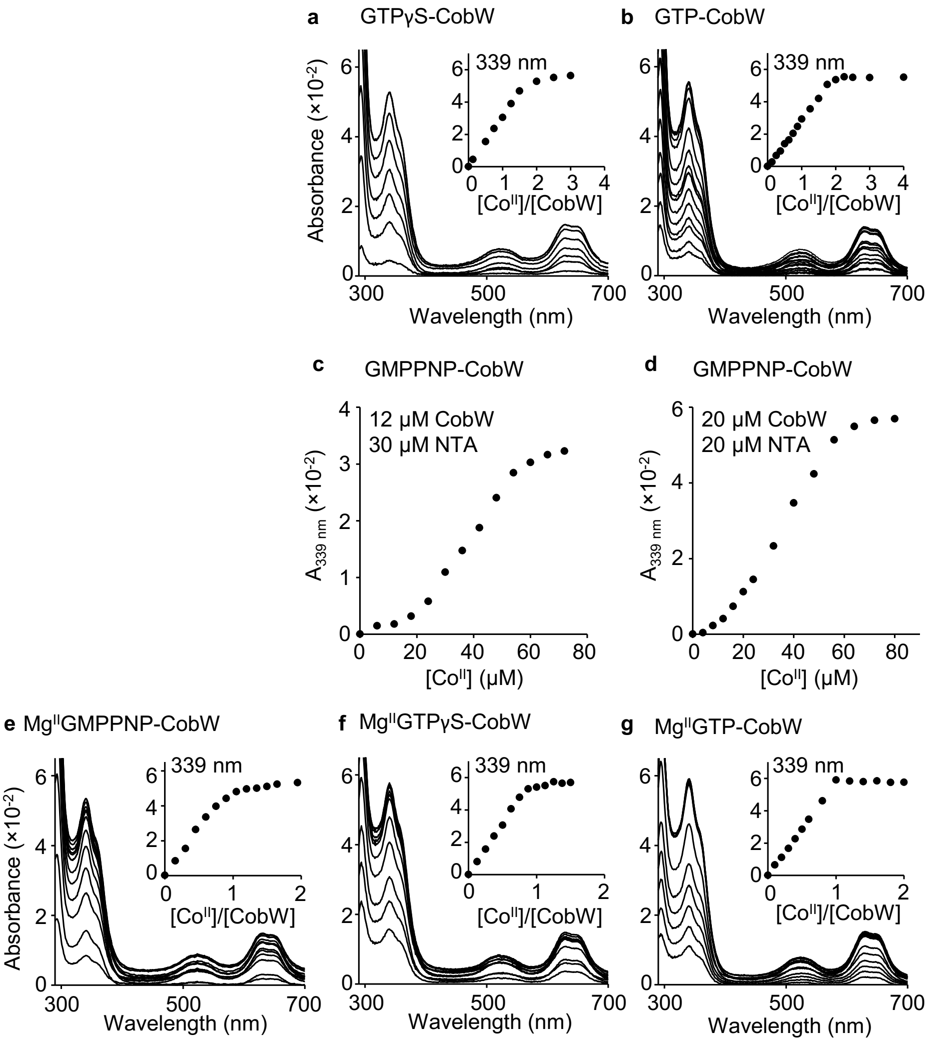

Supplementary Fig. 3. Different GTP analogues assemble similar metal-binding sites in CobW. a-b Representative apo-subtracted spectra of Co^{II}-titrated CobW (20 μM) in the presence of **a** 60 μM GTPγS or **b** 200 μM GTP; features at 339 nm (insets) show linear increase saturating at 2:1 ratio Co^{II}:CobW. **c-d** Apo-subtracted absorbance of Co^{II}-titrated CobW in competition with NTA (20 - 30 μM as indicated) in the presence of excess GMPPNP (30, 60 μM in c, d respectively). Together with Fig. 2a these data complete n = 3 independent titrations showing cooperative binding of the two metal-sites in CobW (in the absence of Mg^{II}). **e** Replicate data for Fig 2b. **f** As in **a** with added Mg^{II} (2.7 mM). **g** As in **b** with added Mg^{II} (2.7 mM). In **e-g** the feature at 339 nm (inset) shows a linear increase saturating at 1:1 ratio Co^{II}:CobW.

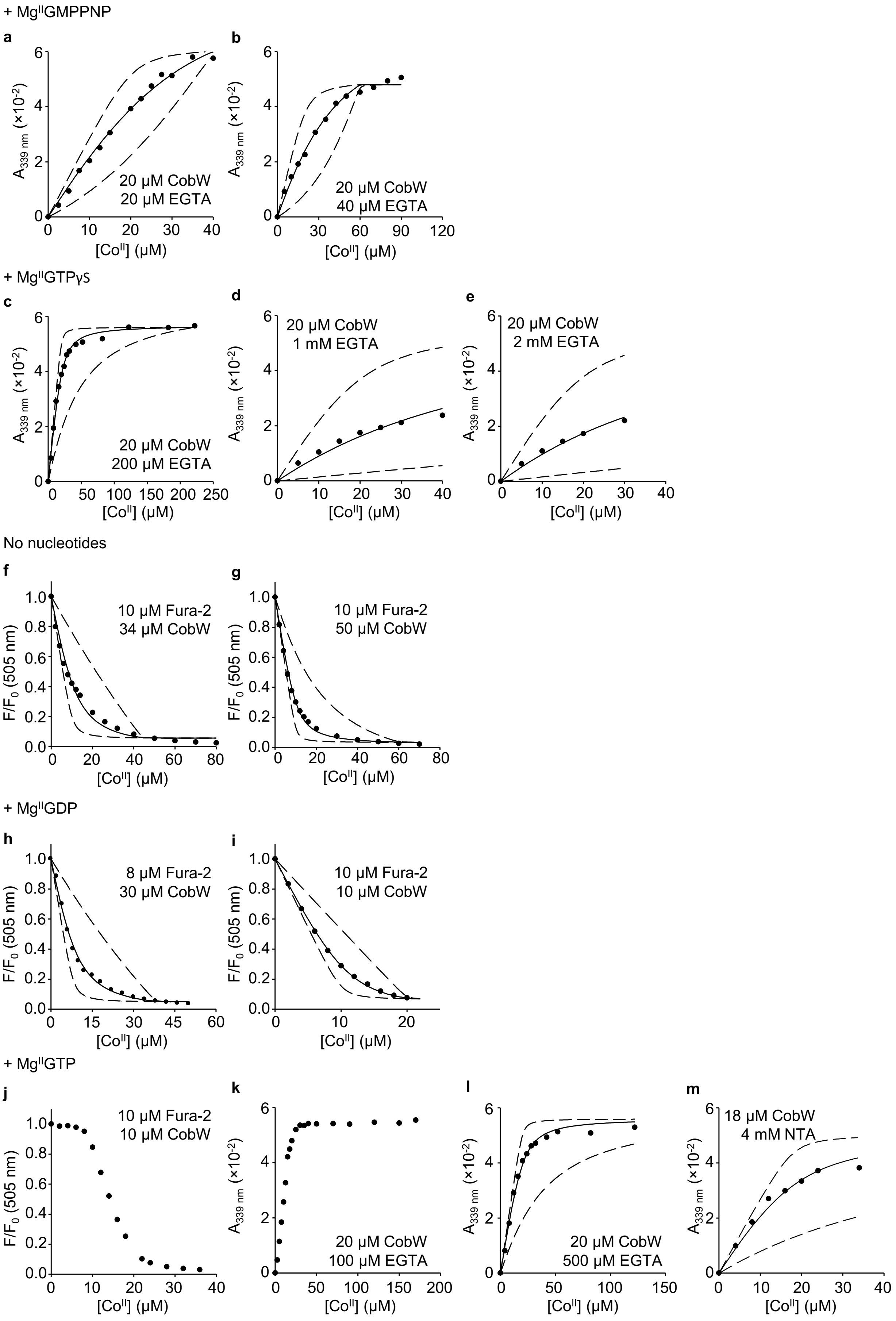
+ Mg^II GMPPNP
a
20 µM CobW
20 µM EGTA
b
20 µM CobW
40 µM EGTA
+ Mg^II GTPγS
c
20 µM CobW
200 µM EGTA
d
20 µM CobW
1 mM EGTA
e
20 µM CobW
2 mM EGTA
No nucleotides
f
10 µM Fura-2
34 µM CobW
g
10 µM Fura-2
50 µM CobW
+ Mg^II GDP
h
8 µM Fura-2
30 µM CobW
i
10 µM Fura-2
10 µM CobW
+ Mg^II GTP
j
10 µM Fura-2
10 µM CobW
k
20 µM CobW
100 µM EGTA
l
20 µM CobW
500 µM EGTA
m
18 µM CobW
4 mM NTA
A339 nm (×10-2)
F/F0 (505 nm)
[Co^II] (µM)

Supplementary Fig. 4. $K_{Co(II)}$ quantification for CobW, in the absence or presence of nucleotides, using a variety of competing conditions. a-e, k-m Change in absorbance at 339 nm (with respect to Co^{II}-free solution) when Co^{II} was titrated into CobW in competition with EGTA or NTA (as specified in each panel) in the presence of Mg^{II} (2.7 mM) and nucleotide (**a-b**) GMPPNP (60 μM), (**c-e**) GTPγS (60 μM), or (**k-m**) GTP (10-fold excess of protein concentration). **f-j** Fluorescence quenching of Co^{II}-titrated fura-2 in competition with (**f-g**) CobW alone, (**h-i**) CobW in the presence of Mg^{II} (2.7 mM) and GDP (10-fold excess of protein concentration), (**j**) CobW in the presence of Mg^{II} (2.7 mM) and GTP (100 μM). In **a-i**, **l-m** solid traces show curve fits of experimental data to models where CobW binds one molar equivalent Co^{II} per protein monomer. Dashed lines show simulated responses for $K_{Co(II)}$ tenfold tighter or weaker than the fitted value. In **j, k** Co^{II} was almost entirely withheld by Mg^{II}GTP-CobW, preventing meaningful determination of $K_{Co(II)}$ under these conditions. Further details and fitted $K_{Co(II)}$ values are listed in Supplementary Table 1.

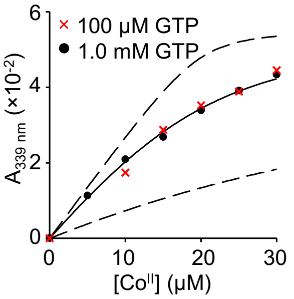

Supplementary Fig. 5. $K_{Co(II)}$ quantification for Mg^{II}GTP-CobW at varying nucleotide concentrations. Change in absorbance at 339 nm (relative to Co^{II}-free solution) when Co^{II} was titrated into CobW (20 µM) in competition with EGTA (2.0 mM) in the presence of Mg^{II} (2.7 mM) and GTP (100 µM or 1.0 mM; red crosses and black circles, respectively). Experiments performed in 50 mM HEPES pH 7.0, 100 mM NaCl, 400 mM KCl. Solid trace shows representative curve fit of the experimental data (for 1.0 mM GTP dataset) to a model where CobW binds one molar equivalent Co^{II} per protein monomer. Dashed lines show simulated responses for $K_{Co(II)}$ tenfold tighter or weaker than the fitted value. At both nucleotide concentrations the measured $K_{Co(II)}$ (1.8 × 10^{-11} M and 1.9 × 10^{-11} M) were within experimental error.

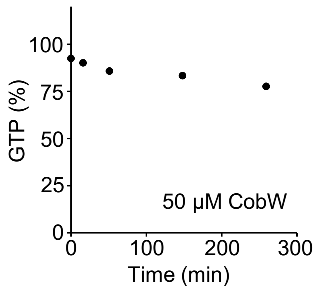

Supplementary Fig. 6. CobW-catalysed GTP hydrolysis is also slow at 4:1 ratio GTP:CobW. Analysis of GTP hydrolysis when a solution of GTP (200 µM) was incubated with CobW (50 µM), Mg^{II} (2.7 mM) and Co^{II} (45 µM). Nucleotides were separated by anion-exchange and detected by UV absorbance (280 nm).

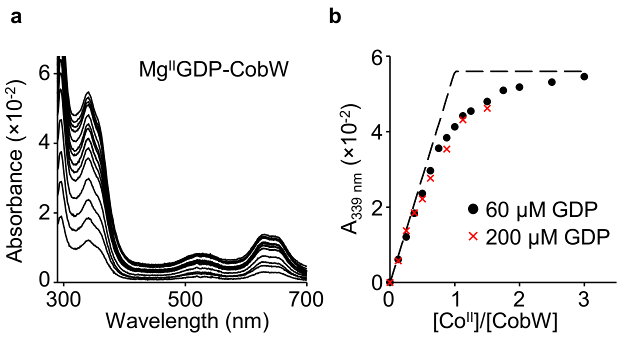

Supplementary Fig. 7. Mg^{II}GDP-CobW has a similar Co^{II} site to Mg^{II}GTP-CobW but with weaker $K_{Co(II)}$. a Apo-subtracted spectra of Co^{II}-titrated CobW (20 μM) in the presence of Mg^{II} (2.7 mM) and GDP (60 μM) showed that Mg^{II}GDP-CobW possesses a similar cysteine-rich, tetrahedral Co^{II} site to that of Mg^{II}GTP-CobW (*cf* Supplementary Fig. 3g). **b** Absorbance feature from (**a**) at 339 nm (black circles) lacks a sharp turning point at 1 equivalent Co^{II}:CobW suggesting competition between the tetrahedral site in Mg^{II}GDP-CobW and weak-binding solution components (*eg* buffer, salts or alternative Co^{II} sites within CobW itself). An equivalent experiment with 200 μM GDP (red crosses) gave indistinguishable results, showing that nucleotide concentration was not a limiting factor for metal-binding. Dotted trace shows simulated response assuming the same extinction coefficient for Co^{II}MgGDP-CobW as for other nucleotide-bound forms (GMPPNP, GTPγS and GTP).

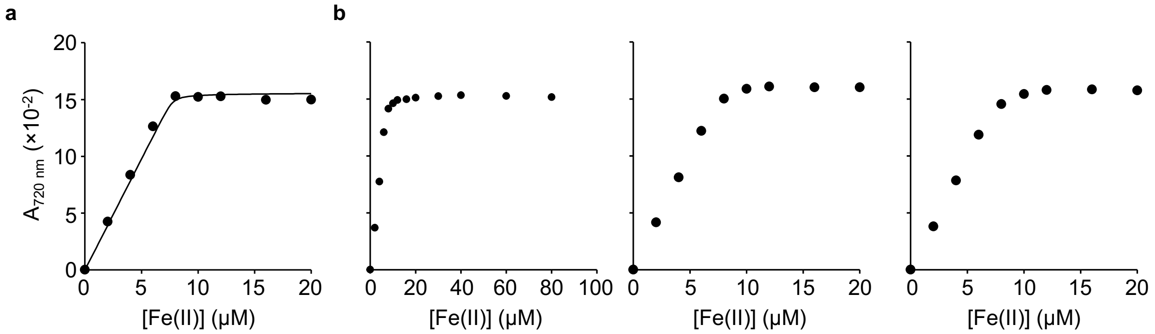

Supplementary Fig. 8. Further details of $K_{Fe(II)}$ quantification for Mg^{II}GTP-CobW using Tar. a Titration of Tar (16 μM) with Fe^{II} in buffer only. Solid trace shows fitted extinction coefficient of $\varepsilon_{720\ nm} = 1.956 \times 10^4\ M^{-1}\ cm^{-1}$ assuming stoichiometric $Fe^{II}Tar_2$ formation (in agreement with reported value at pH 7.0 (ref.[3])). **b** Source data for Fig. 3a showing entire collected dataset (up to 80 μM added Fe^{II}), and two replicated experiments (with up to 20 μM added Fe^{II}) confirming that Mg^{II}GTP-CobW (50 μM) cannot compete with Tar (16 μM) for binding Fe^{II}.

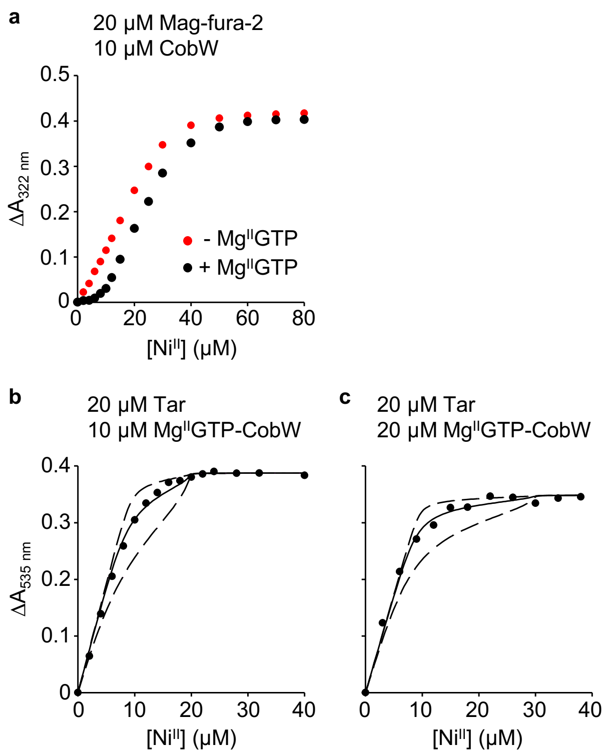

Supplementary Fig. 9. Further details of $K_{Ni(II)}$ quantification for Mg^{II}GTP-CobW. a Absorbance change at 322 nm upon Ni^{II}-titration of CobW (10 µM) in competition with Mf2 (20 µM) with or without Mg^{II} (2.7 mM) and GTP (100 µM). In the absence of Mg^{II}GTP (red circles), CobW binds two Ni^{II} ions with a similar affinity to Mf2 ($K_{Ni(II)} = 5 \times 10^{-8}$ M (ref.[9])); but in the presence of Mg^{II}GTP (black circles), CobW shows an additional Ni^{II} site which outcompetes Mf2. **b-c** Absorbance change (relative to Ni^{II}-free solution) upon Ni^{II}-titration of Tar in competition with CobW in the presence of Mg^{II} (2.7 mM) and GTP (100 µM in **b** and 200 µM in **c**). In **b-c** solid traces show curve fits of experimental data to models where CobW binds one molar equivalent Ni^{II} per protein monomer. Dashed lines show simulated responses for $K_{Ni(II)}$ tenfold tighter or weaker than the fitted value. Fitted $K_{Ni(II)}$ values are listed in Supplementary Table 1.

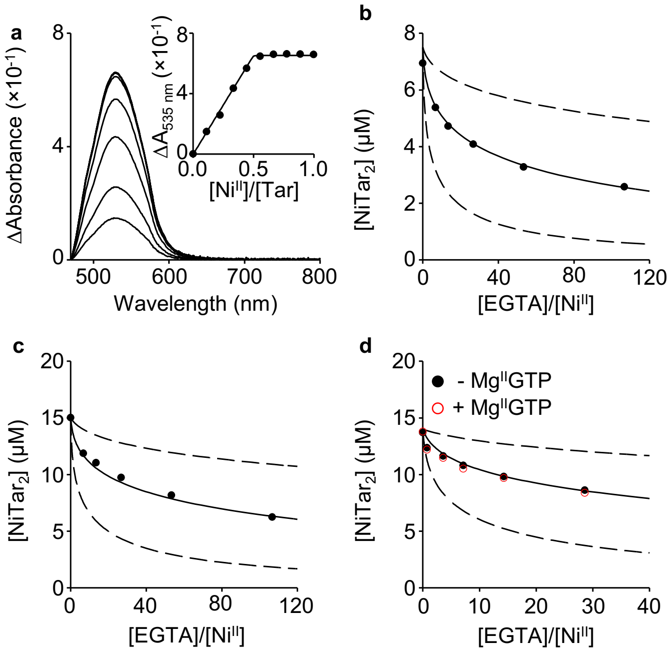

Supplementary Fig. 10. Determination of β_2 for $Ni^{II}Tar_2$ at pH 7.0. a Representative (n = 3) change in absorbance (relative to metal-free probe) upon Ni^{II}-titration of Tar (34 μM). Inset shows linear increase of feature at 535 nm saturating at 1:2 ratio Ni^{II}:Tar; and solid trace shows fitted extinction coefficient of ε = 3.8 (±0.1) × 10^4 M^{-1} cm^{-1} (mean ± SD of n = 3 experiments) assuming stoichiometric $Ni^{II}Tar_2$ formation. **b**-**d** Addition of increasing [EGTA] into probe solutions containing (**b**) 7.5 μM Ni^{II}, 18 μM Tar; (**c**) 15 μM Ni^{II}, 36 μM Tar; and (**d**) 14 μM Ni^{II}, 36 μM Tar; shows competition for Ni^{II}-binding at equilibrium (black circles). Solid traces are curve fitting of experimental data to equation (5) (Methods) resulting in β_2 = 4.3 (±0.6) × 10^{15} M^{-2} for $Ni^{II}Tar_2$ formation at pH 7.0 (mean ± SD from n = 3 experiments in **b**-**d**). Dashed lines are models of binding 10-fold tighter or weaker than measured values. Addition of Mg^{II} (2.7 mM) and GTP (500 μM) to solutions in **d** (open red circles) had negligible effect on Ni^{II}-binding equilibria.

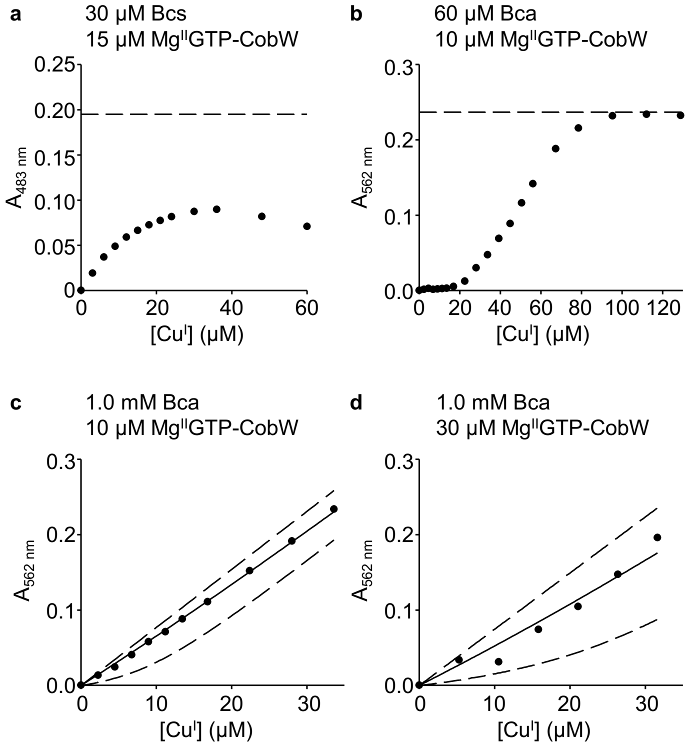

Supplementary Fig. 11. Further details of $K_{Cu(I)}$ quantification for Mg^{II}GTP-CobW. a Cu^{I}-titration into a mixture of Bcs (30 μM), CobW (15 μM), Mg^{II} (2.7 mM) and GTP (200 μM) does not reach theoretical absorbance maximum for complete formation of $Cu^{I}Bcs_2$ (dashed line, $\varepsilon_{483\ nm}$ = 13,000 M^{-1} cm^{-1} (ref.[2])) at saturating [Cu^{I}] which is suggestive of ternary complex formation[13]. **b** Absorbance (562 nm) upon Cu^{I}**-**titration into a mixture of Bca (60 μM), CobW (10 μM), Mg^{II} (2.7 mM) and GTP (200 μM) shows that the protein binds 2 equivalents Cu^{I} with tighter affinity than Bca and a further 3 equivalents Cu^{I} with sufficient affinity to compete with the probe; theoretical absorbance maximum for complete formation of $Cu^{I}Bca_2$ (dashed line, $\varepsilon_{562\ nm}$ = 7,900 M^{-1} cm^{-1} (ref.[2])) is reached upon addition of ~ 80 μM Cu^{I} (*ie* sufficient to saturate both probe Bca and ~ 5 protein sites). **c-d** Absorbance of Cu^{I}-titrated Bca (1.0 mM) in competition with (**c**) 10 μM CobW or (**d**) 30μM CobW in the presence of Mg^{II} (2.7 mM) and GTP (200 μM). In **c-d** solid traces are curve fits of experimental data to model assuming only the tightest site in CobW binds Cu^{I} at the limiting availabilities employed (fitted $K_{Cu(I)}$ in Supplementary Table 1); dashed lines show simulated responses for $K_{Cu(I)}$ tenfold tighter or weaker than the fitted value.

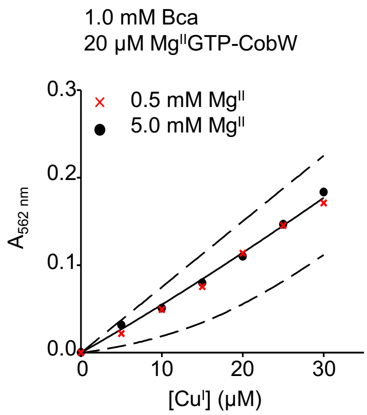

Supplementary Fig. 12. Effect of Mg^{II} on Cu^{I}-competition experiments using Bca. Cu^{I}-competition experiment as in Fig. 3c but with varying [Mg^{II}] (0.5 and 5.0 mM shown by red crosses and black circles, respectively). Solid trace shows representative curve fit of the experimental data (for 5.0 mM Mg^{II} dataset) to a model where CobW binds one molar equivalent Cu^{I} per protein monomer. Dashed lines show simulated responses for $K_{Cu(I)}$ tenfold tighter or weaker than the fitted value. At both Mg^{II} concentrations the fitted $K_{Cu(I)}$ (2.5 × 10^{-16} M and 2.2 × 10^{-16} M) were the same (within experimental error) as the value determined at cellular [Mg^{II}] (Supplementary Table 2). Thus, the presence of cellular [Mg^{II}] had negligible effect on the Cu^{I}-binding equilibria in these experiments.

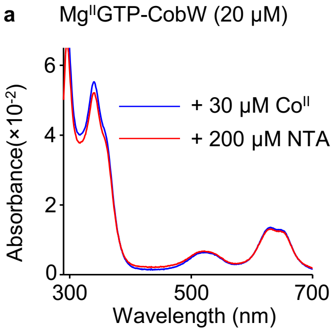

b

$$Co^{II}L + P \underset{}{\overset{K_{ex}}{\rightleftharpoons}} Co^{II}P + L$$

$$\frac{[Co^{II}P]}{[P]} = \frac{K_{CoII}(L)}{K_{CoII}(P)} \times \frac{[Co^{II}L]}{[L]}$$

$$= 733 \times \frac{[Co^{II}L]}{[L]}$$

where L = NTA, P = Mg^{II}GTP-CobW

Supplementary Fig. 13. Mg^{II}GTP-CobW is metal-saturated in Fig. 4 inter-metal competition experiments. a Change in absorbance of Mg^{II}GTP-CobW (20 µM) upon addition of 30 µM Co^{II} (blue trace), followed by 200 µM NTA (red trace) at equilibrium. **b** Reaction describing Co^{II} exchange between NTA and Mg^{II}GTP-CobW, and corresponding relationships valid at equilibrium. In **a**, addition of Co^{II} leads to stoichiometric formation of Co^{II}Mg^{II}GTP-CobW (20 µM); addition of NTA reduces $A_{339\ nm}$ to 95% of original intensity at equilibrium ratio of [Co^{II}NTA]/[NTA] = 0.06 consistent with the predicted 2% dissociation of Co^{II} from the protein complex (calculated using the relationships in **b**) plus a dilution factor (2%) from NTA addition. The measured equilibria in Fig. 4 were all conducted at ratios [Co^{II}NTA]/[NTA] > 0.06 thus the high affinity site of Mg^{II}GTP-CobW is > 95% metalated (with either Co^{II} or Zn^{II}) at all tested conditions.

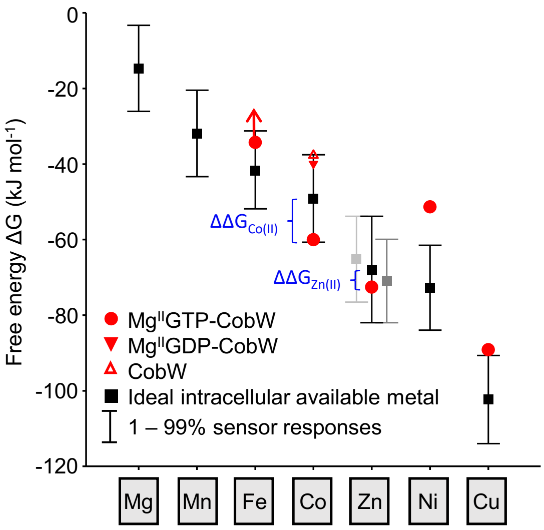

Supplementary Fig. 14. The accessible range of intracellular available free energies for metal-binding as sensors shift from 1 – 99% of their responses. As in Fig. 5 except bars show the change in intracellular available Δ*G* as cognate sensors shifts from 1-99% of their responses. Free-energy change (Δ*G*) for metal-binding to CobW alone (open red triangle), Mg^{II}GDP-CobW (closed red triangle) and Mg^{II}GTP-CobW (red circles) plotted against the intracellular available free energies for metal-binding under idealised conditions (black squares). Free energy differences (ΔΔ*G*) which favour acquisition of metals by Mg^{II}GTP-CobW *in vivo* are indicated in blue. For Fe^{II} binding to Mg^{II}GTP-CobW, arrow indicates limiting $\Delta G > -34.2$ kJ mol^{-1}.

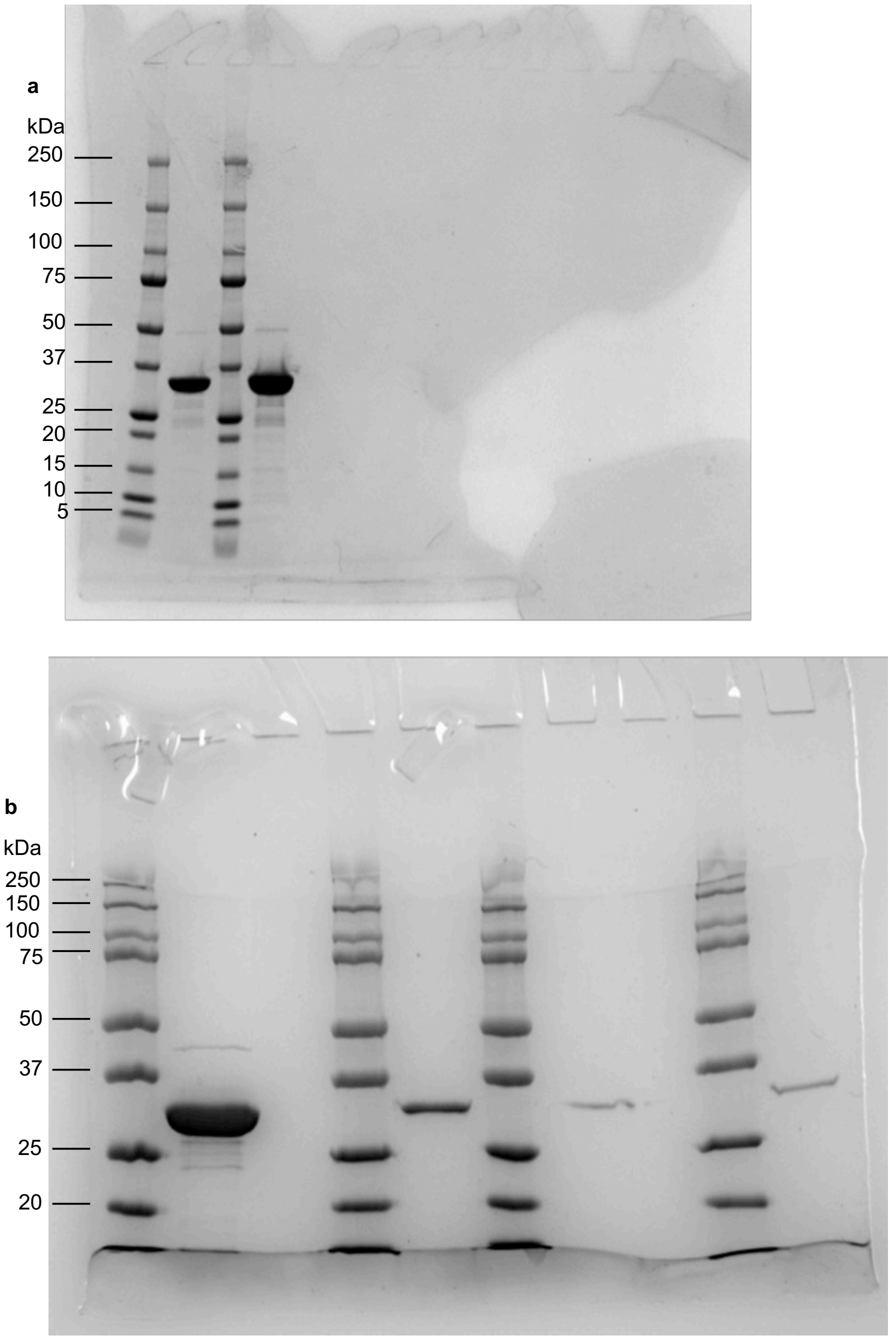

Supplementary Fig. 15. SDS-PAGE analysis of purified YeiR and YjiA. Variable loading of purified proteins **a** YeiR (n = 1 under these conditions), and **b** YjiA (n = 1 under these conditions) alternated with Precision Plus Protein[TM] Dual Xtra Prestained Protein Standards (BioRad)**.**

TCGGCTGCGCCCCAGGGCGCAGTCTGCCCCTTTTATTTATTGACTGTTCTGCACCAGGCA
AGAGAAACGTTAAAAATTTAACATTTTCTGCTCTTAAGATGGCAGCAGCACGACTTCGTC
AACTCAGCACTTCTCTTTTTCACGTTTATTCGCCAGGATTATTAA
AAGTTGTTATGTTGTATCAATATCCTGGAGCTGAC GTG

RNNNYRNNRYNNYRNNNY
YNNNRYNNYRNNRYNNNY

Supplementary Fig. 16. Zur recognition sequences in the promoter region of *yeiR*. DNA sequence 200 bp upstream of *yeiR* start codon (green) with Zur recognition sequence[14] shown in red along with the complementary sequence shown in blue. Identified regions of sequence similarity shown in boxes.

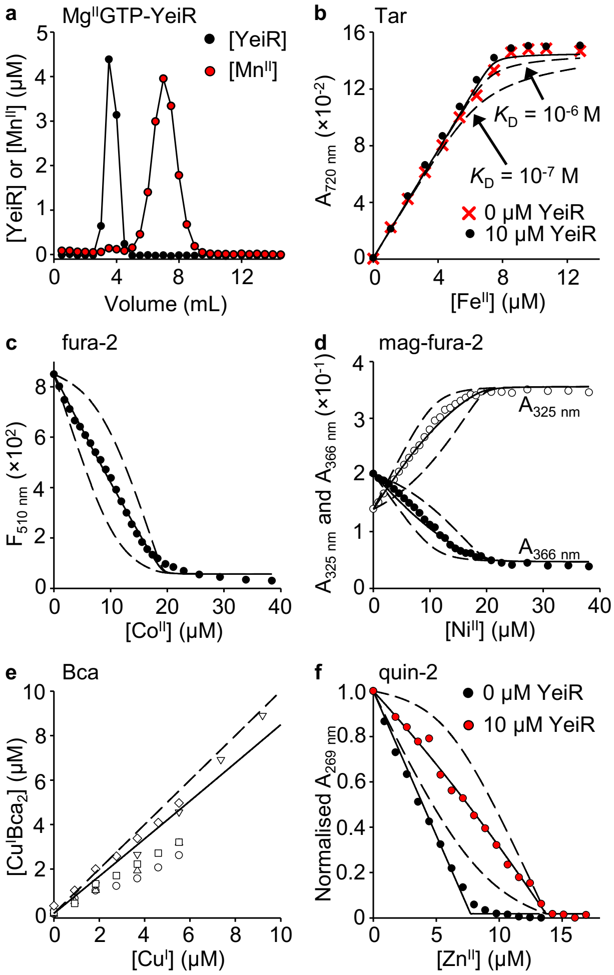

Supplementary Fig. 17. Metal affinities of Mg^{II}GTP-YeiR. a Elution profile following gel filtration of a mixture of YeiR (10 µM), GTP (100 µM), Mg^{II} (2.7 mM) and Mn^{II} (20 µM) showing no co-migration of metal with protein. Fractions were analysed for protein (black) by Bradford assay and for metal (red) by ICP-MS (n = 1). **b** Absorbance change upon Fe^{II}-titration into a mixture of Tar (15 µM), Mg^{II} (2.7 mM) and GTP (100 µM) in the absence (red crosses) or presence (black circles) of YeiR (10 µM). The experimental data overlays the control titration with Tar alone indicating Tar completely outcompetes Mg^{II}GTP-YeiR for Fe^{II}. Dashed lines show simulated responses for specified $K_{Fe(II)}$ of Mg^{II}GTP-YeiR, providing limiting $K_{Fe(II)} \geq 10^{-6}$ M (n = 1). **c** Representative (n = 3) fluorescence emission quenching of fura-2 (10 µM) in the presence of YeiR (8.9 µM), GTP (100 µM) and Mg^{II} (2.7 mM) upon titration with Co^{II}. Solid line represents a fit to a 1:1 binding model with the dashed lines representing simulated fits to affinities 10-fold tighter and weaker than the fitted value. **d** Representative (n = 3) absorbance change of Mf2 (11 µM) at 325 nm (open circles) and 366 nm (closed circles) upon titration with Ni^{II} in the presence of YeiR (8.9 µM), GTP (100 µM) and $MgCl_2$ (2.7 mM). Solid lines represent a fit to a 1:1 binding model with the dashed lines representing simulated fits to affinities 10-fold tighter and weaker than the fitted value. **e** Concentration of $Cu^{I}Bca_2$ formed upon titration of Cu^{I} into a solution of Bca (800 µM), YeiR (10 µM), GTP (100 µM) and Mg^{II} (2.7 mM). Absorbance at 562 nm was measured at equilibrium and converted to [$Cu^{I}Bca_2$]. Different shapes represent different experiments (n = 5). Dashed line represents expected [$Cu^{I}Bca_2$] in the absence of Mg^{II}GTP-YeiR and solid line is a simulation of the Mg^{II}GTP-YeiR $K_{Cu(I)}$ calculated via equation (9) (see methods). **f** Representative (n = 4) normalised absorbance change of quin-2 (7.7 µM) upon titration with Zn^{II} in the absence (in black) or presence (in red) of YeiR (10 µM). GTP (50 µM) and Mg^{II} (2.7 mM) present in both experiments. Solid black line shows fit to a 1:1 binding model where [Mg^{II}GTP-YeiR] is a fitted parameter (fitted

as 6.0 µM, see Supplementary Fig. 20). Dashed lines represent simulations 10-fold tighter and weaker than the fitted value. Replicate experiments for **c-d, f** shown in Supplementary Fig. 19.

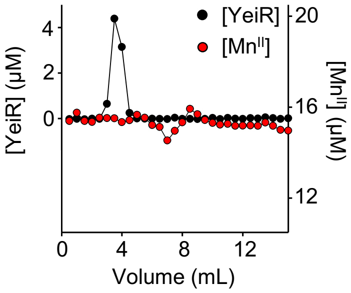

Supplementary Fig. 18. Additional data for Supplementary Fig. 17a. Elution profile following gel filtration of a mixture of YeiR (10 μM), GTP (100 μM), Mg^{II} (2.7 mM) and Mn^{II} (20 μM), where running buffer was additionally supplemented with Mn^{II} (20 μM), showing no co-migration of protein with metal. Fractions were analysed for protein (black) by Bradford assay and for metal (red) by ICP-MS (n = 1).

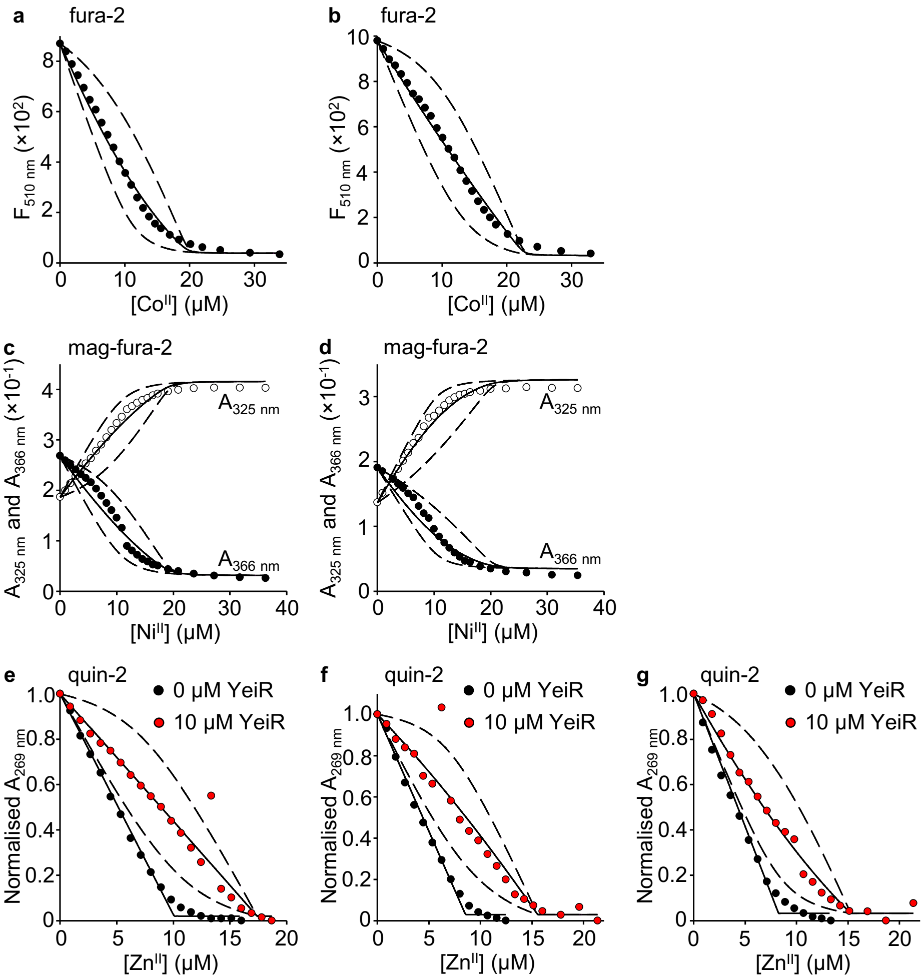

Supplementary Fig. 19. Replicate data for Supplementary Fig. 17c,d and f. a-b Replicates of experiment shown in Supplementary Fig. 17c with **a** [fura-2] = 11 μM, [YeiR] = 8.9 μM, [GTP] = 100 μM, [Mg^{II}] = 2.7 mM; and **b** [fura-2] = 13 μM, [YeiR] = 10 μM, [GTP] = 100 μM, [Mg^{II}] = 2.7 mM. **c-d** Replicates of experiment shown in Supplementary Fig. 17d with **c** [Mf2] = 11 μM, [YeiR] = 8.9 μM, [GTP] = 100 μM, [Mg^{II}] = 2.7 mM; and **d** [Mf2] = 11 μM, [YeiR] = 10 μM, [GTP] = 100 μM, [Mg^{II}] = 2.7 mM. Absorbance at 325 nm and 366 nm shown by open and closed circles, respectively. **e-g** Replicates of experiment shown in Supplementary Fig. 17f with **e** [quin-2] = 10 μM, [YeiR] = 10 μM, [GTP] = 50 μM, [Mg^{II}] = 2.7 mM, fitted [Mg^{II}GTP-YeiR] = 7.3 μM; and **f** [quin-2] = 8.6 μM, [YeiR] = 10 μM, [GTP] = 50 μM, [Mg^{II}] = 2.7 mM, fitted [Mg^{II}GTP-YeiR] = 6.0 μM; and **g** [quin-2] = 8.3 μM, [YeiR] = 10 μM, [GTP] = 50 μM, [Mg^{II}] = 2.7 mM, fitted [Mg^{II}GTP-YeiR] = 5.7 μM. Titrations in the absence and presence of YeiR are shown in black and red, respectively.

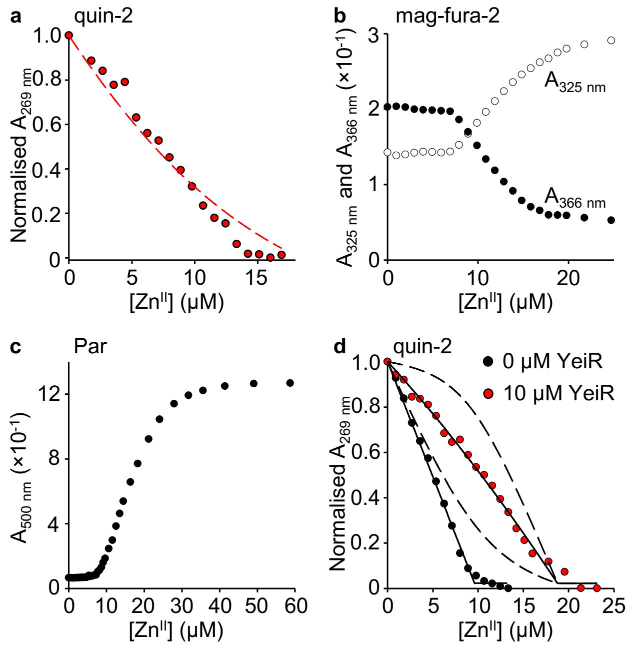

Supplementary Fig. 20. Fitting of Zn^{II} binding data for Mg^{II}GTP-YeiR. a Reproduction of data shown in Supplementary Fig. 17f. Red dashed line shows fit to a 1:1 binding model where [Mg^{II}GTP-YeiR] is a fixed rather than a fitted parameter (as in Supplementary Fig. 17f). **b** Representative (n = 5) absorbance change of Mf2 (11 µM) at 325 nm (open circles) and 366 nm (closed circles) upon titration with Zn^{II} in the presence of YeiR (10 µM), GTP (100 µM) and Mg^{II} (2.7 mM). **c** Absorbance change of Par (40 µM) upon titration with Zn^{II} in the presence of YeiR (10 µM), GTP (100 µM) and Mg^{II} (2.7 mM) (n = 1). **d** Representative (n = 3) normalised absorbance change of quin-2 (9.6 µM) in the absence (black) and presence (red) of YeiR (10 µM). GTPγS (30 µM) and Mg^{II} (2.7 mM) were present in both experiments. Solid black line shows fit to a 1:1 binding model where [Mg^{II}GTPγS-YeiR] is a fitted parameter (fitted as 9.2 µM). Dashed lines represent simulations 10-fold tighter and weaker than the fitted value. Initial attempts to fit the competition data between quin-2 and Mg^{II}GTP-YeiR for Zn^{II} as a 1:1 binding model where [Mg^{II}GTP-YeiR] is a fixed parameter generated a poor fit to the data (Supplementary Figs. 20a and 21a-c). Setting [Mg^{II}GTP-YeiR] as a fitted parameter in the fitting produces a better fit to the data (Supplementary Figs. 17f and 19e-g) determining a $K_{Zn(II)}$ of 3.0 (±1) ×10^{-12} M and a zinc binding stoichiometry of 0.62 (±0.07) with a range of 0.57-0.73. Mg^{II}GTP-YeiR withholds Zn^{II} from the weaker Zn^{II} chelators Mf2 and Par (Supplementary Figs. 20b-c and 21d-g) but inflection points at less than one molar equivalent reveal sub-stoichiometric Zn^{II}-binding. Across the six competition experiments Mg^{II}GTP-YeiR withheld 0.7 (±0.06) molar equivalents (with a range of 0.59 – 0.89) of Zn^{II} from Mf2 and Par. Sub-stoichiometric Zn^{II} binding by Mg^{II}GTP-YeiR may be reporting on hydrolysis of GTP over the course of the experiments. Competition with quin-2 was repeated using the non-hydrolysable GTP analogue GTPγS (Supplementary Figs. 20d and 21h-i). These data were fit with [Mg^{II}GTPγS-YeiR] as a fitted parameter determining a Zn^{II} affinity of 4.1 (±3) ×10^{-12} M and a zinc binding stoichiometry of 0.97 (±0.05) with a range of 0.92-1.0. These analyses suggest that GTP may be partially hydrolysed over the course of Zn^{II} competition experiments, that the Zn^{II} affinities determined using GTP or GTPγS are within error of each other suggests this does not impact the Zn^{II} affinity determined. Replicate experiments for **a-b, d** shown in Supplementary Fig. 21.

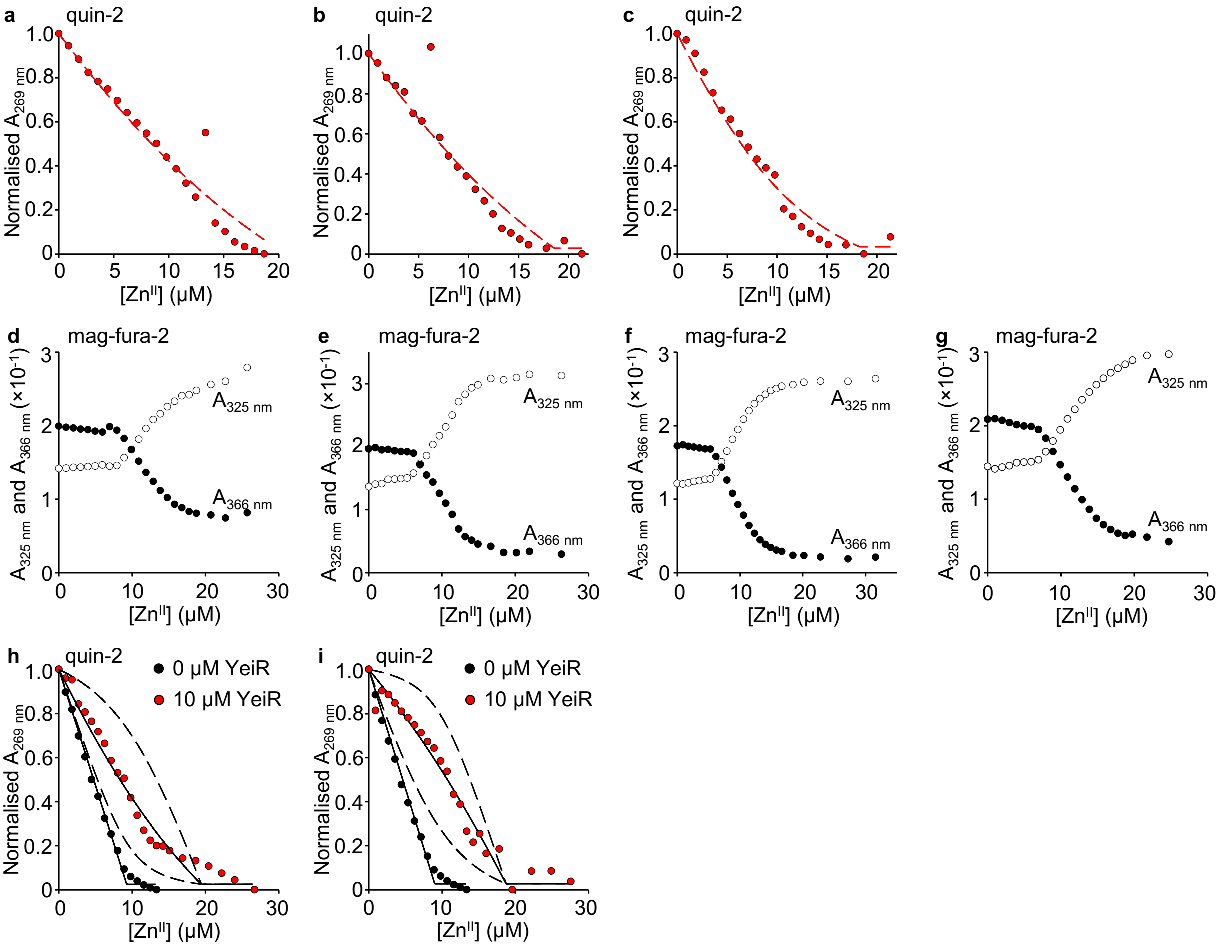

Supplementary Fig. 21. Additional replicates and fittings for Supplementary Fig. 19e-g and 20b,d. a-c Reproduction of data shown in **a** Supplementary Fig. 19e; **b** Supplementary Fig. 19f; and **c** Supplementary Fig. 19g. Red dashed line shows fits to a 1:1 binding model where [Mg^{II}GTP-YeiR] is a fixed rather than a fitted parameter (as in Supplementary Figs. 19e-g). **d-g** Replicates of experiment shown in Supplementary Fig. 20b with **d** [Mf2] = 11 μM, [YeiR] = 10 μM, [GTP] = 100 μM, [Mg^{II}] = 2.7 mM; **e** [Mf2] = 11 μM, [YeiR] = 8.9 μM, [GTP] = 100 μM, [Mg^{II}] = 2.7 mM; **f** [Mf2] = 11 μM, [YeiR] = 8.9 μM, [GTP] = 100 μM, [Mg^{II}] = 2.7 mM; and **g** [Mf2] = 12 μM, [YeiR] = 10 μM, [GTP] = 100 μM, [Mg^{II}] = 2.7 mM. Absorbance at 325 nm and 366 nm shown by open and closed circles, respectively. **h-i** Replicates of experiment shown in Supplementary Fig. 20d with **h** [quin-2] = 9.2 μM, [YeiR] = 10 μM, [GTPγS] = 30 μM, [Mg^{II}] = 2.7 mM, fitted [Mg^{II}GTPγS-YeiR] = 10 μM; and **i** [quin-2] = 9.0 μM, [YeiR] = 10 μM, [GTPγS] = 30 μM, [Mg^{II}] = 2.7 mM, fitted [Mg^{II}GTPγS-YeiR] = 9.7 μM. Titrations in the absence and presence of YeiR are shown in black and red, respectively.

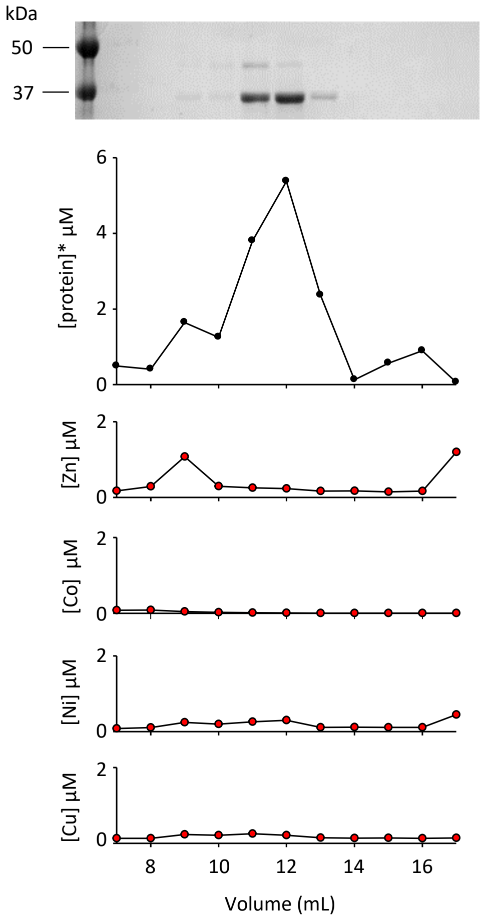

Supplementary Fig. 22. Size exclusion chromatography of YeiR shows co-purification of (trace amounts of) Zn^{II}. YeiR, overexpressed in *E. coli*, was purified from crude cell lysates in two steps using a HisTrap column followed by size exclusion chromatography (Superdex 75 10/300 GL, 24 mL, GE Life Sciences), crucially excluding any intermediate incubation with chelator (EDTA, 10 mM) as normally used to prepare apo-YeiR. Fractions were analysed by SDS-PAGE and $A_{280\ nm}$ for protein (*[protein] is based on $A_{280\ nm}$ using the extinction coefficient of YeiR, shown in black) and for metals (shown in red) by ICP-MS (n = 1). Zn^{II} was the only metal appreciably detected in YeiR-containing fractions: Zn^{II} was detected in a smaller protein peak (fraction 9) eluted slightly earlier than the bulk of YeiR (fractions 11-12). It is formally possible that fraction 9 corresponds to a proportion of YeiR that has retained both Mg^{II}GTP and Zn^{II} during purification. Notably, metals are often lost or exchanged during purification procedures making it notoriously challenging to determine *in vivo* metal occupancies post-extraction (hence the need for a metalation calculator). Metal loss or replacement is especially problematic for weaker metal-protein interactions: For example, the *Salmonella* cobalt chaperone, CbiK, was shown to lose its metal during purification[11]. The Zn^{II} affinity of Mg^{II}GTP-YeiR is significantly tighter ($K_{Zn(II)}$ = 3.0 (±1.2) × 10^{-12} M, Supplementary Table 5) than the Co^{II} affinity of CbiK ($K_{Co(II)}$ = 1.4 (±0.1) × 10^{-8} M, ref.[11]), but YeiR is not an ideal choice of protein for preserving *in vivo* metalation, since GTP hydrolysis and/or dissociation may occur during a multi-step purification from crude cell lysates. Additionally, the YeiR purification protocol involved exposure to Ni^{II}-containing HisTrap resin and 50 mM imidazole. Analysis of the metal-dependent products of a biochemical pathway (such as CobW-dependent B_{12} biosynthesis) provides an alternative readout of *in vivo* metal occupancy.

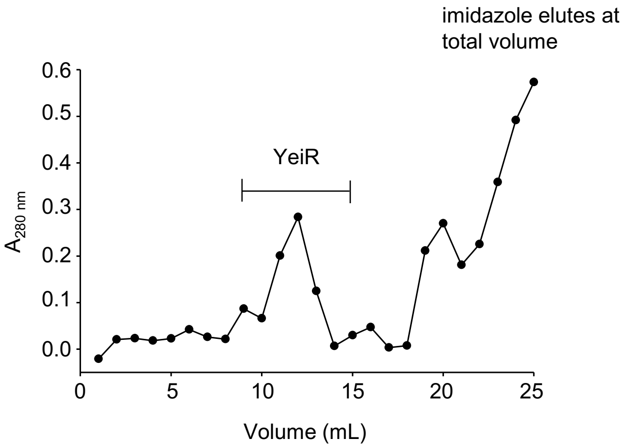

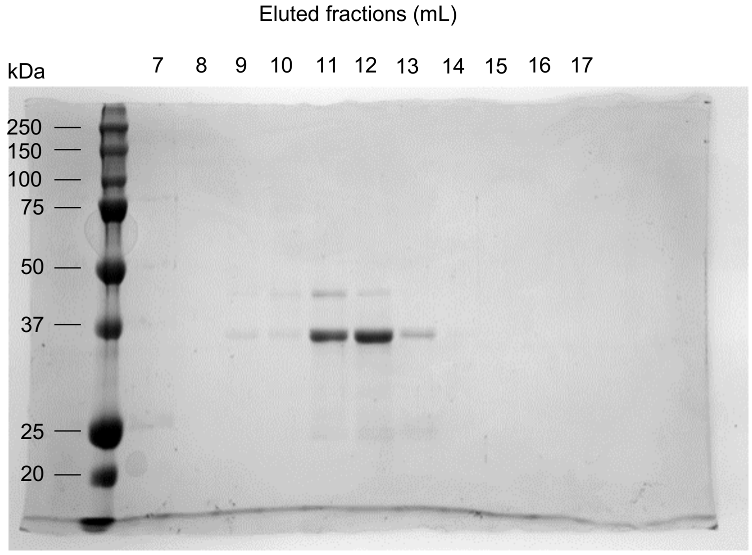

Supplementary Fig. 23. Source figures for Supplementary Fig. 22. A_{280} analysis of YeiR elution fractions from SEC (1-25 mL) and full gel image for SDS-PAGE analysis of protein elution peak (fractions 7-17) with size marker indications (kDa).

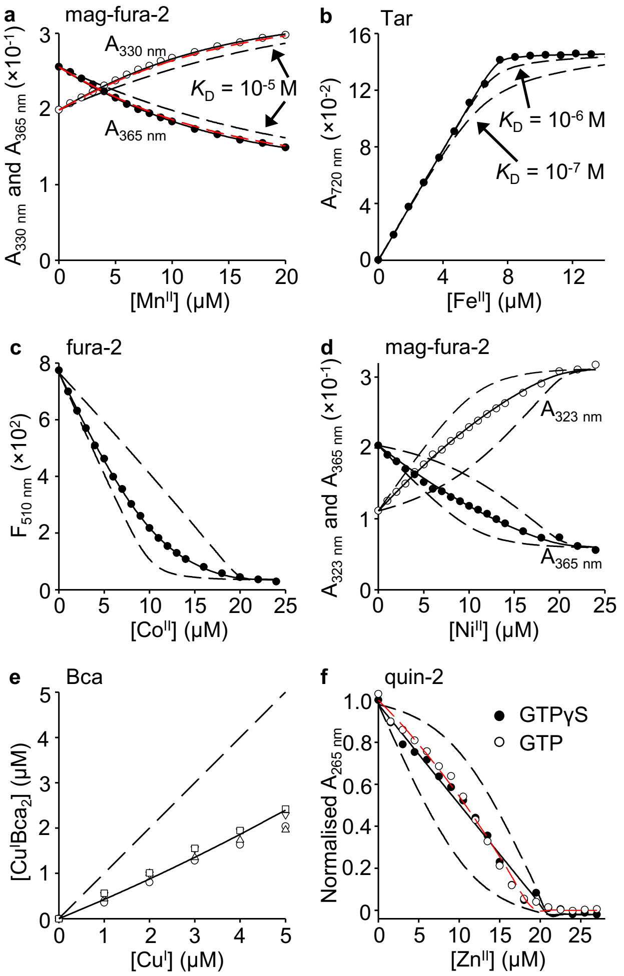

Supplementary Fig. 24. Metal affinities of Mg^{II}GTPγS-YjiA and Mg^{II}GTP-YjiA. a. Representative (n = 4) absorbance change of Mf2 (11 µM) at 330 nm (open circles) and 365 nm (closed circles) upon titration with Mn^{II} in the presence of YjiA (10 µM), GTPγS (100 µM) and Mg^{II} (2.7 mM). Solid black lines represent a fit to a 1:1 binding model. Dashed black lines show simulated responses for YjiA-Mg^{II}GTPγS $K_{Mn(II)} = 10^{-5}$ M and dashed red line for $K_{Mn(II)} = 10^{-4}$ M, providing limiting $K_{Mn(II)} \geq 10^{-4}$ M. **b.** Representative (n = 4) absorbance change upon Fe^{II}-titration into a mixture of Tar (15 µM), Mg^{II} (2.7 mM) and GTPγS (100 µM) in the presence of YjiA (10 µM). Dashed lines show simulated responses for specified $K_{Fe(II)}$ of Mg^{II}GTPγS-YjiA, providing limiting $K_{Fe(II)} \geq 10^{-6}$ M. **c.** Representative (n = 4) fluorescence emission quenching of fura-2 (10 µM) in the presence of YjiA (9.9 µM), GTPγS (100 µM) and Mg^{II} (2.7 mM) upon titration with Co^{II}. Solid line represents a fit to a 1:1 binding model with the dashed lines representing simulated fits to affinities 10-fold tighter and weaker than the fitted value. **d.** Representative (n = 3) absorbance change of Mf2 (11 µM) at 323 nm (open circles) and 365 nm (closed circles) upon titration with Ni^{II} in the presence of YjiA (10 µM), GTPγS (100 µM) and $MgCl_2$ (2.7 mM). Solid lines represent a fit to a 1:1 binding model with the dashed lines representing simulated fits to affinities 10-fold tighter and weaker than the fitted value. **e.** Concentration of $Cu^{I}Bca_2$ formed upon titration of Cu^{I} into a solution of Bca (250 µM), YjiA (11 µM), GTPγS (100 µM) and Mg^{II} (2.7 mM). Absorbance at 562 nm was measured at equilibrium and converted to [$Cu^{I}Bca_2$]. Different shapes represent different experiments (n = 4). Dashed line represents expected [$Cu^{I}Bca_2$] in the absence of Mg^{II}GTPγS-YjiA and solid line is a simulation of the Mg^{II}GTPγS-YjiA $K_{Cu(I)}$ calculated via equation (9) (see methods). Full titrations shown in Supplementary Fig. 25l. **f.** Representative (n = 3, for both experiments) normalised absorbance change of quin-2 (11 µM) upon titration with Zn^{II} in the presence of YjiA (10 µM), GTPγS (100 µM) and Mg^{II} (2.7 mM) (closed circles) or quin-2 (8.5 µM) in the presence of YjiA (10 µM), GTP (100

μM) and Mg^{II} (2.7 mM) (open circles). Solid black line represents a fit to a 1:1 binding model for the GTPγS data, dashed red line represents a fit to a 1:1 binding model for the GTP data. Dashed black lines represent simulated fits to affinities 10-fold tighter and weaker than the fitted value for the GTPγS data. Replicate experiments for **a-d, f** and full data for **e** shown in Supplementary Fig. 25.

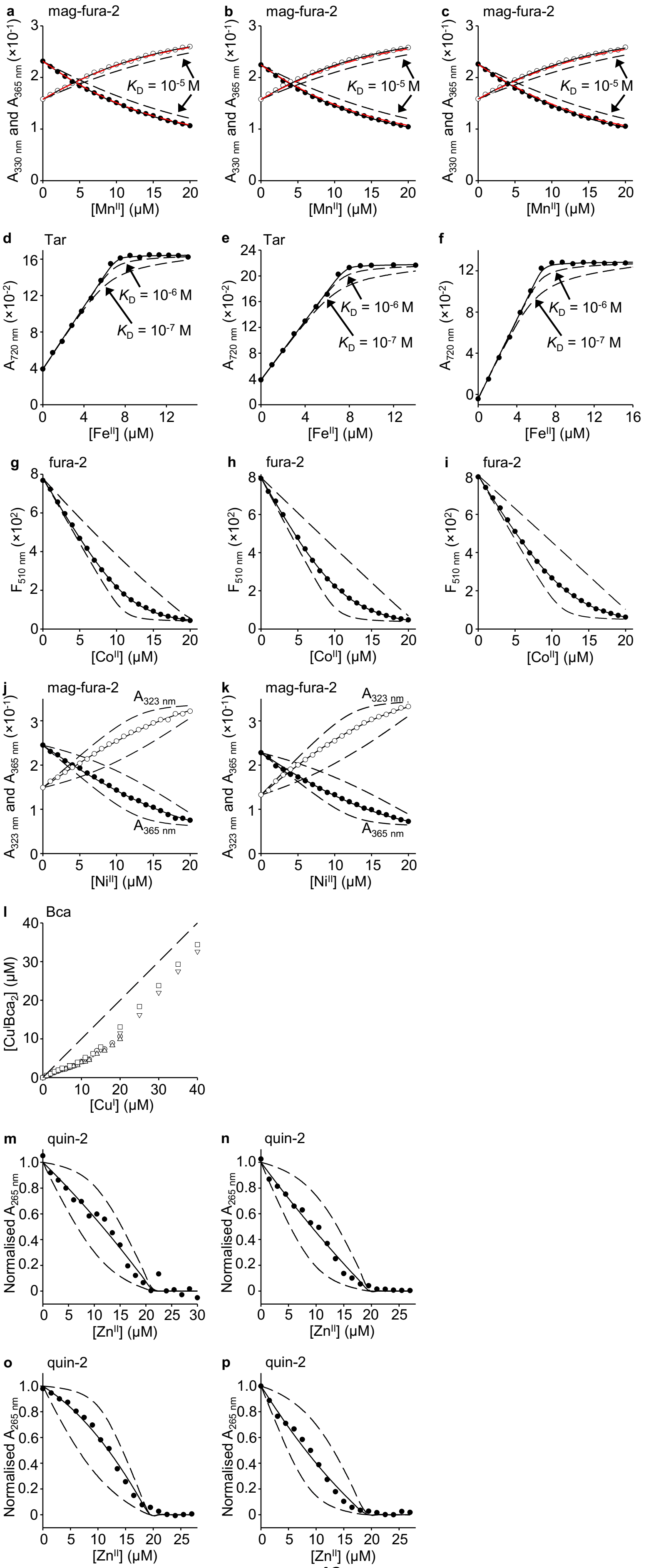

a mag-fura-2
b mag-fura-2
c mag-fura-2
$A_{330\,nm}$ and $A_{365\,nm}$ (×10^{-1})
K_D = 10^{-5} M
[Mn^{II}] (μM)
d Tar
e Tar
f
$A_{720\,nm}$ (×10^{-2})
K_D = 10^{-6} M
K_D = 10^{-7} M
[Fe^{II}] (μM)
g fura-2
h fura-2
i fura-2
$F_{510\,nm}$ (×10^2)
[Co^{II}] (μM)
j mag-fura-2
k mag-fura-2
$A_{323\,nm}$ and $A_{365\,nm}$ (×10^{-1})
$A_{323\,nm}$
$A_{365\,nm}$
[Ni^{II}] (μM)
l Bca
[Cu^{I}Bca$_2$] (μM)
[Cu^I] (μM)
m quin-2
n quin-2
o quin-2
p quin-2
Normalised $A_{265\,nm}$
[Zn^{II}] (μM)

Supplementary Fig. 25. Replicate data for Supplementary Fig. 24. a-c Replicates of experiment shown in Supplementary Fig. 24a with **a** [Mf2] = 12 μM, [YjiA] = 9.7 μM, [GTPγS] = 100 μM, [Mg^{II}] = 2.7 mM; **b** [Mf2] =12 μM, [YjiA] = 11 μM, [GTPγS] = 100 μM, [Mg^{II}] = 2.7 mM; and **c** [Mf2] = 13 μM, [YjiA] = 11 μM, [GTPγS] = 100 μM, [Mg^{II}] = 2.7 mM. Absorbance at 330 nm and 365 nm shown by open and closed circles, respectively. Simulated $K_{Mn(II)} = 10^{-4}$ M (dashed red line) overlays data fit (solid black line). **d-f** Replicates of experiment shown in Supplementary Fig. 24b with **d** [Tar] = 15 μM, [YjiA] = 9.1 μM, [GTPγS] = 100 μM, [Mg^{II}] = 2.7 mM; **e** [Tar] = 18 μM, [YjiA] = 10 μM, [GTPγS] = 100 μM, [Mg^{II}] = 2.7 mM; and **f** [Tar] = 16 μM, [YjiA] = 11 μM, [GTPγS] = 100 μM, [Mg^{II}] = 2.7 mM. **g-i** Replicates of experiment shown in Supplementary Fig. 24c with **g** [fura-2] = 11 μM, [YjiA] = 9.2 μM, [GTPγS] = 100 μM, [Mg^{II}] = 2.7 mM; **h** [fura-2] = 10 μM, [YjiA] = 11 μM, [GTPγS] = 100 μM, [Mg^{II}] = 2.7 mM; and **i** [fura-2] = 11 μM, [YjiA] = 10 μM, [GTPγS] = 100 μM, [Mg^{II}] = 2.7 mM. **j-k** Replicates of experiment shown in Supplementary Fig. 24d with **j** [Mf2] = 13 μM, [YjiA] = 9.9 μM, [GTPγS] = 100 μM, [Mg^{II}] = 2.7 mM; and **k** [Mf2] = 12 μM, [YjiA] = 10 μM, [GTPγS] = 100 μM, [Mg^{II}] = 2.7 mM. Absorbance at 323 nm and 365 nm shown by open and closed circles, respectively. **l.** Full data sets for experiments shown in Supplementary Fig. 24e. Different shapes represent different experiments. **m-n** Replicates of experiment shown in Supplementary Fig. 24f (GTPγS only) with **m** [quin-2] = 11 μM, [YjiA] = 11 μM, [GTPγS] = 100 μM, [Mg^{II}] = 2.7 mM; and **n** [quin-2] = 9.1 μM, [YjiA] = 11 μM, [GTPγS] = 100 μM, [Mg^{II}] = 2.7 mM. **o-p** Replicates of experiment shown in Supplementary Fig. 24f (GTP only) with **o** [quin-2] = 8.7 μM, [YjiA] = 11 μM, [GTP] = 100 μM, [Mg^{II}] = 2.7 mM; and **p** [quin-2] = 8.6 μM, [YjiA] = 10 μM, [GTP] = 100 μM, [Mg^{II}] = 2.7 mM.

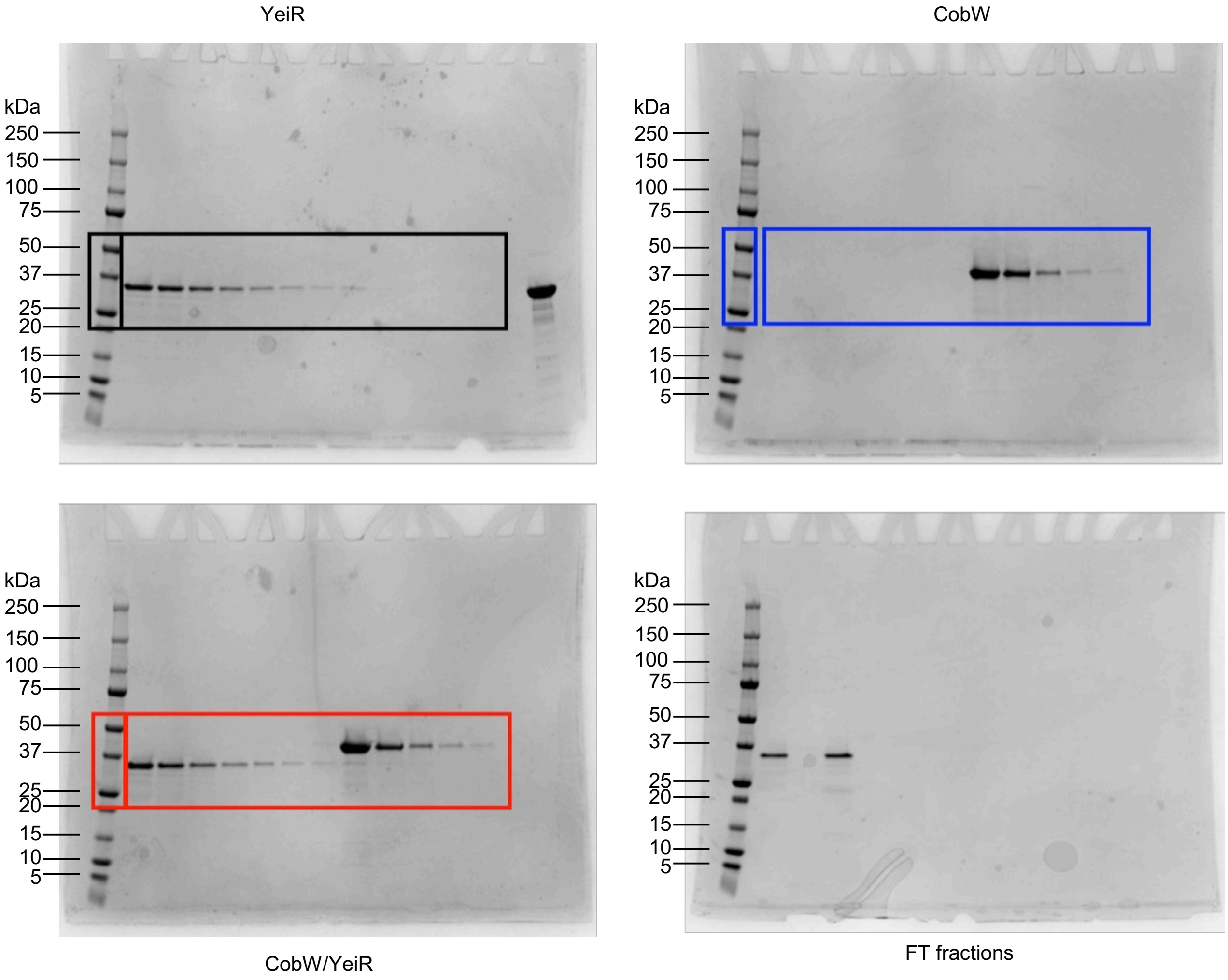

Supplementary Fig. 26. Source figures for Fig. 7. Full gel images for Fig. 7, plus flow through fractions from anion exchange of YeiR, CobW and YeiR/CobW mixtures (lanes 1-3, respectively). Boxes define the tracks visible in Fig. 7 for YeiR (black), CobW (blue) and YeiR/CobW (red).

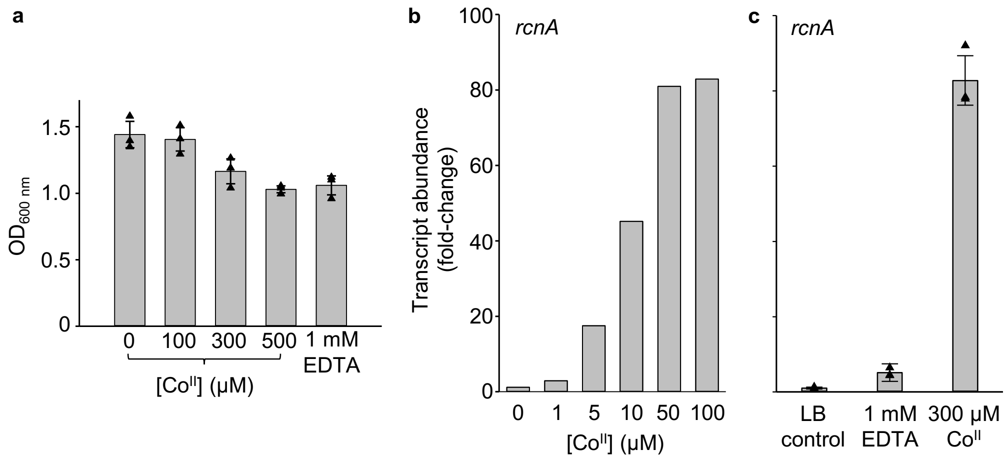

Supplementary Fig. 27. Calibration of maximum and minimum *rcnA* responses. a $OD_{600\ nm}$ of *E. coli** cultures following 4h exposure to Co^{II} or EDTA (added when $OD_{600\ nm} \sim 0.2$). 300 μM Co^{II} and 1 mM EDTA moderately inhibited growth (by 19% and 26%, respectively) relative to untreated control, and were selected as boundary conditions for *rcnA* calibration. **b-c** Transcript abundance (relative to untreated control condition, see Methods) of *rcnA* following 1h exposure of B_{12}-producing *E. coli** to **b** non-inhibitory Co^{II} concentations (0 – 100 μM) (n = 1), and **c** boundary conditions determined from **a** (1 mM EDTA or 300 μM Co^{II}). Addition of 1 mM EDTA did not lead to further repression of *rcnA* expression but instead produced a slight increase in *rcnA* transcript abundance relative to untreated LB; thus the calibrated minimum and maximum were determined from cells grown in untreated LB and 300 μM Co^{II}, respectively. Data in **a** and **c** are the mean ± SD of n = 3 biologically independent replicates; triangles represent individual experiments (some data points overlap, experimental values are available in Source Data files). Note that untreated control sample shown in **c** is replicated data from Fig. 8b (these samples were cultured, and RNA collected, simultaneously, as part of a single experiment).

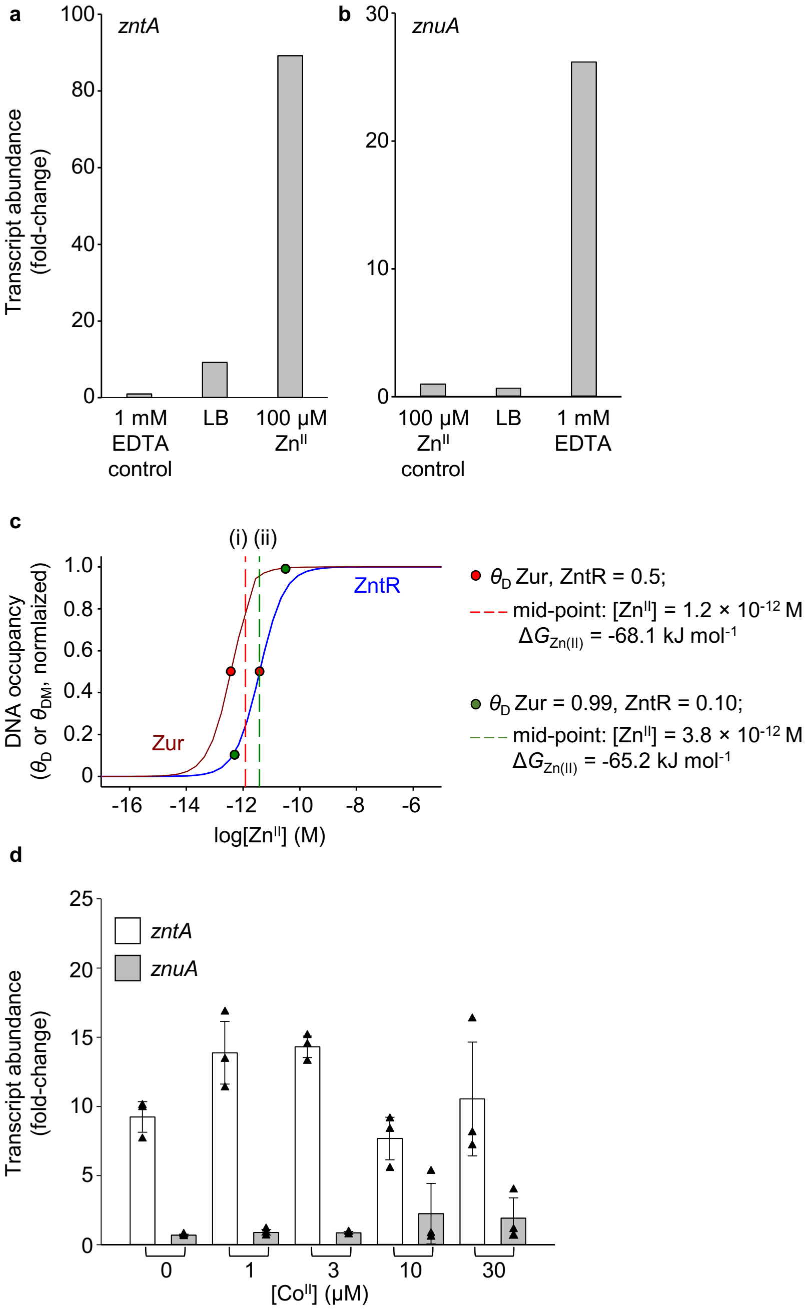

Supplementary Fig. 28. Estimation of Zn^{II} availability within *E. coli cells in defined growth conditions (conditional cells). a** Transcript abundance of *zntA* (regulated by activator ZntR) following 1h exposure of *E. coli** to EDTA (1 mM), Zn^{II} (100 μM) or LB only (n = 1); fold-changes were calculated relative to the EDTA-treated sample (control condition), in which minimum *zntA* expression was observed. The calculated ZntR response in LB media (equation (12)) was θ_D = 0.10. **b** Transcript abundance of *znuA* (regulated by co-repressor Zur) following 1h exposure of *E. coli** to EDTA (1 mM), Zn^{II} (100 μM) or LB only (n = 1); fold-changes were calculated relative to the Zn^{II}-treated sample (control condition), in which minimum *znuA* expression is expected. *znuA* abundance was comparable in LB and Zn^{II}-treated samples, thus we inferred $\theta_D \geq 0.99$ in LB media. Zur may not be fully de-repressed by addition of 1 mM EDTA, as achieving a maximal Zur-response is known to be difficult in cultured cells[15]. **c** The intracellular available [Zn^{II}] corresponding to half of each sensors response (red circles) differ and we previously reasoned that the intracellular available [Zn^{II}] in an idealised cell (*ie* neither Zn^{II}-deficiency nor -excess) will be mid-

way between these values ('i', dotted red trace)[11]. The intracellular [Zn^{II}] corresponding to estimated sensor responses in LB media (from **a** and **b**; green circles) and the [Zn^{II}] mid-way between these values ('ii', dotted green trace) are also shown. Since (i) and (ii) differed only marginally, for the calculations in Fig. 9a we assumed the intracellular available [Zn^{II}] to be that of an idealised cell ($\Delta G_{Zn(II)}$ = -68.1 kJ mol^{-1}). **d** Transcript abundance of *zntA* and *znuA* within *E. coli** following 1h treatment with Co^{II}, measured by qPCR. The data show that intracellular Zn^{II} availability remains constant (within experimental error) in all samples, and is consistent with that estimated in unsupplemented LB (from **a** and **b**). Data are the mean ± SD of n = 3 biologically independent replicates (fold-changes are calculated relative to control conditions specified in **a** and **b**, where n = 1 for control conditions)**.** Triangles represent individual experiments (some data points overlap, experimental values are available in Source Data files).

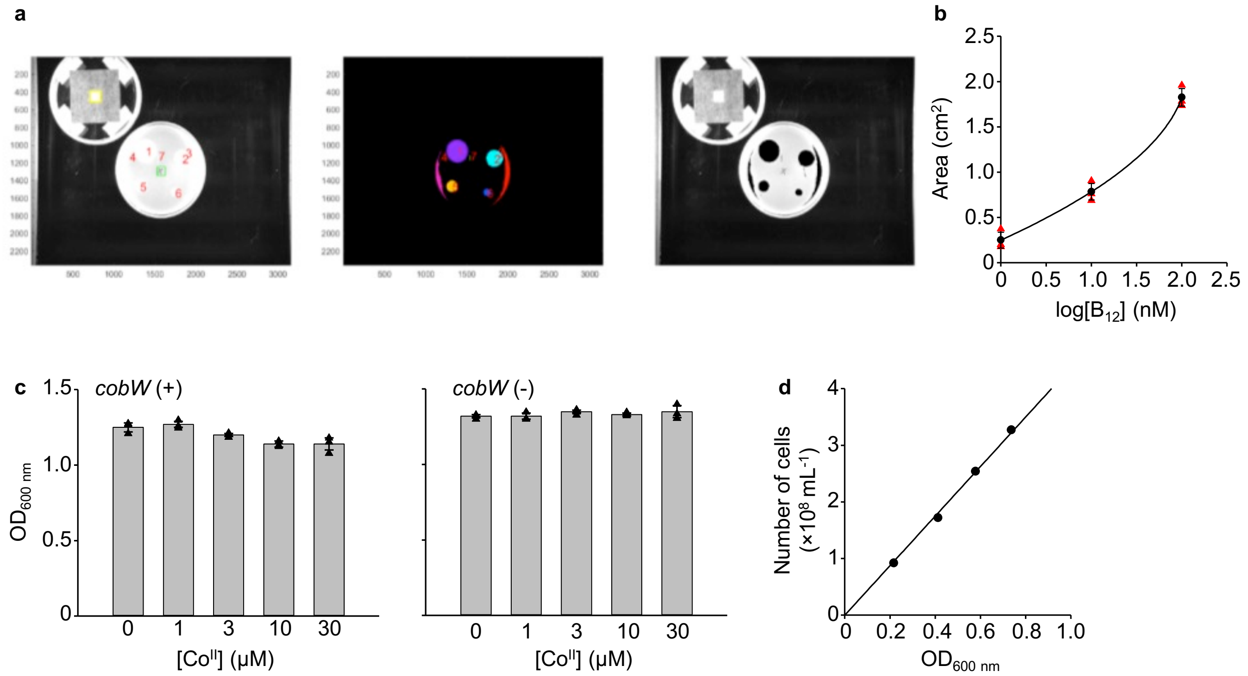

Supplementary Fig. 29. Quantification of vitamin B_{12} in *E. coli*.* a Source figure for Fig. 9b (inset): output of automated analysis of AR2680 growth areas from representative (n = 3) bioassay plate of B_{12} calibration standards (1-1000 nM) imaged together with a 1.0 cm^2 reference area (provided as an example .tiff file in Supplementary Data 2). **b** Calibration curve correlating B_{12} concentration and growth area from data in **a** (1 – 100 nM only, since experimental samples contained ≤ 100 nM B_{12}). Data (black) show average ± SD from n = 3 experiments, red triangles are individual experiments (some data points overlap, experimental values are available in Source Data files). **c** Final $OD_{600\ nm}$ of *E. coli** cultures when harvested for B_{12} analysis. Data are the mean ± SD of n = 3 biologically independent replicates. Triangles represent individual experiments (some data points overlap, experimental values are available in Source Data files). **d** Representative calibration curve relating number of cells with $OD_{600\ nm}$ for *E. coli**. A correlation factor of 4.4 ± 0.1 × 10^8 cells mL^{-1} $OD_{600\ nm}^{-1}$ (mean ± SD of n = 3 biological replicates) was determined.

a

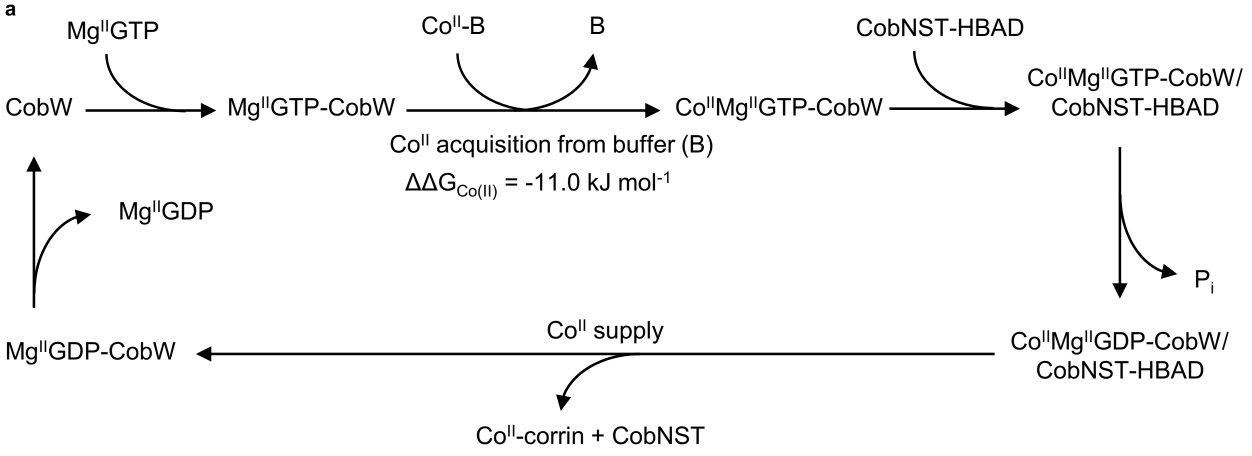

b

$Co^{II}Mg^{II}GDP$-CobW + B ⟶ $Mg^{II}GDP$-CobW + Co^{II}-B ΔG = -9.1 kJ mol^{-1}
(= - $\Delta\Delta G_{Co(II)}$ for $Mg^{II}GDP$-CobW)

Supplementary Fig. 30. Proposed mechanism of CobW. a Binding of $Mg^{II}GTP$ enables CobW to acquire Co^{II} from intracellular buffer ligands (B) and GTP hydrolysis will trigger Co^{II} release from CobW (since the reaction in **b** is thermodynamically favourable). The intrinsic GTPase activity of CobW is slow (see Fig. 2f,g and Supplementary Fig. 6), as observed for other COG0523 proteins[16-19]. Giedroc and co-workers hypothesised that interactions with partner proteins may stimulate GTP hydrolysis in COG0523 proteins[19]. Likewise, we speculate that CobNST could act as a guanine nucleotide activating protein (GAP) enabling Co^{II} release to be targeted to the cobaltocheletase. Release of Co^{II} mediated by CobNST acting as a guanine nucleotide exchange-factor (GEF) is also formally possible[20]. By analogy to ZTP-ZagA[21], GTP-binding (and subsequent metal-acquisition) by CobW could promote interaction with CobNST and contribute to the reaction cycle shown in **a**. Dissociation of $Mg^{II}GDP$ (or nucleotide exchange), resets the reaction cycle with GTPases thought to be saturated with nucleotide (either GTP or GDP) inside cells[22].

```
YeiR      -----MTKTNLITGFLGSGKTTSILHLLAHKDPAEKWAVLVNEFGEVGIDGALLADSG--  53
CobW      MSDLTKIPVTVITGFLGAGKTTLIRHLMANP-EGRKLAVLVNEFGTVGVDGEILRQCADE  59
YjiA      ---MTPIAVTLLTGFLGAGKTTLLRHILNEQ-HGFKIAVIENEFGEVSVDDQLIGDRA--  54

YeiR      ----ALLKEIPGGCMCCVNGLPMQVGLNTLLR-----QGKPDRLLIEPTGLGHPKQILDL  104
CobW      NCPDENIVELANGCICCTVADEFIPTIEALMAR----PVRPDHILIETSGLALPKPLLKA  115
YjiA      ----TQIKTLTNGCICCTRSNELEDALLDLLDSRDRGDIAFDRLVIECTGMADPGPIIQT  110

YeiR      LTA-PVYEPWIDLRATLCILDPR---------------------LLLDQQSVANENFRDQ  142
CobW      FDW-PAIRSKITVDGVIAVADAEAVAAGRFAPDVAAVDAQRQADDIIDHETPLSEVFEDQ  174
YjiA      FFSHDVLCERYLLDGVIALVDAVHANE--------------QM--------NQFTIAQSQ  148

YeiR      LASADIIIANKTDRATAQSDAALQQWWR-QYGGDRQLIHAEHGQIDGKLLDLPRQNLAEL  201
CobW      IACADIVLLSKADLAGAEGLATARALIEAELPRKLPILPLTEGVIDPKVILGLGA-AA-E  232
YjiA      IGYADRILLTKTDVAGDSEKLRERL---ARINARAPVYTVVHGDIDLSQLFNTSGFMLEE  205

YeiR      PASAAHSHTHASKKGLAALNLPAQQRWRRSLNSRQGHQA------CGWIFDADTVFDTIG  255
CobW      ------------------DDLAARPSHHDDHDDHEHDDFDTVVIELPEIADPAALVAAI-  273
YjiA      ------------------NVLASQPRFHFIAD--KQNDVSSIVVELDYPVDISEVSRVME  245

YeiR      LLEWARLAPVGRVKGVMRIQEGLVRINRQGDDLHIETQSVAP-----PDSRVELIS-NTE  309
CobW      -ERLAREQNILRVKGHIAVAGKPMRLLVQAVGERVRHQYDRPWGTEARRSALVVIAEHHD  332
YjiA      NLLLESADKLLRYKGMLWIDGEPNRLLFQGVQRLYSADWDRPWGDETPHSTLVFIGIQLP  305

YeiR      TDWNTLQTALLKLRLATHA   328
CobW      VDEAAIRAVLLGGVAA---   348
YjiA      EDE--IRAAFVGLRK----   318
```

Walker A/B
Guanine Recognition
CxCC (conserved in all COG0523)
Cysteines (conserved in CobW only)

Supplementary Fig. 31. Alignment of COG0523 proteins investigated in this work. Multiple sequence alignment of CobW (*R. capsulatus*), YeiR (*Salmonella*) and YjiA (*Salmonella*). Walker A (GxxGxGK[S/T] in yellow), Walker B (hhhExxG in yellow) and guanine recognition (N[T/S]xKD in cyan) sequence motifs are conserved in G3E GTPases[23]. The putative metal-binding motif (CxCC in magenta) is conserved in the COG0523 subfamily[24]. Two additional cysteines in *R. capsulatus* CobW (C_{56} and C_{61} in green) are conserved in all examined CobWs (from *Rhodobacter capsulatus, Rhodobacter spheroids, Brucella melitensis, Mesorhizobium loti, Pseudomonas fluorescens, Rhizobium leguminosarum and Sinorhizobium meliloti*) but are missing from predicted Zn^{II}-binding COG0523 proteins (*Salmonella* YeiR, *Salmonella* YjiA, *Bacilius subtilis* ZagA[21], *Acenitobacter baumanii* ZigA[25], *Staphylococcus* ZigA[19]).

Supplementary Note 1. Derivation of equations for calculating the *in vivo* metal occupancy of a protein (equations 1-4 in main text).

Derivation of equations 1 and 2 (in main text)

The following equilibrium describes the dissociation of a complex of metal (M) and protein (P)

$$\mathrm{MP} \rightleftharpoons \mathrm{M} + \mathrm{P}$$

The equilibrium constant for the dissociation reaction is

$$K_{\mathrm{MP}} = \frac{[\mathrm{M}][\mathrm{P}]}{[\mathrm{MP}]} \qquad (1)$$

The standard free energy corresponding to the formation of the metal-protein complex is given by

$$\Delta G_{\mathrm{MP}} = -RT\ln\left(\frac{1}{K_{\mathrm{MP}}}\right) = RT\ln(K_{\mathrm{MP}}) \qquad (2)$$

where R (gas constant) = 8.314 × 10^{-3} kJ K^{-1} mol^{-1} and T (temperature) = 298.15 K.

Equation (2) can be rearranged to

$$K_{\mathrm{MP}} = \mathrm{e}^{\frac{\Delta G_{\mathrm{MP}}}{RT}} \qquad (3)$$

Similar equations can be written for the dissociation of a complex between a metal and any hypothetical ligand. Intracellular available ΔG_{M}, defined as the free energy required for a hypothetical ligand to become 50% metalated from available intracellular metal (that is, [MP] = [P]), can be derived from (1) and (2), considering that [M] is the concentration of intracellular available metal

$$\Delta G_{\mathrm{M}} = RT\ln([\mathrm{M}]) \qquad (4)$$

Equation (4) can thus be rearranged to express [M]

$$[\mathrm{M}] = \mathrm{e}^{\frac{\Delta G_{\mathrm{M}}}{RT}} \qquad (5)$$

The difference between the free energy for protein metalation, ΔG_{MP}, and intracellular available ΔG_{M} is defined as

$$\Delta\Delta G_{\mathrm{M}} = \Delta G_{\mathrm{MP}} - \Delta G_{\mathrm{M}} \qquad (6)$$

The fractional occupancy of a protein with metal is defined as

$$\text{Fractional occupancy (\%)} = 100 \times \frac{[\mathrm{MP}]}{[\mathrm{P}]_{\mathrm{tot}}} \qquad (7)$$

where

$$[\mathrm{P}]_{\mathrm{tot}} = [\mathrm{P}] + [\mathrm{MP}] \qquad (8)$$

is the mass balance equation for the protein.

After combining (7) and (8), equation (1) can be used to give

$$\text{Fractional occupancy (\%)} = 100 \times \frac{\frac{[\text{M}][\text{P}]}{K_{\text{MP}}}}{\left(\text{P} + \frac{[\text{M}][\text{P}]}{K_{\text{MP}}}\right)}$$

$$\Rightarrow \text{Fractional occupancy (\%)} = 100 \times \frac{\frac{[\text{M}]}{K_{\text{MP}}}}{\left(1 + \frac{[\text{M}]}{K_{\text{MP}}}\right)}$$

Substituting (3) and (5), the equation can be written as a function of ΔG_{M} and ΔG_{MP}

$$\text{Fractional occupancy (\%)} = 100 \times \frac{\text{e}^{\frac{\Delta G_{\text{M}} - \Delta G_{\text{MP}}}{RT}}}{\left(1 + \text{e}^{\frac{\Delta G_{\text{M}} - \Delta G_{\text{MP}}}{RT}}\right)}$$

Finally, using equation (6) gives

$$\Rightarrow \text{Fractional occupancy (\%)} = 100 \times \frac{\text{e}^{\frac{-\Delta\Delta G_{\text{M}}}{RT}}}{\left(1 + \text{e}^{\frac{-\Delta\Delta G_{\text{M}}}{RT}}\right)}$$

Derivation of equation 3 (in main text)

Based on exchange constant in Fig. 4a

$$\frac{[\text{Co}^{\text{II}}\text{L}]}{[\text{Zn}^{\text{II}}\text{L}]} \times \frac{[\text{Zn}^{\text{II}}\text{P}]}{[\text{Co}^{\text{II}}\text{P}]} = \frac{K_{\text{Zn}^{\text{II}}\text{L}} K_{\text{Co}^{\text{II}}\text{P}}}{K_{\text{Co}^{\text{II}}\text{L}} K_{\text{Zn}^{\text{II}}\text{P}}}$$

Equation (1) can be used to substitute $K_{\text{Zn}^{\text{II}}\text{L}}$ and $K_{\text{Co}^{\text{II}}\text{L}}$, giving

$$\frac{[\text{Zn}^{\text{II}}\text{P}]}{[\text{Co}^{\text{II}}\text{P}]} = \frac{[\text{Zn}^{\text{II}}\text{L}]}{[\text{Co}^{\text{II}}\text{L}]} \times \frac{[\text{L}][\text{Zn}^{\text{II}}]}{[\text{Zn}^{\text{II}}\text{L}]} \times \frac{[\text{Co}^{\text{II}}\text{L}]}{[\text{Co}^{\text{II}}][\text{L}]} \times \frac{K_{\text{Co}^{\text{II}}\text{P}}}{K_{\text{Zn}^{\text{II}}\text{P}}}$$

$$\Rightarrow \frac{[\text{Zn}^{\text{II}}\text{P}]}{[\text{Co}^{\text{II}}\text{P}]} = \frac{[\text{Zn}^{\text{II}}]}{[\text{Co}^{\text{II}}]} \times \frac{K_{\text{Co}^{\text{II}}\text{P}}}{K_{\text{Zn}^{\text{II}}\text{P}}}$$

The equation can thus be expressed as a function of $\Delta G_{\text{Zn}^{\text{II}}\text{P}}$ and $\Delta G_{\text{Co}^{\text{II}}\text{P}}$ by using (3)

$$\frac{[\text{Zn}^{\text{II}}\text{P}]}{[\text{Co}^{\text{II}}\text{P}]} = \frac{[\text{Zn}^{\text{II}}]}{[\text{Co}^{\text{II}}]} \times \frac{\text{e}^{\frac{\Delta G_{\text{Co}^{\text{II}}\text{P}}}{RT}}}{\text{e}^{\frac{\Delta G_{\text{Zn}^{\text{II}}\text{P}}}{RT}}}$$

Substituting (5) gives

$$\frac{[\text{Zn}^{\text{II}}\text{P}]}{[\text{Co}^{\text{II}}\text{P}]} = \frac{\text{e}^{\frac{\Delta G_{\text{Co}^{\text{II}}\text{P}} - \Delta G_{\text{Co}^{\text{II}}}}{RT}}}{\text{e}^{\frac{\Delta G_{\text{Zn}^{\text{II}}\text{P}} - \Delta G_{\text{Zn}^{\text{II}}}}{RT}}}$$

which can be rearranged using equation (6)

$$\frac{[\text{Zn}^{\text{II}}\text{P}]}{[\text{Co}^{\text{II}}\text{P}]} = \frac{\text{e}^{\frac{\Delta\Delta G_{\text{Co}^{\text{II}}\text{P}}}{RT}}}{\text{e}^{\frac{\Delta\Delta G_{\text{Zn}^{\text{II}}\text{P}}}{RT}}} \quad (9)$$

The mass balance equation for the protein species present in the system in Fig. 4a can be written as

$$[\mathrm{P}]_{\mathrm{tot}} = [\mathrm{Co^{II}P}] + [\mathrm{Zn^{II}P}] + [\mathrm{P}] \quad (10)$$

This can be rearranged by dividing both terms by $[\mathrm{Co^{II}P}]$

$$\Rightarrow \frac{[\mathrm{P}]_{\mathrm{tot}}}{[\mathrm{Co^{II}P}]} = \frac{[\mathrm{Co^{II}P}] + [\mathrm{Zn^{II}P}] + [\mathrm{P}]}{[\mathrm{Co^{II}P}]}$$

$$\Rightarrow \frac{[\mathrm{P}]_{\mathrm{tot}}}{[\mathrm{Co^{II}P}]} = 1 + \frac{[\mathrm{Zn^{II}P}]}{[\mathrm{Co^{II}P}]} + \frac{[\mathrm{P}]}{[\mathrm{Co^{II}P}]}$$

$$\Rightarrow \frac{[\mathrm{Co^{II}P}]}{[\mathrm{P}]_{\mathrm{tot}}} = \frac{1}{1 + \frac{[\mathrm{Zn^{II}P}]}{[\mathrm{Co^{II}P}]} + \frac{[\mathrm{P}]}{[\mathrm{Co^{II}P}]}}$$

Substituting (1) gives

$$\frac{[\mathrm{Co^{II}P}]}{[\mathrm{P}]_{\mathrm{tot}}} = \frac{1}{1 + \frac{[\mathrm{Zn^{II}P}]}{[\mathrm{Co^{II}P}]} + \frac{K_{\mathrm{Co^{II}P}}}{[\mathrm{Co^{II}}]}}$$

Substituting (3) and (5) gives

$$\frac{[\mathrm{Co^{II}P}]}{[\mathrm{P}]_{\mathrm{tot}}} = \frac{1}{1 + \frac{[\mathrm{Zn^{II}P}]}{[\mathrm{Co^{II}P}]} + \frac{\mathrm{e}^{\frac{\Delta G_{\mathrm{Co^{II}P}}}{RT}}}{\mathrm{e}^{\frac{\Delta G_{\mathrm{Co^{II}}}}{RT}}}}$$

which can then be rearranged using (6)

$$\frac{[\mathrm{Co^{II}P}]}{[\mathrm{P}]_{\mathrm{tot}}} = \frac{1}{1 + \frac{[\mathrm{Zn^{II}P}]}{[\mathrm{Co^{II}P}]} + \mathrm{e}^{\frac{\Delta\Delta G_{\mathrm{Co^{II}}}}{RT}}}$$

Finally, substituting (9) and rearranging the equation gives

$$\frac{[\mathrm{Co^{II}P}]}{[\mathrm{P}]_{\mathrm{tot}}} = \frac{1}{1 + \mathrm{e}^{\frac{\Delta\Delta G_{\mathrm{Co^{II}}} - \Delta\Delta G_{\mathrm{Zn^{II}}}}{RT}} + \mathrm{e}^{\frac{\Delta\Delta G_{\mathrm{Co^{II}}}}{RT}}}$$

$$\Rightarrow \frac{[\mathrm{Co^{II}P}]}{[\mathrm{P}]_{\mathrm{tot}}} = \frac{1}{\mathrm{e}^{\frac{\Delta\Delta G_{\mathrm{Co^{II}}}}{RT}}\left(\mathrm{e}^{\frac{-\Delta\Delta G_{\mathrm{Co^{II}}}}{RT}} + \mathrm{e}^{\frac{-\Delta\Delta G_{\mathrm{Zn^{II}}}}{RT}} + 1\right)}$$

$$\Rightarrow \frac{[\mathrm{Co^{II}P}]}{[\mathrm{P}]_{\mathrm{tot}}} = \frac{\mathrm{e}^{\frac{-\Delta\Delta G_{\mathrm{Co^{II}}}}{RT}}}{\left(\mathrm{e}^{\frac{-\Delta\Delta G_{\mathrm{Co^{II}}}}{RT}} + \mathrm{e}^{\frac{-\Delta\Delta G_{\mathrm{Zn^{II}}}}{RT}} + 1\right)}$$

The fractional occupancy of the protein with Co^{II} can thus be calculated using (7)

$$\Rightarrow \text{Fractional occupancy (\%)} = 100 \times \frac{[\mathrm{Co^{II}P}]}{[\mathrm{P}]_{\mathrm{tot}}} = 100 \times \frac{\mathrm{e}^{\frac{-\Delta\Delta G_{\mathrm{Co^{II}}}}{RT}}}{\left(\mathrm{e}^{\frac{-\Delta\Delta G_{\mathrm{Co^{II}}}}{RT}} + \mathrm{e}^{\frac{-\Delta\Delta G_{\mathrm{Zn^{II}}}}{RT}} + 1\right)}$$

Derivation of equation 4 (in main text)

Let us consider a protein which can bind n different metals (namely M_1 to M_n). The mass balance equation for the protein can be written as

$$[\mathrm{P}]_{\mathrm{tot}} = [\mathrm{P}] + [\mathrm{M_1P}] + [\mathrm{M_2P}] + \cdots + [\mathrm{M_nP}] \tag{11}$$

The fractional occupancy of protein with any of the metals (e.g. with metal M_i) can be calculated using the expression

$$\text{Fractional occupancy (\%)} = 100 \times \frac{[\mathrm{M_iP}]}{[\mathrm{P}]_{\mathrm{tot}}}$$

Using (1), this can be rewritten as

$$\text{Fractional occupancy (\%)} = 100 \times \frac{[\mathrm{M_i}][\mathrm{P}]}{K_{\mathrm{M_i}}[\mathrm{P}]_{\mathrm{tot}}}$$

Substituting (11) gives

$$\text{Fractional occupancy (\%)} = 100 \times \frac{[\mathrm{M_i}][\mathrm{P}]}{K_{\mathrm{M_i}}([\mathrm{P}] + [\mathrm{M_1P}] + [\mathrm{M_2P}] + \cdots + [\mathrm{M_nP}])}$$

which can be then rearranged to

$$\text{Fractional occupancy (\%)} = 100 \times \frac{[\mathrm{M_i}]}{K_{\mathrm{M_i}}\left(1 + \frac{[\mathrm{M_1P}]}{[\mathrm{P}]} + \frac{[\mathrm{M_2P}]}{[\mathrm{P}]} + \cdots + \frac{[\mathrm{M_nP}]}{[\mathrm{P}]}\right)}$$

Equation (1) can be used once again at the denominator, giving

$$\text{Fractional occupancy (\%)} = 100 \times \frac{[\mathrm{M_i}]}{K_{\mathrm{M_i}}\left(1 + \frac{[\mathrm{M_1}]}{K_{\mathrm{M_1}}} + \frac{[\mathrm{M_2}]}{K_{\mathrm{M_2}}} + \cdots + \frac{[\mathrm{M_n}]}{K_{\mathrm{M_n}}}\right)}$$

Using equations (3) and (5), the fractional occupancy can be written as a function of intracellular available $\Delta G_{\mathrm{M_i}}s$ and of the free energies of protein-metal complex formation $\Delta G_{\mathrm{M_iP}}s$

$$\text{Fractional occupancy (\%)} = 100 \times \frac{\mathrm{e}^{\frac{\Delta G_{\mathrm{M_i}}}{RT}}}{\mathrm{e}^{\frac{\Delta G_{\mathrm{M_iP}}}{RT}}\left(1 + \frac{\mathrm{e}^{\frac{\Delta G_{\mathrm{M_1}}}{RT}}}{\mathrm{e}^{\frac{\Delta G_{\mathrm{M_1P}}}{RT}}} + \frac{\mathrm{e}^{\frac{\Delta G_{\mathrm{M_2}}}{RT}}}{\mathrm{e}^{\frac{\Delta G_{\mathrm{M_2P}}}{RT}}} + \cdots + \frac{\mathrm{e}^{\frac{\Delta G_{\mathrm{M_n}}}{RT}}}{\mathrm{e}^{\frac{\Delta G_{\mathrm{M_nP}}}{RT}}}\right)}$$

This equation can be rearranged, and substituting (6) gives

$$\text{Fractional occupancy (\%)} = 100 \times \frac{\mathrm{e}^{\frac{\Delta G_{\mathrm{M_i}} - \Delta G_{\mathrm{M_iP}}}{RT}}}{\left(1 + \mathrm{e}^{\frac{\Delta G_{\mathrm{M_1}} - \Delta G_{\mathrm{M_1P}}}{RT}} + \mathrm{e}^{\frac{\Delta G_{\mathrm{M_2}} - \Delta G_{\mathrm{M_2P}}}{RT}} + \cdots + \mathrm{e}^{\frac{\Delta G_{\mathrm{M_n}} - \Delta G_{\mathrm{M_nP}}}{RT}}\right)}$$

$$\Rightarrow \text{Fractional occupancy (\%)} = 100 \times \frac{\mathrm{e}^{\frac{-\Delta\Delta G_{\mathrm{M_i}}}{RT}}}{\left(1 + \mathrm{e}^{\frac{-\Delta\Delta G_{\mathrm{M_1}}}{RT}} + \mathrm{e}^{\frac{-\Delta\Delta G_{\mathrm{M_2}}}{RT}} + \cdots + \mathrm{e}^{\frac{-\Delta\Delta G_{\mathrm{M_n}}}{RT}}\right)}$$

Finally, the equation for the fractional occupancy can be written the more compact form

$$\Rightarrow \text{Fractional occupancy (\%)} = 100 \times \frac{e^{\frac{-\Delta\Delta G_{\mathrm{M_i}}}{RT}}}{\left(1 + \sum_{k=1}^{k=n} e^{\frac{-\Delta\Delta G_{\mathrm{M}_k}}{RT}}\right)}$$

Supplementary References

1 Kwan, C.-Y. & Putney, J. Uptake and intracellular sequestration of divalent cations in resting and methacholine-stimulated mouse lacrimal acinar cells: dissociation by Sr(II) and Ba(II) of agonist-stimulated divalent cation entry from the refilling of the agonist-sensitive intracellular pool. *J. Biol. Chem.* **265**, 678-684 (1990).

2 Xiao, Z. & Wedd, A. G. The challenges of determining metal-protein affinities. *Nat. Prod. Rep.* **27**, 768-789 (2010).

3 Cortes, L., Roberts, B. R., Wedd, A. G. & Xiao, Z. Molecular Aspects of a Robust Assay for Ferroxidase Function of Ceruloplasmin. *Inorganic Chemistry* **56**, 5275-5284 (2017).

4 Xiao, Z. *et al.* Unification of the Copper(I) Binding Affinities of the Metallo-chaperones Atx1, Atox1, and Related Proteins: DETECTION PROBES AND AFFINITY STANDARDS. *Journal of Biological Chemistry* **286**, 11047-11055 (2011).

5 Martell, A. & Smith, R. Critical stability constants: amino acids. Vol. 1. *NY: Springer* (1974).

6 Grynkiewicz, G., Poenie, M. & Tsien, R. Y. A new generation of Ca2+ indicators with greatly improved fluorescence properties. *Journal of Biological Chemistry* **260**, 3440-3450 (1985).

7 Lattanzio, F. A. & Bartschat, D. K. The effect of pH on rate constants, ion selectivity and thermodynamic properties of fluorescent calcium and magnesium indicators. *Biochem. Biophys. Res. Commun.* **177**, 184-191 (1991).

8 Tsien, R. Y. New calcium indicators and buffers with high selectivity against magnesium and protons: design, synthesis, and properties of prototype structures. *Biochemistry* **19**, 2396-2404 (1980).

9 Reyes-Caballero, H., Lee, C. W. & Giedroc, D. P. Mycobacterium tuberculosis NmtR Harbors a Nickel Sensing Site with Parallels to Escherichia coli RcnR. *Biochemistry* **50**, 7941-7952 (2011).

10 Jefferson, J. R., Hunt, J. B. & Ginsburg, A. Characterization of indo-1 and quin-2 as spectroscopic probes for Zn(II)-protein interactions. *Anal. Biochem.* **187**, 328-336 (1990).

11 Osman, D. *et al.* Bacterial sensors define intracellular free energies for correct enzyme metalation. *Nature Chemical Biology* **15**, 241-249 (2019).

12 Kaluarachchi, H. *et al.* Metal selectivity of the *Escherichia coli* nickel metallochaperone, SlyD. *Biochemistry* **50**, 10666-10677 (2011).

13 Young, T. R., Wedd, A. G. & Xiao, Z. Evaluation of Cu(i) binding to the E2 domain of the amyloid precursor protein – a lesson in quantification of metal binding to proteins via ligand competition. *Metallomics* **10**, 108-119 (2018).

14 Gilston, B. A. *et al.* Structural and Mechanistic Basis of Zinc Regulation Across the E. coli Zur Regulon. *PLoS Biol.* **12**, e1001987 (2014).

15 Graham, A. I. *et al.* Severe zinc depletion of *Escherichia coli*: roles for high affinity zinc binding by ZinT, zinc transport and zinc-independent proteins. *J. Biol. Chem.* **284**, 18377-18389 (2009).

16 Blaby-Haas, C. E., Flood, J. A., Crecy-Lagard, V. d. & Zamble, D. B. YeiR: a metal-binding GTPase from *Escherichia coli* involved in metal homeostasis. *Metallomics* **4**, 488-497 (2012).

17 Sydor, A. M. *et al.* Metal binding properties of *Escherichia coli* YjiA, a member of the metal homeostasis-associated COG0523 family of GTPases. *Biochemistry* **52**, 1788-1801 (2013).

18 Gumataotao, N., Lankathilaka, K. P. W., Bennett, B. & Holz, R. C. The iron-type nitrile hydratase activator protein is a GTPase. *Biochem. J.* **474**, 247-258 (2017).

19 Jordan, M. R. *et al.* Mechanistic insights into the metal-dependent activation of Zn(II)-dependent metallochaperones. *Inorg. Chem.* **58**, 13661-13672 (2019).

20 Bos, J. L., Rehmann, H. & Wittinghofer, A. GEFs and GAPs: critical elements in the control of small G proteins. *Cell* **129**, 865-877 (2007).

21 Chandrangsu, P., Huang, X., Gaballa, A. & Helmann, J. D. *Bacillus subtilis* FolE is sustained by the ZagA zinc metallochaperone and the alarmone ZTP under conditions of zinc deficiency. *Mol. Microbiol.* **112**, 751-765 (2019).

22 Bourne, H. R., Sanders, D. A. & McCormick, F. The GTPase superfamily: conserved structure and molecular mechanism. *Nature* **349**, 117-127 (1991).

23 Leipe, D. D., Wolf, Y. I., Koonin, E. V. & Aravind, L. Classification and evolution of P-loop GTPases and related ATPases. *J. Mol. Biol.* **317**, 41-72 (2002).

24 Haas, C. E. *et al.* A subset of the diverse COG0523 family of putative metal chaperones is linked to zinc homeostasis in all kingdoms of life. *BMC Genomics* **10**, 470-490 (2009).

25 Nairn, B. L. *et al.* The response of *Acinetobacter baumannii* to zinc starvation. *Cell Host Microbe* **19**, 826-836 (2016).
