# Supplementary material for: Calculating metalation in cells reveals CobW acquires CoII for vitamin B12 biosynthesis while related proteins prefer ZnII
Source: Nat Commun. 2021 Feb 19;12:1195. doi: 10.1038/s41467-021-21479-8 (PMC7895991; doi:10.1038/s41467-021-21479-8)
Supplement: Supplementary file 3 — Description of Additional Supplementary Files [file 41467_2021_21479_MOESM3_ESM.docx]

**Description of Additional Supplementary Files**

**Supplementary Data 1:**

Metalation calculator for determining in vivo metal occupancies of proteins.

**Supplementary Data 2:**

Example input file for Supplementary Software 1.

**Supplementary Software 1:**

MATLAB code for automated area analysis of bacterial growth

**Supplementary Software 2:**

Dynafit Scripts.
