# Supplementary figures and images for: Calculating metalation in cells reveals CobW acquires CoII for vitamin B12 biosynthesis while related proteins prefer ZnII
Source: Nat Commun. 2021 Feb 19;12:1195. doi: 10.1038/s41467-021-21479-8 (PMC7895991; doi:10.1038/s41467-021-21479-8)

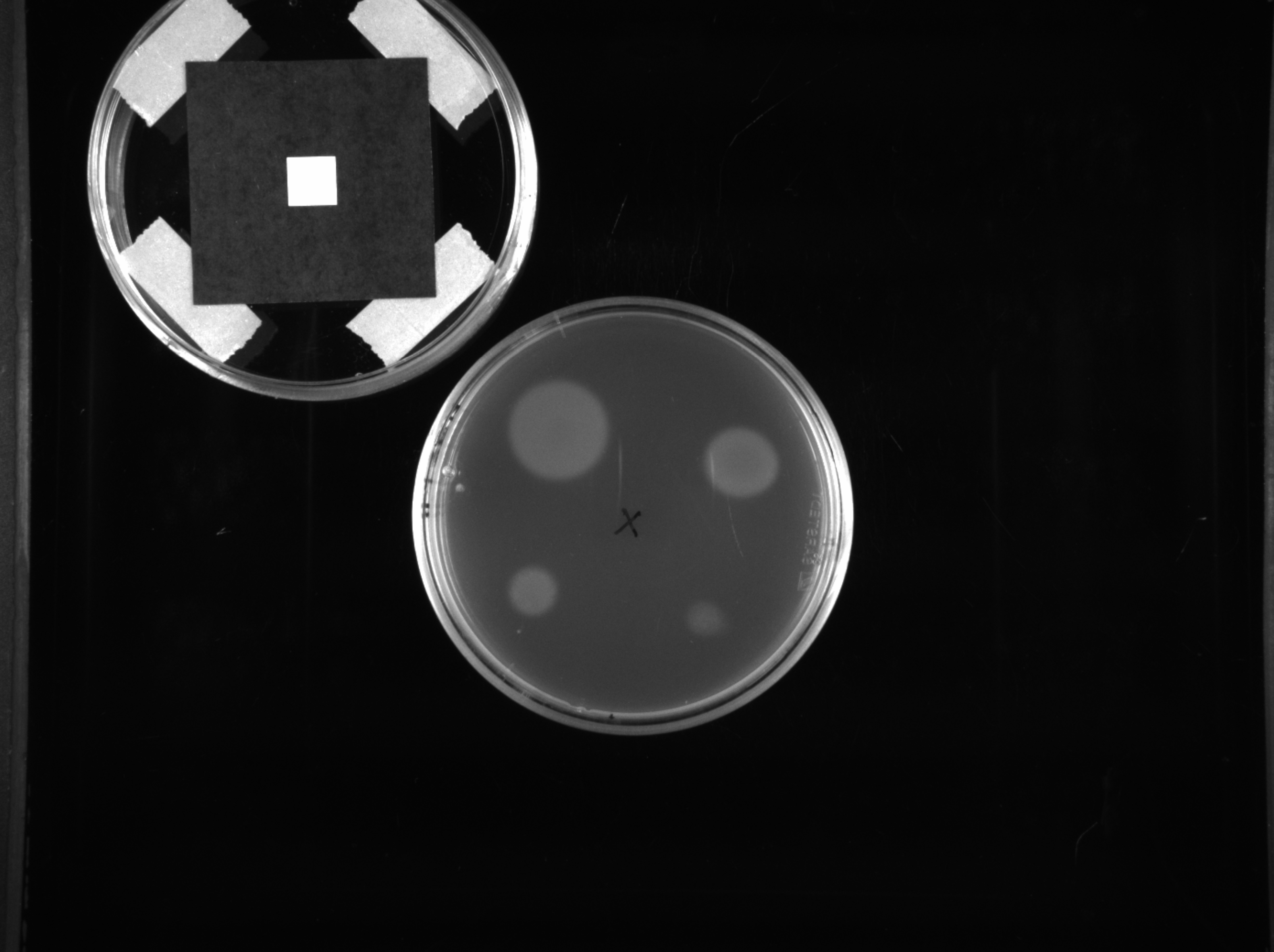

Supplement: Supplementary file 5 — Supplementary Data 2 [file 41467_2021_21479_MOESM5_ESM.zip › Supplementary Data 2.tiff]
