# Supplementary material for: Calculating metalation in cells reveals CobW acquires CoII for vitamin B12 biosynthesis while related proteins prefer ZnII
Source: Nat Commun. 2021 Feb 19;12:1195. doi: 10.1038/s41467-021-21479-8 (PMC7895991; doi:10.1038/s41467-021-21479-8)
Supplement: Supplementary file 6 — Supplementary Software 1 [file 41467_2021_21479_MOESM6_ESM.zip › Supplementary Software 1.docx]

**Supplementary Software 1**

**MATLAB code for automated area analysis of bacterial growth spots**

This file contains:

1. **Description of the MATLAB code**
2. **The MATLAB code and instructions for use**

**1. Description of the MATLAB code**

The objective of the code is to provide a form of image segmentation; identifying spots of bacterial growth on an agar plate and measuring their area.

Digital photographic images form a set of values that correspond to the photons registered at a certain pixel on the camera sensor (alternatively this can be described as the intensity or brightness). Areas of bacterial growth are brighter than the background media, enabling detection and separation of the two, following identification of a suitable threshold value of intensity: The image is split into pixels we identify as either bacteria or media. The act of choosing a threshold value is central to many image segmentation methods.

In addition to areas of bacterial growth and media, there are artefacts present in the image. An artefact is any item that is not of interest e.g. the Petri dish, dirt or scratches on the dish lid/base. The first step in image processing uses a mask to remove any artefacts outside of the Petri dish and the Petri dish edge, leaving only the plate area. Remaining issues include (i) scratches/dirt on Petri dishes, (ii) uneven illumination of the media due to reflected light from the Petri dish edge and (iii) uneven thickness of the agar across the plate. These are difficult to correct in an automated way and should be minimised as much as possible during plate preparation and image capture.

To choose the threshold value, we first need to identify the values of intensity that describe the media. The media is relatively homogenous but will likely contain slight variations in brightness across the plate. Moreover, there will be noise in the image, which arises from photon counting (Poisson noise) and sensor performance. To characterise media intensity, the MATLAB routine calculates the mean ($\mu$) and standard deviation ($\sigma$) of 1000 pixels near the centre of the Petri dish that contain only media (i.e. no culture pixels). The centre of the plate can be marked manually with a black cross before image capture without any effect on this calculation.

The threshold is then set to be a value, $N$, that is a number of standard deviations above the mean value of the media, i.e., $N=\mu+m\sigma$. The best value of $m$ is somewhat subjective, and largely depends on what works in each individual case (there will be variability e.g. culture, media, instrument). There is no objective way to set the threshold value, as there is no discrete edge to the bacterial growth spot. The edge of the growth spot and media are blurred, meaning the intensity curve from spot centre to media decreases smoothly: the $m$ value should be selected following optimisation with different $m$ values. We have found that varying $m$ over a range of 3, e.g. $6\leq m\leq8$ , has negligible influence on the returned value of bacterial growth area. The same $m$ value should be used across a batch of plates which includes at least one calibration plate.

After setting the threshold value, the image is thresholded into bacterial and media pixels. The routine will then find and label each set of contiguous image pixels as defined regions on the plate. Each will be a different bacterial growth spot, but may also include other areas of brightness e.g. from the plate edge; the latter can be ignored. Once labelled, the number of pixels in each region is counted and provides the area of each bacterial growth spot. The area in cm^2^ is determined with reference to a square centimetre included in the image.

**2. The MATLAB code and instructions for use**

**System requirements**

This code has been tested using MATLAB R2019a Version 9.6.0.1072779 running on Windows 10.

**Installation guide**

MATLAB is available to download here: [https://uk.mathworks.com/products/matlab.html](about:blank)

The code operates within MATLAB and therefore there is no additional installation time.

**Demo**

This code calculates areas (in cm^2^) of bacterial growth spots on a Petri dish, prepared as described in Methods. Required input is one or more .tif image files taken on black background: Each .tif file must include both the plate with growth spots to be quantified and a 1cm^2^ reference area in the same image (for scaling), as described above and in Methods. An example image is included in Supplementary Data 2. Expected run time for the example image is < 1 min (on a normal desktop computer).

**Instructions for use**

To run the MATLAB routine:

1. In MATLAB open a new script.
2. Copy the following MATLAB routine (courier new font, below) to the MATLAB editor window and save to the MATLAB folder as “find_blobs_new.m”.
3. Type “find_blobs_new” in the command window and press enter.
4. When prompted, select picture (.tif) file(s) for analysis. Single or multiples .tif files can be selected.
5. When prompted, select points at the bottom left and top right of the 1cm^2^ reference area, then press enter. This will be used to locate the 1cm^2^ reference area.
6. When prompted, select a position in the centre of the Petri dish (where no bacterial growth is observed), then press enter. This will be used to determine the background signal.
7. An annotated image (.png) file will be generated in the current folder window, showing all detected spots and assigning an integer number to each. Outputs in the command window display the area and radius corresponding to each of the detected spots.
8. Repeat steps 5-7 for each image.

**Variable settings (e.g. defining** $\boldsymbol{m}$**)**

Variable settings are editable at the start of the routine and include (i) the diameter of the Petri dish, (ii) the minimum size of bacterial growth spot (blob) and (iii) the detection threshold. For the current analysis, default values for all variable settings were used. To change the detection threshold, use the command “find_blobs_new(‘StandDev’,m)”, where *m* is a number > 0. The default setting (in find_blobs_new.m) is 8. A smaller value of *m* will lower the detection threshold: This allows fainter spots to be detected but may increase false-positive signals, and vice versa (see above description of the MATLAB code).

**MATLAB Routine**

START COPYING:

function find_blobs_new(varargin)

p=inputParser;

%default values

expand_box=20 ; % for finding the cm box - incase user clicks to close to edge

addOptional(p,'expand_box',expand_box,@isnumeric);

PetriDishDiam=1200; % diameter of petri dish

addOptional(p,'PetriDishDiam',PetriDishDiam,@isnumeric);

rad_low=150; % annulus radii for masks

addOptional(p,'rad_low',rad_low,@isnumeric);

mediaBoxLen=50; % half length of media box

addOptional(p,'mediaBoxLen',mediaBoxLen,@isnumeric);

min_blob=500; % minimum size of blobs

addOptional(p,'min_blob',min_blob,@isnumeric);

StandDev=8; % standard deviation for threshold

addOptional(p,'StandDev',StandDev,@isnumeric);

manualSelect='no'; % turn on/off background selection

validSelect = {'no','yes'};

checkSelect = @(x) any(validatestring(x,validSelect));

addOptional(p,'manualSelect',manualSelect,checkSelect);

debugPlot='no'; % turn on/off debugging plots

addOptional(p,'debugPlot',debugPlot,checkSelect);

parse(p,varargin{:});

%Import image file

[FileName,PathName,FilterIndex] = uigetfile('.tif','Select location of tif image file.','multiselect','on');

DateString=strrep(datestr(datetime('now')),':','_');

%------------------------

%Open file to write to

OutputFile=strcat('blob_details_',DateString,'.csv');

fileID = fopen(OutputFile,'w');

%------------------------

if iscellstr(FileName)==1

numberFiles=length(FileName);

else

numberFiles=1;

end

for jj=1:numberFiles

if iscellstr(FileName)==0 ThisFile=FileName; else ThisFile=FileName{jj}; end

Image = imread(strcat(PathName,ThisFile));

ImageSize=size(Image);

% Get rid of transparency channel

if ImageSize(3)== 4 Image(:,:,4) = []; end

GrayImage=rgb2gray(Image);

im_sz=size(GrayImage);

%find 1cm mark

[BoxPos,scale]=find_square(GrayImage,p);

[masks,xPetrieCenter,yPetrieCenter]=create_mask(GrayImage,im_sz,p);

GrayImageMasked=imgaussfilt(double(GrayImage),2);

GrayImageMasked(find(masks == 0))=0;

thresholdLevel=find_threshold(GrayImageMasked,masks,p,xPetrieCenter,yPetrieCenter);

%Threshold image

maxGray=max(GrayImageMasked(:));

BinaryImage = imbinarize(GrayImageMasked/double(maxGray),thresholdLevel);

[labeledImage, numBlobs] = bwlabel(BinaryImage);

[blobSizes,labeledImage]=choose_blobs(numBlobs,labeledImage,p);

[numBlobsLarge,na]=size(blobSizes);

plot_blob_images(GrayImage,labeledImage,BinaryImage,BoxPos,DateString,blobSizes,ThisFile,xPetrieCenter,yPetrieCenter,p);

%------------------------

fprintf(fileID,'\n');

fprintf(fileID,strrep(ThisFile,'.tif',''));

fprintf(fileID,'\n');

fprintf(fileID,'%s,%s,%s,%s\n','Blob Number','Area (Pixels)','Area (cm2)','Radius (cm)');

for i=1:numBlobsLarge

area=blobSizes(i,1)/scale/scale;

radius=(area/pi)^0.5;

fprintf('%d has area %4.3f cm2 and radius cm %4.3f.\n',i,area,radius);

fprintf(fileID,'%d, %d, %4.3f, %4.3f\n',i,blobSizes(i,1),area,radius);

end

fprintf(fileID,'\n');

%------------------------

end

% close text file

fclose(fileID);

end

function [BoxPos,scale]=find_square(GrayImage,p)

fig = figure('position', [0, 0, 1000, 1000]);

imshow(GrayImage);

text(500,100,['Select points near bottom left and top right of'...

' square. Press Enter once finished.'],'Color','red','FontSize',18);

axis on

fprintf(['Select points near bottom left and top right of'...

' square. Press Enter once finished.\n']);

[xbox,ybox]=getpts;

close(fig)

if xbox(1) > xbox(2)

temp=xbox(1);

xbox(1)=xbox(2); xbox(2)=temp;

end

if ybox(1) > ybox(2)

temp=ybox(1);

ybox(1)=ybox(2); ybox(2)=temp;

end

xbox(1)=round(xbox(1))-p.Results.expand_box; xbox(2)=round(xbox(2))+p.Results.expand_box;

ybox(1)=round(ybox(1))-p.Results.expand_box; ybox(2)=round(ybox(2))+p.Results.expand_box;

BinaryImage = imbinarize(GrayImage(ybox(1):ybox(2),xbox(1):xbox(2)));

[labeledImage, numBlobs] = bwlabel(BinaryImage);

[YPixbox,XPixbox]=find(labeledImage==1);

LowerLeftCornerBox=[min(XPixbox)+xbox(1),min(YPixbox)+ybox(1)]; % bottom left hand corner box (as shown in imshow)

cm=max(XPixbox)-min(XPixbox); rm=max(YPixbox)-min(YPixbox);

scale= sqrt(numel(XPixbox)) ; % scaling, pixels per cm

BoxPos=[LowerLeftCornerBox(1),LowerLeftCornerBox(2),cm,rm];

end

function [masks,xPetrieCenter,yPetrieCenter]=create_mask(GrayImage,im_sz,p)

%Select center of Petri dish

fig = figure('position', [0, 0, 1000, 1000]);

imshow(GrayImage);

text(500,100,['Select center of Petri dish'...

'. Press Enter once finished.'],'Color','red','FontSize',18);

axis on

fprintf(['Select center of Petri dish'...

'. Press Enter once finished.\n']);

[xPetrieCenter,yPetrieCenter]=getpts;

close(fig)

% create polar coords for mask

y=imresize(1:im_sz(1),[im_sz(2),im_sz(1)]);

y=transpose(y);

x=imresize(1:im_sz(2),[im_sz(1),im_sz(2)]);

masks=ones(im_sz(1),im_sz(2),'uint8');

MaskBoundary=p.Results.PetriDishDiam/2-p.Results.rad_low;

temp=ones(im_sz(1),im_sz(2),'uint8');

r=((y-yPetrieCenter).^2+(x-xPetrieCenter).^2).^0.5;

temp(find(r> MaskBoundary ))=0;

masks(:,:)=temp;

end

function thresholdLevel=find_threshold(GrayImageMasked,masks,p,xPetrieCenter,yPetrieCenter)

%Select media to calculate background level for threshold

if strcmp(p.Results.manualSelect,'yes')

[row,col]=find(masks ~= 0);

low_bound=[min(row),min(col)];

up_bound=[max(row),max(col)];

fig=figure('position', [0, 0, 1000, 1000]);

imshow(GrayImageMasked(low_bound(1):up_bound(1),low_bound(2):up_bound(2)),[0 80])

text(50,50,['Draw box over media to set background'...

'. Double click in the box once finished.'],'Color','red','FontSize',16);

h = imrect;

position = wait(h);

boxVals=h.getPosition;

mediaBox=GrayImageMasked(low_bound(1)+boxVals(2):low_bound(1)+...

boxVals(2)+boxVals(4),low_bound(2)+boxVals(1):low_bound(2)+boxVals(1)+boxVals(3));

close(fig)

boxMean=median(mediaBox(:));

boxSTD=std(double(mediaBox(:)));

else

mediaBox=GrayImageMasked(fix(yPetrieCenter)-p.Results.mediaBoxLen:fix(yPetrieCenter)+p.Results.mediaBoxLen,...

fix(xPetrieCenter)-p.Results.mediaBoxLen:fix(xPetrieCenter)+p.Results.mediaBoxLen);

if strcmp(p.Results.debugPlot,'yes')

fig_debug=figure('position', [0, 0, 500, 500],'visible','off');

image(mediaBox);

text(1,1,'Region selected for background calcution','Color','red','FontSize',16);

colormap gray

saveas(fig_debug,'media_region_debug.png')

close(fig_debug);

end

Gaussian=@(x,p) p(1)*exp(-(x-p(2)).^2/2./p(3))+p(4);

%doubleGauss=@(x,p) p(1)*exp(-(x-p(2)).^2/2./p(3))+p(4)*exp(-(x-p(5)).^2/2./p(6))+p(7);

objFn=@(q,x,data) sum( (data-Gaussian(x,q)).^2);

histfig=figure('visible','off');

h=histogram(mediaBox(:));

xvals=h.BinEdges(1:h.NumBins)+h.BinWidth/2;

yvals=h.Values;

close(histfig);

[peakHeight,loc]=findpeaks(yvals);

[peakHeightd,locdesc]=sort(peakHeight,'descend');

peaklocs=loc( locdesc(1) );

xvalred=xvals(max([peaklocs-10,1]):min([peaklocs+10,length(xvals)]));

yvalred=yvals(max([peaklocs-10,1]):min([peaklocs+10,length(xvals)]));

guessParam=[peakHeightd(1),xvalred(11),2,min(yvalred)];

options = optimoptions('fsolve','Display','none','Algorithm','levenberg-marquardt');

f= @(p) objFn(p,xvalred,yvalred);

[param,fval,exitflag,output]=fsolve(f, guessParam ,options);

if strcmp(p.Results.debugPlot,'yes')

fig_debug=figure('visible','off');

plot(xvalred,yvalred,'r');

hold on

plot(xvalred,Gaussian(xvalred,param),'b' )

saveas(fig_debug,'media_region_fit_debug.png')

hold off

close(fig_debug);

end

boxMean=param(2);

boxSTD=sqrt(param(3));

end

maxGray=max(GrayImageMasked(:));

thresholdLevel=(double(boxMean)+p.Results.StandDev*boxSTD)/double(maxGray);

end

function [blob_sizes,labeledImage]=choose_blobs(numBlobs,labeledImage,p)

%find large blobs and order

blob_sizes=[];

for i=1:numBlobs

[r,c]=find(labeledImage==i);

if numel(r) > p.Results.min_blob

blob_sizes=[blob_sizes; [numel(r),min(r),min(c),i]];

else

labeledImage(r,c)=0;

end

end

[vals,index]=sort(blob_sizes(:,1),'descend');

blob_sizes=blob_sizes(index,:);

end

function plot_blob_images(GrayImage,labeledImage,BinaryImage,BoxPos,DateString,blob_sizes,ThisFile,xPetrieCenter,yPetrieCenter,p)

[numBlobsLarge,na]=size(blob_sizes);

sav_fig=figure('visible','off');

fig_pos = get(sav_fig,'Position');

fig_pos(3)=fig_pos(3).*3.;

set(sav_fig,'Position',fig_pos);

sav_fig_axa = axes('Position',[0.075+0*(0.05+0.25) 0.05 0.25 0.8]);

sav_fig_axb = axes('Position',[0.075+1*(0.05+0.25) 0.05 0.25 0.8]);

sav_fig_axc = axes('Position',[0.075+2*(0.05+0.25) 0.05 0.25 0.8]);

%original image

image(sav_fig_axa,GrayImage);

title(sav_fig_axa,ThisFile,'FontSize',18,'FontWeight','Bold');

rectangle(sav_fig_axa,'Position',BoxPos,'EdgeColor','y','LineWidth',2);

mediaBoxPos=[xPetrieCenter-p.Results.mediaBoxLen,yPetrieCenter-p.Results.mediaBoxLen,...

2.*p.Results.mediaBoxLen,2.*p.Results.mediaBoxLen];

rectangle(sav_fig_axa,'Position',mediaBoxPos,'EdgeColor','g','LineWidth',1);

axis off

coloredLabels = label2rgb (labeledImage, 'hsv', 'k', 'shuffle');

image(sav_fig_axb,coloredLabels);

for i=1:numBlobsLarge

radius=(blob_sizes(i,1)/pi)^0.5;

XCenter=blob_sizes(i,3)+radius;

YCenter=blob_sizes(i,2)+radius;

text(sav_fig_axa,XCenter,YCenter,num2str(i),'Color','red','FontSize',14);

text(sav_fig_axb,XCenter,YCenter,num2str(i),'Color','red','FontSize',14) ;

end

axis off

image(sav_fig_axc,double(GrayImage).*imcomplement(BinaryImage))

axis off

colormap gray

saveas(sav_fig,strcat('blob_pictures_',strrep(ThisFile,'.tif','_'),DateString,'.png'))

end

STOP COPYING.
