# Supplementary material for: Calculating metalation in cells reveals CobW acquires CoII for vitamin B12 biosynthesis while related proteins prefer ZnII
Source: Nat Commun. 2021 Feb 19;12:1195. doi: 10.1038/s41467-021-21479-8 (PMC7895991; doi:10.1038/s41467-021-21479-8)
Supplement: Supplementary file 7 — Supplementary Software 2 [file 41467_2021_21479_MOESM7_ESM.zip › Supplementary Software 2.docx]

**Supplementary Software 2**

**Dynafit Scripts**

**Dynafit script to describe competition between EGTA and Mg^II^GMPPNP-CobW for Co^II^**

[model]

EGTA binds 1 Co(II) per monomer. Mg(II)GMPPNP-CobW binds 1 Co(II) per monomer.

[components]

; C = EGTA (competitor)

; P = CobW (protein)

; M = Co(II) (metal)

[task]

task = fit

data = equilibria

[mechanism]

C + M <==> MC : Keq1 dissociation

P + M <==> MP : Keq2 dissociation

[concentrations]; micromolar

C = 40

P = 20

[constants]; micromolar

Keq1 = 0.00789

Keq2 = 0.001 ?

[responses]

MP = 0.0028?

[data]

variable M

offset = auto

set MgGMPPNPCobW

[set:MgGMPPNPCobW]

0 0

6 0.008139

12 0.016892

18 0.022708

24 0.029845

30 0.036144

36 0.043848

48 0.052472

60 0.054842

72 0.055745

84 0.056828

96 0.05663

[end]

**Dynafit script to describe competition between EGTA and Mg^II^GTPγS-CobW for Co^II^**

[model]

EGTA binds 1 Co(II) per monomer. Mg(II)GTPyS-CobW binds 1 Co(II) per monomer.

[components]

; C = EGTA (competitor)

; P = CobW (protein)

; M = Co(II) (metal)

[task]

task = fit

data = equilibria

[mechanism]

C + M <==> MC : Keq1 dissociation

P + M <==> MP : Keq2 dissociation

[concentrations]; micromolar

C = 1000

P = 20

[constants]; micromolar

Keq1 = 0.00789

Keq2 = 0.000001 ?

[responses]

MP = 0.0028

[data]

variable M

offset = auto

set MgGTPySCobW

[set:MgGTPySCobW]

0 0

5 0.006432

10 0.010445

15 0.014342

20 0.017468

25 0.019309

30 0.021115

40 0.023736

[end]

**Dynafit script to describe competition between Fura-2 and CobW for Co^II^**

[model]

Fura2 binds 1 Co(II) per monomer. CobW binds 1 Co(II) per monomer.

[components]

; C = Fura2 (competitor)

; P = CobW (protein)

; M = Co(II) (metal)

[task]

task = fit

data = equilibria

[mechanism]

C + M <==> MC : Keq1 dissociation

P + M <==> MP : Keq2 dissociation

[concentrations]; micromolar

P = 37

C = 10

[constants]; micromolar

Keq1 = 0.00864

Keq2 = 0.5 ?

[responses]

MC = -1 ?

[data]

variable M

offset = auto

set CobW

[set:CobW]

0.000 1

2.000 0.786898009

4.000 0.616778285

6.000 0.496297227

8.000 0.399378345

10.000 0.338180665

14.000 0.242332963

18.000 0.192537668

26.000 0.125832535

34.000 0.093327689

50.000 0.048680581

66.000 0.032373569

82.000 0.041403781

[end]

**Dynafit script to describe competition between Fura-2 and Mg^II^GDP-CobW for Co^II^**

[model]

Fura-2 binds 1 Co(II) per monomer. Mg(II)GDP-CobW binds 1 Co(II) per monomer.

[components]

; C = Fura-2 (competitor)

; P = CobW (protein)

; M = Co(II) (metal)

[task]

task = fit

data = equilibria

[mechanism]

C + M <==> MC : Keq1 dissociation

P + M <==> MP : Keq2 dissociation

[concentrations]; micromolar

C = 8.06

P = 20

[constants]; micromolar

Keq1 = 0.00864

Keq2 = 0.01 ?

[responses]

MC = -1 ?

[data]

variable M

offset = auto

set MgGDPCobW

[set:MgGDPCobW]

0 1

2 0.870760656

4 0.670936169

6 0.495969137

8 0.359041645

10 0.26705313

12 0.197223607

15 0.154501562

18 0.116076522

22 0.07842911

26 0.060364067

30 0.047021687

34 0.040090394

38 0.035768074

42 0.031218785

46 0.028668037

[end]

**Dynafit script to describe competition between EGTA and Mg^II^GTP-CobW for Co^II^**

[model]

EGTA binds 1 Co per monomer. Mg(II)GTP-CobW binds 1 Co(II) per monomer.

[components]

; C = EGTA (competitor)

; P = CobW (protein)

; M = Co(II) (metal)

[task]

task = fit

data = equilibria

[mechanism]

C + M <==> MC : Keq1 dissociation

P + M <==> MP : Keq2 dissociation

[concentrations]; micromolar

C = 2000

P = 18

[constants]; micromolar

Keq1 = 0.00789

Keq2 = 0.0001 ?

[responses]

MP = 0.0028

[data]

variable M

offset = auto

set MgGTPCobW

[set:MgGTPCobW]

0 0

4 0.007629

8 0.015819

12 0.02097

16 0.025836

20 0.029532

24 0.031528

34 0.03799

[end]

**Dynafit script to estimate extinction coefficient for Fe^II^Tar_2_ complex at 720 nm**

[model]

2 Tar monomers bind 1 Fe(II) ion.

Estimate extinction coefficient at 720 nm.

[components]

; C = Tar (competitor)

; M = Fe(II) (metal)

[task]

task = fit

data = equilibria

[mechanism]

C + C + M <==> MC2 : Keq1 dissociation

[concentrations]; molar

C = 16e-6

[constants]; molar

Keq1 = 2.51e-14

[responses]

MC2 = 19000 ?

[data]

variable M

offset = auto

set 720

[set:720]

0 0

0.000002 0.042471

0.000004 0.083526

0.000006 0.126292

0.000008 0.152762

0.00001 0.152132

0.000012 0.152632

0.000016 0.149657

0.00002 0.149805

[end]

**Dynafit script to describe competition between Tar and Mg^II^GTP-CobW for Fe^II^**

[model]

2 Tar monomers bind 1 Fe(II) ion.

Mg(II)GTP-CobW binds 1 Fe(II) per monomer.

[components]

; C = TAR (competitor)

; P = CobW (protein)

; M = Co(II) (metal)

[task]

task = fit

data = equilibria

[mechanism]

C + C + M <==> MC2 : Keq1 dissociation

P + M <==> MP : Keq2 dissociation

[concentrations]; molar

C = 16e-6

P = 50e-6

[constants]; molar

Keq1 = 2.512e-14

Keq2 = 0.000001 ?

[responses]

MC2 = 19560

[data]

variable M

offset = auto

set MgGTPCobW

[set:MgGTPCobW]

0 0

0.000002 0.036798

0.000004 0.077242

0.000006 0.120745

0.000008 0.141377

0.00001 0.146101

0.000012 0.148976

0.000016 0.149556

0.00002 0.150987

0.00003 0.152325

0.00004 0.153229

0.00006 0.152552

0.00008 0.151422

[end]

**Dynafit script to determine extinction coefficient for Ni^II^Tar_2_ complex at 535 nm**

[model]

2 Tar monomers bind 1 Ni(II) ion.

Fit extinction coefficient at 535 nm.

[components]

; C = Tar (competitor)

; M = Ni(II) (metal)

[task]

task = fit

data = equilibria

[mechanism]

C + C + M <==> MC2 : Keq1 dissociation

[concentrations]; molar

C = 34e-6

[constants]; molar

Keq1 = 2.3e-16

[responses]

MC2 = 35000 ?

[data]

variable M

offset = auto

set 535

[set:535]

0 0

0.0000033 0.1257

0.0000066 0.248

0.0000099 0.3727

0.0000132 0.4883

0.0000165 0.6058

0.0000198 0.6472

0.0000231 0.6644

0.0000264 0.6636

0.0000297 0.6622

0.000033 0.6607

[end]

**Dynafit script to describe competition between Tar and Mg^II^GTP-CobW for Ni^II^**

[model]

TAR forms 1:2 complex with 1 Ni. CobW binds approx 1 equivalents of Ni with affinity to compete with TAR.

[components]

; C = TAR (competitor)

; P = CobW (protein)

; M = Co(II) (metal)

[task]

task = fit

data = equilibria

[mechanism]

C + C + M <==> MC2 : Keq1 dissociation

P + M <==> MP : Keq2 dissociation

[concentrations] ;molar

C = 20e-6

P = 30e-6

[constants] ;molar

Keq1 = 2.3e-16

Keq2 = 0.000001 ?

[responses]

MC2 = 35000 ?

[data]

variable M

offset = auto

set CobW1

[set:CobW1]

0 0

0.000003 0.103169

0.000006 0.198488

0.000009 0.253271

0.000012 0.278219

0.000015 0.299028

0.000018 0.308315

0.000022 0.311758

0.000026 0.325144

0.00003 0.332409

0.000034 0.336594

0.000038 0.350309

0.000042 0.355477

[end]

**Dynafit script to describe competition between Bca and Mg^II^GTP-CobW for Cu^I^**

[model]

2 Bca monomers bind 1 Cu(I) ion. Mg(II)GTP-CobW binds 1 Cu(I) per monomer.

[components]

; C = Bca (competitor)

; P = CobW (protein)

; M = Cu(I) (metal)

[task]

task = fit

data = equilibria

[mechanism]

C + C + M <==> MC2 : Keq1 dissociation

P + M <==> MP : Keq2 dissociation

[concentrations]; molar

C = 1000e-6

P = 20e-6

[constants]; molar

Keq1= 6.3e-18

Keq2 = 0.0000000000000001 ?

[responses]

MC2 = 7900

[data]

variable M

offset = auto

set MgGTPCobW

[set:MgGTPCobW]

0 0

0.00000336 0.013419

0.00000672 0.028628

0.00001008 0.045342

0.00001344 0.060609

0.0000168 0.077346

0.0000224 0.10741

0.000028 0.140089

0.0000336 0.171832

[end]

**Dynafit script to describe competition between Tar and Mg^II^GTP-YeiR for Fe^II^**

[model]

YeiR binds 1 Fe per monomer. TAR binds 1 Fe per 2 molecules

[components]

; F = Fe

; T = TAR

; P = protein

[task]

task = fit

data = equilibria

[mechanism]

F + T + T <==> FT2 : Keq1 association

F + P <==> FP : Keq2 association

[concentrations] ;molar

T = 0.0000153

P = 0.000010

[constants] ;molar

Keq1 = 39800000000000

Keq2 = 3980000 ?

[responses]

FT2 = 19000

[data]

variable F

offset = auto

set MgGTPYeiR

[set:MgGTPYeiR]

0 0.000897

1.06667E-06 0.021332

2.13333E-06 0.044404

0.0000032 0.066263

4.26667E-06 0.08671

5.33333E-06 0.10755

0.0000064 0.126339

7.46667E-06 0.141943

8.53333E-06 0.148718

0.0000096 0.150257

1.06667E-05 0.149921

0.0000128 0.150551

[end]

**Dynafit script to describe competition between Fura-2 and Mg^II^GTP-YeiR for Co^II^**

[model]

YeiR binds 1 Co per monomer. Fura2 binds 1 Co per molecule

[components]

; P = protein monomer

; C = Co

; F = Fura2

[task]

task = fit

data = equilibria

[mechanism]

F + C <==> FC : Keq1 dissociation

P + C <==> PC : Keq2 dissociation

[concentrations] ;micromolar

F = 10.3

P = 8.9

[constants] ;micromolar

Keq1 = 8.64e-3

Keq2 = 8.64e-3 ?

[responses]

FC = -79.5 ?

[data]

variable C

offset = auto

set MgGTPYeiR

[set:MgGTPYeiR]

0 848.7721

0.916 800.6973

1.832 747.1918

2.748 710.3059

3.664 665.1365

4.58 640.4542

5.496 607.2464

6.412 574.9301

7.328 539.0147

8.244 508.7284

9.16 470.68

10.076 436.6915

10.992 390.6033

11.908 343.9182

12.824 299.442

13.74 253.4265

14.656 216.0951

15.572 182.2139

16.488 154.6994

17.404 132.5288

18.32 117.8734

19.694 95.92131

21.068 83.13196

22.9 67.90307

25.648 53.83965

29.312 43.70213

33.892 33.41156

38.472 29.69386

[end]

**Dynafit script to describe competition between Mag-fura-2 and Mg^II^GTP-YeiR for Ni^II^**

[model]

Both molecules bind one nickel atom

[components]

;N = nickel

;M = Mf2

;P = protein

[task]

data = equilibria

task = fit

[mechanism]

M + N <==> MN : Keq1 dissociation

P + N <==> PN : Keq2 dissociation

[constants] ;micromolar

Keq1 = 5e-2

Keq2 = 5e-2 ?

[concentrations] ;micromolar.

M = 10.6

P = 8.9

[equil]

variable N

offset = auto

set data1_325 | response MN = 0.019413 ?

set data1_366 | response MN = -0.01546 ?

[set:data1_325]

0 0.139576

0.907 0.151422

1.814 0.166906

2.721 0.181083

3.628 0.191492

4.535 0.20551

5.442 0.217592

6.349 0.22969

7.256 0.242288

8.163 0.251916

9.07 0.266359

9.977 0.279724

10.884 0.295618

11.791 0.301198

12.698 0.314394

13.605 0.317867

14.512 0.324804

15.419 0.330406

16.326 0.335766

17.233 0.341541

18.14 0.340003

19.047 0.346776

20.861 0.345845

22.675 0.346011

24.489 0.344389

27.21 0.350291

30.838 0.347649

34.466 0.348112

38.094 0.345356

[set:data1_366]

0 0.202081

0.907 0.194007

1.814 0.189919

2.721 0.18384

3.628 0.174986

4.535 0.167212

5.442 0.158462

6.349 0.150333

7.256 0.142539

8.163 0.132514

9.07 0.121216

9.977 0.112099

10.884 0.107069

11.791 0.093336

12.698 0.088135

13.605 0.076535

14.512 0.069777

15.419 0.065637

16.326 0.061649

17.233 0.061567

18.14 0.05455

19.047 0.056359

20.861 0.048886

22.675 0.044652

24.489 0.0407

27.21 0.044692

30.838 0.039491

34.466 0.039921

38.094 0.038159

[end]

**Dynafit script simulating competition between Mg^II^GTP-YeiR and Bca for Cu^I^ from calculated affinity of Mg^II^GTP-YeiR**

[model]

YeiR binds 1 Cu per monomer. BCA binds 1 Cu per 2 molecule

[components]

; C = Cu

; B = BCA

; P = protein

[task]

task = fit

data = equilibria

[mechanism]

C + B + B <==> CB2 : Keq1 association

C + P <==> CP : Keq2 association

[concentrations] ;molar

B = 0.000800

P = 0.00001

[constants] ;molar

Keq1 = 158000000000000000

Keq2 = 2.03e15

[responses]

CB2 = 7900

[data]

variable C

set Bca

[set:Bca]

0 0

5e-6 0.0395

10e-6 0.079

[end]

**Dynafit script simulating binding of Cu^I^ to Bca in the absence of Mg^II^GTP-YeiR**

[model]

BCA binds 1 Cu per 2 molecule

[components]

; C = Cu

; B = BCA

[task]

task = fit

data = equilibria

[mechanism]

C + B + B <==> CB2 : Keq1 association

[concentrations] ;molar

B = 0.000800

[constants] ;molar

Keq1 = 158000000000000000

[responses]

CB2 = 7900

[data]

variable C

set Bca

[set:Bca]

0 0

5e-6 0.0395

10e-6 0.079

[end]

**Dynafit script describing binding of Zn^II^ to quin-2 in the absence of Mg^II^GTP-YeiR**

[model]

Quin2 binds 1 Zn per molecule

[components]

; Z = Zn

; Q = quin2

[task]

task = fit

data = equilibria

[mechanism]

Q + Z <==> QZ : Keq1 dissociation

[concentrations] ;micromolar

Q = 10 ?

[constants] ;micromolar

Keq1 = 3.7e-6

[responses]

QZ = -0.1 ?

[data]

variable Z

offset = auto

set quin2

[set:quin2]

0 1

0.89 0.865703907

1.78 0.729399103

2.67 0.633194316

3.56 0.510764934

4.45 0.424766075

5.34 0.323745947

6.23 0.218063564

7.12 0.134379596

8.01 0.058183067

8.9 0.0331971

9.79 0.017920681

10.68 0.013638831

11.57 0.004889567

12.46 0.000848948

13.35 0

[end]

**Dynafit script describing competition between Mg^II^GTP-YeiR and quin-2 for Zn^II^ with [Mg^II^GTP-YeiR] as a fitted parameter**

[model]

Quin2 binds 1 Zn per molecule. Protein binds 1 Zn per molecule.

[components]

; Z = Zn

; Q = quin2

; P = protein

[task]

task = fit

data = equilibria

[mechanism]

Q + Z <==> QZ : Keq1 dissociation

P + Z <==> PZ : Keq2 dissociation

[concentrations] ;micromolar

Q = 7.73

P = 10 ?

[constants] ;micromolar

Keq1 = 3.7e-6

Keq2 = 1e-6 ?

[responses]

QZ = -0.127

[data]

variable Z

offset = auto

set MgGTPYeiR

[set:MgGTPYeiR]

0 1

0.89 1.026045343

1.78 0.885676112

2.67 0.840198649

3.56 0.777137743

4.45 0.790536453

5.34 0.630167894

6.23 0.560682834

7.12 0.527116736

8.01 0.450984841

8.9 0.394062384

9.79 0.320207724

10.68 0.234290444

11.57 0.178498202

12.46 0.153259554

13.35 0.061443313

14.24 0.01689439

15.13 0.01438187

16.02 0

16.91 0.01187355

[end]

**Dynafit script describing competition between Mg^II^GTP-YeiR and quin-2 for Zn^II^ with [Mg^II^GTP-YeiR] as a fixed parameter**

[model]

Quin2 binds 1 Zn per molecule. Protein binds 1 Zn per molecule.

[components]

; Z = Zn

; Q = quin2

; P = protein

[task]

task = fit

data = equilibria

[mechanism]

Q + Z <==> QZ : Keq1 dissociation

P + Z <==> PZ : Keq2 dissociation

[concentrations] ;micromolar

Q = 7.73

P = 10

[constants] ;micromolar

Keq1 = 3.7e-6

Keq2 = 1e-6 ?

[responses]

QZ = -0.127

[data]

variable Z

offset = auto

set MgGTPYeiR

[set:MgGTPYeiR]

0 1

0.89 1.026045343

1.78 0.885676112

2.67 0.840198649

3.56 0.777137743

4.45 0.790536453

5.34 0.630167894

6.23 0.560682834

7.12 0.527116736

8.01 0.450984841

8.9 0.394062384

9.79 0.320207724

10.68 0.234290444

11.57 0.178498202

12.46 0.153259554

13.35 0.061443313

14.24 0.01689439

15.13 0.01438187

16.02 0

16.91 0.01187355

[end]

**Dynafit script describing binding of Zn^II^ to quin-2 in the absence of Mg^II^GTPγS-YeiR**

[model]

Quin2 binds 1 Zn per molecule

[components]

; Z = Zn

; Q = quin2

[task]

task = fit

data = equilibria

[mechanism]

Q + Z <==> QZ : Keq1 dissociation

[concentrations] ;micromolar

Q = 10 ?

[constants] ;micromolar

Keq1 = 3.7e-6

[responses]

QZ = -0.1 ?

[data]

variable Z

offset = auto

set quin2

[set:quin2]

0 1

0.89 0.92777912

1.78 0.837775247

2.67 0.7296203

3.56 0.649339943

4.45 0.573909843

5.34 0.471218622

6.23 0.373239626

7.12 0.275747572

8.01 0.154483515

8.9 0.087227916

9.79 0.055818198

10.68 0.031482568

11.57 0.021015218

12.46 0.008504243

13.35 0

[end]

**Dynafit script describing competition between Mg^II^GTPγS-YeiR and quin-2 for Zn^II^**

[model]

Quin2 binds 1 Zn per molecule. Protein binds 1 Zn per molecule.

[components]

; Z = Zn

; Q = quin2

; P = protein

[task]

task = fit

data = equilibria

[mechanism]

Q + Z <==> QZ : Keq1 dissociation

P + Z <==> PZ : Keq2 dissociation

[concentrations] ;micromolar

Q = 9.61

P = 10 ?

[constants] ;micromolar

Keq1 = 3.7e-6

Keq2 = 1e-6 ?

[responses]

QZ = -0.1017

[data]

variable Z

offset = auto

set MgGTPySYeiR

[set:MgGTPySYeiR]

0 1

0.89 0.942065454

1.78 0.919945194

2.67 0.844239772

3.56 0.836108477

4.45 0.8096687

5.34 0.760813727

6.23 0.683577621

7.12 0.645008101

8.01 0.655861029

8.9 0.587827697

9.79 0.535000411

10.68 0.499772264

11.57 0.452433042

12.46 0.393598752

13.35 0.335974075

14.24 0.263546559

15.13 0.211869153

16.02 0.152635391

17.8 0.116914438

19.58 0.072532051

21.36 0.000694408

23.14 0

[end]

**Dynafit script describing competition between Mg^II^GTPγS-YjiA and mag-fura-2 for Mn^II^**

[model]

Both molecules bind one manganese ion

[components]

;N = manganese

;M = Magfura2

;P = protein

[task]

data = equilibria

task = fit

[mechanism]

M + N <==> MN : Keq1 dissociation

P + N <==> PN : Keq2 dissociation

[constants] ;micromolar

Keq1 = 6.1

Keq2 = 1 ?

[concentrations] ;micromolar.

M = 10.9

P = 10.0

[equil]

variable N

offset = auto

set data1_330 | response MN = 0.01369

set data1_365 | response MN = -0.01455

[set:data1_330]

0 0.19865

1 0.207371

2 0.215152

3 0.222614

4 0.230855

5 0.237535

6 0.245146

7 0.251813

8 0.256969

9 0.262944

10 0.267393

12 0.274863

14 0.282518

16 0.287983

18 0.293901

20 0.297787

[set:data1_365]

0 0.25572

1 0.248918

2 0.240281

3 0.232425

4 0.223226

5 0.214653

6 0.206508

7 0.199564

8 0.194187

9 0.188718

10 0.183187

12 0.173485

14 0.166369

16 0.15908

18 0.154883

20 0.148879

[end]

**Dynafit script describing competition between Mg^II^GTPγS-YjiA and Tar for Fe^II^**

[model]

YjiA binds one Fe per monomer. 2 tar bind 1 Fe.

[components]

; P = protein monomer

; F = Fe

; D = tar

[task]

task = fit

data = equilibria

[mechanism]

D + D + F <==> DDF : Keq1 dissociation

P + F <==> PF : Keq2 dissociation

[concentrations] ;micromolar

D = 15.3 ?

P = 9.1

[constants] ;micromolar

Keq2 = .1 ?

Keq1 = 2.5e-2

[responses]

DDF = .019 ?

[data]

variable F

offset = auto

set data

[set:data]

0 0.038215

1 0.061654

2 0.083831

3 0.109813

4 0.129628

5 0.1519

6 0.171338

7 0.202418

8 0.212797

9 0.215662

10 0.216266

12 0.217008

14 0.216435

[end]

**Dynafit script describing competition Mg^II^GTPγS-YjiA and fura-2 for Co^II^**

[model]

YjiA binds one cobalt per monomer. F2 binds one cobalt molecule.

[components]

; P = protein monomer

; C = Co

; D = F2

[task]

task = fit

data = equilibria

[mechanism]

D + C <==> DC : Keq1 dissociation

P + C <==> PC : Keq2 dissociation

[concentrations] ;micromolar

D = 10.0

P = 9.86

[constants] ;micromolar

Keq2 = .1 ?

Keq1 = 8.6e-3

[responses]

DC = -73 ?

[data]

variable C

offset = auto ?

set data

[set:data]

0 770.3581543

0 776.7072144

1 700.0096436

1 697.8795776

2 626.1061401

2 635.6925049

3 571.5881348

3 566.522522

4 507.7991943

4 508.9021301

5 455.7695007

5 467.5066833

6 396.6525269

6 399.2562866

7 353.6632385

7 355.0593262

8 300.5822449

8 303.43927

9 261.361969

9 258.742157

10 217.4630127

10 216.22229

11 181.299118

11 182.6351929

12 153.8913574

12 153.0696106

13 130.0011444

13 128.6427765

14 108.955574

14 106.2553177

16 80.95066833

16 77.72036743

18 58.26393509

18 56.28638458

20 42.99574661

20 44.98760605

22 36.07785034

22 35.13269043

24 28.56347466

24 28.28633308

[end]

**Dynafit script describing competition between Mg^II^GTP-YjiA and mag-fura-2 for Ni^II^**

[model]

Both molecules bind one nickel atom

[components]

;N = nickel

;M = Magfura2

;P = protein

[task]

data = equilibria

task = fit

[mechanism]

M + N <==> MN : Keq1 dissociation

P + N <==> PN : Keq2 dissociation

[constants] ;micromolar

Keq1 = 5e-2

Keq2 = .01 ?

[concentrations] ;micromolar.

M = 10.5

P = 10.0

[equil]

variable N

offset = auto

set data1_323 | response MN = 0.01924

set data1_365 | response MN = -0.01383

[set:data1_323]

0 0.111261

1 0.125408

2 0.137665

3 0.149307

4 0.165744

5 0.176567

6 0.190587

7 0.197404

8 0.209549

9 0.217584

10 0.229064

11 0.238221

12 0.247422

13 0.255549

14 0.263061

16 0.277491

18 0.290586

20 0.307893

22 0.310373

24 0.31719

[set:data1_365]

0 0.203446

1 0.190048

2 0.180758

3 0.170187

4 0.162479

5 0.151561

6 0.142117

7 0.138465

8 0.130467

9 0.124493

10 0.118067

11 0.112642

12 0.106812

13 0.099302

14 0.093293

16 0.082354

18 0.072914

20 0.073519

22 0.061269

24 0.05572

[end]

**Dynafit script describing competition between Mg^II^GTPγS-YjiA or (Mg^II^GTP-YjiA) and quin-2 for Zn^II^**

[model]

YjiA binds one zinc per monomer. Quin2 binds one zinc per molecule.

[components]

; P = protein monomer

; Z = Zn

; Q = Quin2

[task]

task = fit

data = equilibria

[mechanism]

Q + Z <==> QZ : Keq1 dissociation

P + Z <==> PZ : Keq2 dissociation

[concentrations] ;micromolar

Q = 8.6

P = 10.3

[constants] ;micromolar

Keq1 = 3.7e-6

Keq2 = 1e-7 ?

[responses]

QZ = -0.01783 ?

[data]

variable Z

offset = auto ?

set data

[set:data]

0 1

1.5 0.893509696

3 0.789423831

4.5 0.752802376

6 0.715721332

7.5 0.636352427

9 0.584682211

10.5 0.521701603

12 0.439984307

13.5 0.354338079

15 0.229318462

16.5 0.119560587

18 0.047657213

19.5 0.082188096

21 0.009146957

22.5 0.004354893

24 -0.020143482

25.5 -0.01270037

27 -0.021387737

[end]
