# Supplementary material for: Calculating metalation in cells reveals CobW acquires CoII for vitamin B12 biosynthesis while related proteins prefer ZnII
Source: Nat Commun. 2021 Feb 19;12:1195. doi: 10.1038/s41467-021-21479-8 (PMC7895991; doi:10.1038/s41467-021-21479-8)
Supplement: Supplementary file 8 — Reporting Summary [file 41467_2021_21479_MOESM8_ESM.pdf]

nature research

Corresponding author(s): Tessa R. Young and Nigel J. Robinson

Last updated by author(s): Jan 11, 2021

Reporting Summary

Nature Research wishes to improve the reproducibility of the work that we publish. This form provides structure for consistency and transparency in reporting. For further information on Nature Research policies, see our Editorial Policies and the Editorial Policy Checklist.

Statistics

For all statistical analyses, confirm that the following items are present in the figure legend, table legend, main text, or Methods section.

n/a	Confirmed	
☐	☒	The exact sample size (*n*) for each experimental group/condition, given as a discrete number and unit of measurement
☐	☒	A statement on whether measurements were taken from distinct samples or whether the same sample was measured repeatedly
☒	☐	The statistical test(s) used AND whether they are one- or two-sided<br>*Only common tests should be described solely by name; describe more complex techniques in the Methods section.*
☒	☐	A description of all covariates tested
☒	☐	A description of any assumptions or corrections, such as tests of normality and adjustment for multiple comparisons
☐	☒	A full description of the statistical parameters including central tendency (e.g. means) or other basic estimates (e.g. regression coefficient) AND variation (e.g. standard deviation) or associated estimates of uncertainty (e.g. confidence intervals)
☒	☐	For null hypothesis testing, the test statistic (e.g. *F*, *t*, *r*) with confidence intervals, effect sizes, degrees of freedom and *P* value noted<br>*Give P values as exact values whenever suitable.*
☒	☐	For Bayesian analysis, information on the choice of priors and Markov chain Monte Carlo settings
☒	☐	For hierarchical and complex designs, identification of the appropriate level for tests and full reporting of outcomes
☒	☐	Estimates of effect sizes (e.g. Cohen's *d*, Pearson's *r*), indicating how they were calculated

Our web collection on statistics for biologists contains articles on many of the points above.

Software and code

Policy information about availability of computer code

Data collection	PerkinElmer UV WinLab Version 6.0.3 was used for UV-vis spectroscopy; Cary Eclipse Version 1.1 Scan Application was used for fluorescence spectroscopy; ImageLab Version 5.2.1 (Bio-Rad) was used to collect images of SDS-PAGE gels and B12 bioassay plates; Rotor-Gene Q - Pure Detection Version 2.3.4 (Qiagen) was used to collect qPCR data; PlasmaLab Version 2.6.2.337 (ThermoFisher) was used to collect ICP-MS data.
Data analysis	CCP4 Molecular Graphics Version 2.10.10 was used to produce figure of structural homology model; SigmaPlot Version 14.0 was used to produce graphs; ChemDraw Version 18.1 was used to produce figures of nucleotide structure; Dynafit Version 4 (BioKin Ltd) was used to fit biochemical data (example scripts provided in Supplementary Software 2); Kaleidagraph Version 4.1.1 (Synergy Software) was used to fit biochemical data; mass spectrometry data were processed using Masslynx 4.1 and deconvoluted using MaxEnt 1; LinReg PCR Version 2016.1 was used to analyze qPCR data; MATLAB R2019a Version 9.6.0.1072779 was used to determine buffered metal concentration from DNA occupancy of sensors (using code from ref. 9) and to determine areas of bacterial growth on B12 bioassay plates (using code provided in Supplementary Software 1); Excel version: Microsoft Excel Version 16.36 was used to produce the metalation calculator spreadsheet (Supplementary Data 1).

For manuscripts utilizing custom algorithms or software that are central to the research but not yet described in published literature, software must be made available to editors and reviewers. We strongly encourage code deposition in a community repository (e.g. GitHub). See the Nature Research guidelines for submitting code & software for further information.

Data

Policy information about availability of data

All manuscripts must include a data availability statement. This statement should provide the following information, where applicable:

- Accession codes, unique identifiers, or web links for publicly available datasets
- A list of figures that have associated raw data
- A description of any restrictions on data availability

All source data are available within the article, its Supplementary Information files and Source Data files, or from the corresponding authors upon request.

Field-specific reporting

Please select the one below that is the best fit for your research. If you are not sure, read the appropriate sections before making your selection.

☒ Life sciences ☐ Behavioural & social sciences ☐ Ecological, evolutionary & environmental sciences

For a reference copy of the document with all sections, see nature.com/documents/nr-reporting-summary-flat.pdf

Life sciences study design

All studies must disclose on these points even when the disclosure is negative.

Sample size	Sample sizes were chosen based on prior experimental experience, and to give consistent results, following convention in the literature for equivalent analyses (eg, ref. 9 in main manuscript). Experiments designed to derive quantitative values used to model or test calculations of metalation were performed in triplicate (n=3) to enable calculation of SD (listed in Tables or shown as error bars in Figures) with additional replicates (up to n=5) performed when the SD was initially high. A fourth replicate in which Zn(II) was added before Co(II) (rather than the reverse) was also performed in the determination of Zn(II) affinity of MgGTP-CobW. The number of independent experiments or biologically independent samples is shown in Figure legends or footnotes of Tables.
Data exclusions	Metal competition data were excluded from affinity calculations in cases when the experimental design led to no measurable equilibrium (ie negligible partitioning of metal to one or other partner: protein or competing ligand) and would therefore lead to unreliable affinity estimations (see examples in Supplementary Figure 4j-k). The limiting affinities inferred in these cases were consistent with the final calculated affinities (determined from experiments where measurable equilibrium was observed). This exclusion criteria was pre-established.
Replication	Replicates (‘n’), and their nature (n always refers to independent experiments and/or biologically independent samples, not technical replicates) are specifically defined in the Figure legends or Table footnotes. For quantitative data used to model and test in vivo protein metalation ‘n’ was 3-5 to enable calculation of s.d.
Randomization	Data collection by instruments (absorbance, fluorescence, ICP-MS, qPCR, image capture and analysis, cell-counting) was independent from the experimenter when collected therefore randomisation was not performed. Bacterial colonies used for protein purification, and gene expression analyses were chosen at random.
Blinding	No blinding was used in this study as the types of measurements made (see randomisation) were not susceptible to subjective bias. Automatic analysis of colony size (Supplementary Software 1) was developed to meet this requirement in the assays of B12.

Reporting for specific materials, systems and methods

We require information from authors about some types of materials, experimental systems and methods used in many studies. Here, indicate whether each material, system or method listed is relevant to your study. If you are not sure if a list item applies to your research, read the appropriate section before selecting a response.

Materials & experimental systems

n/a	Involved in the study	
☒	☐	Antibodies
☒	☐	Eukaryotic cell lines
☒	☐	Palaeontology and archaeology
☒	☐	Animals and other organisms
☒	☐	Human research participants
☒	☐	Clinical data
☒	☐	Dual use research of concern

Methods

n/a	Involved in the study	
☒	☐	ChIP-seq
☒	☐	Flow cytometry
☒	☐	MRI-based neuroimaging
